# Comprehensive investigation of the gene expression system regulated by an *Aspergillus oryzae* transcription factor XlnR using integrated mining of gSELEX-Seq and microarray data

**DOI:** 10.1186/s12864-018-5375-5

**Published:** 2019-01-08

**Authors:** Hiroya Oka, Takaaki Kojima, Kunio Ihara, Tetsuo Kobayashi, Hideo Nakano

**Affiliations:** 1 Department of Applied Biosciences, Graduate School of Bioagricultural Sciences, Nagoya University, Furo-cho, Chikusa-ku, Nagoya, 464-8601 Japan; 2 Center for Gene Research, Nagoya University, Furo-cho, Chikusa-ku, Nagoya, 464-8602 Japan

**Keywords:** *Aspergillus oryzae*, gSELEX-Seq (genomic SELEX-Seq), XlnR, Transcriptome

## Abstract

**Background:**

Transcription factors (TFs) specifically bind to DNA sequences and control the expression of target genes. AoXlnR is a key TF involved in the expression of xylanolytic and cellulolytic enzymes in the filamentous fungi, *Aspergillus oryzae*. Genomic SELEX-Seq (gSELEX-Seq) can reveal the in vitro binding sites of a TF in a genome. To date, the gene expression network controlled by AoXlnR in *A. oryzae* is not fully explored. In this study, the data from gSELEX-Seq analysis and data mining were applied toward a comprehensive investigation of the AoXlnR-regulated transcriptional network in *A. oryzae*.

**Results:**

Around 2000 promoters were selected as AoXlnR-binding DNAs using gSELEX-Seq, consequently identifying the genes downstream of them. On the other hand, 72 differentially expressed genes (DEGs) related to AoXlnR had been determined by microarray analysis. The intersecting set of genes, that were found using the gSELEX-Seq and the microarray analysis, had 51 genes. Further, the canonical AoXlnR-binding motifs, 5′-GGCT(A/G) A-3′, were successfully identified in gSELEX-Seq. The motif numbers in each promoter of the DEGs and differential expression levels were correlated by in silico analysis. The analysis showed that the presence of both 5′-GGCTAA-3′ and 5′-GGCTGA-3′ motif has significantly high correlation with the differential expression levels of the genes.

**Conclusions:**

Genes regulated directly by AoXlnR were identified by integrated mining of data obtained from gSELEX-Seq and microarray. The data mining of the promoters of differentially expressed genes revealed the close relation between the presence of the AoXlnR-binding motifs and the expression levels of the downstream genes. The knowledge obtained in this study can contribute greatly to the elucidation of AoXlnR-mediated cellulose and xylan metabolic network in *A. oryzae*. The pipeline, which is based on integrated mining of data consisting of both in vitro characterization of the DNA-binding sites and TF phenotype, can be a robust platform for comprehensive analysis of the gene expression network via the TFs.

**Electronic supplementary material:**

The online version of this article (10.1186/s12864-018-5375-5) contains supplementary material, which is available to authorized users.

## Background

Transcription factors (TFs) interact with the enhancers and promoters on a genome and play a central role in transcription regulation in cells [1]. Identifying the genes regulated by a TF is important for understanding not only the function of the TF but also the gene expression network of the cells. However, since laborious efforts are required to comprehensively identify TF-regulated genes, except for some model TFs, the detailed functions of most TFs are still poorly understood [2, 3]. Therefore, so far, various methods have been devised for high-throughput identification of genes regulated by a target TF.

DNA microarray (DNA-chip) analysis, which is a comprehensive DNA hybridization technique, was proposed by Brawn et al. [4]. This technique is mainly applied for simultaneous analysis of the expression of more than thousands of genes. To date, many genes regulated by TFs have been identified using this method in various organisms [5, 6]. However, since DNA microarray analysis gives no information of the TF-binding sites, it is very difficult to distinguish whether the expression of the differentially expressed genes (DEGs) is regulated directly or indirectly by a target TF.

On the other hand, systematic evolution of ligands by exponential enrichment (SELEX) is a method used for selecting nucleotides that specifically bind to a target from a random sequence pool in vitro [7–9]. Genomic SELEX (gSELEX), in which the binding sites of a target TF can be preferentially enriched from a genomic library in vitro, enables direct mapping of the TF-binding sites within the genome [10]. Dror et al. showed that the form of DNA near the consensus motif contributes to the determinant of the TF-binding sequence [11]. The study suggested that it is important to use genomic libraries rather than synthetic DNA libraries for the identification of TF-binding sequences. Recently, Kojima et al. established a new comprehensive analysis system for transcriptional regulatory networks by using gSELEX-Seq and RNA-Seq [12]. This system identified the genes directly regulated by AmyR, a TF from *A. nidulans* [12]. The analytical pipeline is a robust platform for comprehensive genome-wide identification of genes regulated by a target TF.

*A. oryzae* is one of the most widely used industrial microorganisms in food production and brewing since ancient times in Asia. Currently, this fungus is expected to have further industrial applications such as the production of useful proteins or metabolites, not only because of its high protein-production ability but also the safety for human health and the environment [13]. In addition, since a variety of cellulolytic and xylanolytic enzymes can be produced in *A. oryzae*, this is an attractive microorganism for biofuel production from plant biomass, which is mainly composed of cellulose, hemicelluloses, and lignin [14]. To utilize the xylanolytic or cellulolytic pathway of *A. oryzae* for such applications, it is extremely important to understand the transcriptional network controlled by TFs.

Filamentous fungi can produce various enzymes in the presence of cellulose or β-1,4 xylan. XlnR has been identified as a TF involved in the expressions of xylanolytic and cellulolytic enzymes in *Aspergillus niger* [15–18]. The XlnR (AnXlnR) binds to 5´-GGCTAAA-3′ and 5´-GGCTAG-3′ via the Zn (II)_2_ Cys_6_ zinc finger motif [17, 19]. AoXlnR, an ortholog of XlnR in *A. oryzae*, also controls the expression of xylanolytic and cellulolytic genes [20–22]. The binding sites of AoXlnR have been presumed to be 5´-GGCTAA-3′ and 5´-GGCTGA-3′ by electrophoretic mobility shift assay (EMSA) using AoXlnR [20–22].

In this study, the genes directly regulated by AoXlnR were successfully identified by combining data obtained from gSELEX-Seq and microarray analyses [22]. The AoXlnR-binding motifs extracted from the selected DNA sequences by gSELEX-Seq were consistent with the canonical motifs reported in the previous study [21]. Furthermore, correlations between the motifs in promoters of the DEGs and the differential expression levels were analyzed by using bioinformatics data mining. Finally, the results obtained from the comprehensive analysis in this study are discussed.

## Methods

### Construction of an *A. oryzae* genomic library

*A. oryzae* genomic library was constructed as described previously [12], with some modifications. A wild type *A. oryzae* strain, RIB40 (National Research Institute of Brewing Stock Culture ATCC-42149) was used to isolate genomic DNA in this study. Linkers were prepared by annealing the corresponding primer pairs: Nextra-Read1/Nextra-Read1-adp-comp and Nextra-Read2/Nextra-Read2-adp-comp (Additional file 1: Table S1). One nanogram of genomic DNA that was size-fractionated to approximately 100 bp was amplified in a 20-μL PCR mixture containing 0.025 U/μL *LA Taq* (Takara Bio, Otsu, Japan) and 0.25 μM of each of the primers Nextra-Read1 and Nextra-Read2 (Additional file 1: Table S1) in the following PCR program: preheating at 94 °C for 3 min; 15 cycles consisting of 94 °C for 15 s and 68 °C for 2 min; and additional extension at 72 °C for 7 min. The amplicons were purified using MinElute PCR Purification Kit (Qiagen, Tokyo, Japan) for gSELEX analysis.

### Expression of recombinant AoXlnR in *Escherichia coli*

The N-terminal 183 amino acids of AoXlnR that included the DNA-binding domain was expressed as a MalE (maltose-binding protein [MBP]) fusion protein in *E. coli* BL21(DE3) [12, 21], followed by purification according to the procedure described in a previous study [23]. The purified protein concentration was determined with the Bradford assay, with bovine serum albumin (BSA) as a standard [24].

### In vitro selection of AoXlnR-bound DNA fragments from the *A. oryzae* genomic library using gSELEX-Seq

gSELEX was performed according to the procedure described in a previous study [12], with some modifications. In this study, 20 ng of *A. oryzae* genomic library was mixed with 100 μL of 0.4 μM purified MBP-XlnR_1–183_ in PBS (137 mM NaCl, 2.7 mM KCl, 10 mM Na_2_HPO_4_, 1.8 mM KH_2_PO_4_, pH 7.4) containing 10 mM 2-mercaptoethanol (PBS/2-ME) and agitated for 30 min at room temperature. Next, 10 μL of amylose resin (New England BioLabs, Ipswich, MA, USA) was washed with 500 μL of MBP without (w/o) EDTA buffer (200 mM NaCl, 20 mM Tris–HCl, 10 mM 2-mercaptoethanol, pH 7.5) and then, the resin was suspended in a 1.5-mL tube in 900 μL of fresh MBP w/o EDTA buffer and mixed with the MBP-XlnR_1–183_-binding reaction mixture. The suspension was mixed using a rotator for 1 h at 4 °C, following which the resin was recovered by centrifuging the suspension at 300×*g* for 1 min at 4 °C. The MBP-XlnR_1–183_-bound amylose resin was washed with 500 μL of MBP w/o EDTA buffer. After removing as much of the supernatant as possible, the resin was suspended in 10 μL of MBP w/o EDTA elution buffer (200 mM NaCl, 20 mM Tris–HCl, 10 mM 2-mercaptoethanol, 20 mM maltose, pH 7.5), and the suspension was mixed using a rotator for 15 min at 4 °C. Lastly, the supernatant was recovered after centrifugation at 300×*g* for 1 min at 4 °C.

The selected DNA fragments in the supernatant were amplified using a PCR mixture (8 tubes × 20 μL) that included 0.025 U/μL *LA* Taq DNA polymerase (Takara) and 0.25 μM primers (Nextra-Read1 and Nextra-Read2) in the following program: preheating at 95 °C for 3 min; 14 cycles (in the first round), 12 cycles (in the second round), or 10 cycles (in the third round) of 94 °C for 15 s and 68 °C for 2 min; and an additional extension at 72 °C for 7 min.

### Analysis of relative AoXlnR-binding affinity by using bead display and flow cytometry

The selected DNA fragments from gSELEX (from each round) were PCR-amplified using the primers Nextra-Read1-bio and Nextra-Read2-Cy5, followed by purification using MinElute PCR Purification Kit for the immobilization of M-280 streptavidin-coated beads (Dynabeads M-280 Streptavidin; Life Technologies, Carlsbad, CA, USA). The relative AoXlnR-binding activity of the fragments in each pool selected by gSELEX was examined by using flow cytometry (JSAN; Bay Bioscience, Kobe, Japan) as described by Kojima et al. [12]. The flow cytometric data were analyzed by using the FlowJo software (Treestar, Ashland, OR, USA).

### DNA sequencing and data analysis in gSELEX-Seq

For sequencing with an Illumina MiSeq sequencer (Illumina, San Diego, CA, USA), the selected DNA fragments by gSELEX (from each round) were purified using the Agencourt AMPure XP system (Beckman Coulter, Brea, CA, USA). All sequencing data are available under controlled access through the DNA Databank of Japan (DDBJ; accession number DRA006473).

The 5′ and 3′ adapters were stripped from the reads by using Cutadapt (v1.7.1, https://cutadapt.readthedocs.io/en/stable/#) with the following parameters: -g TCGTCGGCAGCGTCAGATGTGTATAAGAGACAG -a CTGTCTCTTATACACATCTGACGCTGCCGACGATT -g GTCTCGTGGGCTCGGAGATGTGTATAAGAGACAG -a CTGTCTCTTATACACATCTCCGAGCCCACGAGACTT -O 15. The trimmed paired-end reads were mapped with Bowtie (v2) onto the *A. oryzae* genome sequences (A_oryzae_RIB40_current_chromosomes_13_Mar_2016 [25]) with default settings. Peaks were called using MACS (v1.4.2) with default settings, except for the following options: -f BAM -g 40,000,000. Once the peaks were ranked on the basis of fold enrichment, the peak interval data were converted to that of 50-bp sequences, which were cut out in each direction from the summit position by using BEDTools (v2.17.0) with the following parameters: bedtools slop -l 24 -r 24. The sequence data were extracted using the fastaFromBed utility in BEDTools.

Candidate promoters regulated by AoXlnR were annotated as follows: The *A. oryzae* upstream1000 dataset, which contains the 1000-bp region upstream of all of the predicted *A. oryzae* genes, was obtained using A_oryzaeRIB40_current_orf_genomic_1000.fasta [25]. The 50-bp sequences obtained from the third round of selection were annotated by local BLAST in *A. oryzae* upstream1000 with the following parameters: blastn -evalue 10 -outfmt 6. Subsequently, motifs were identified by MEME (v 4.10.2) with the following parameters: -dna -maxsize 500,000 -nmotifs 5 -revcomp -maxw 20.

### Affinity determination for MBP-AoXlnR against AoXlnR-binding fragments by using biolayer interferometry (BLI)

The XRE-WT fragment (the region between − 179 to − 93 bp from the initiation codon of AO090103000423 [*xynF1*], an AoXlnR-binding fragment retaining two AoXlnR-binding motifs reported in the previous study [21]), was amplified from *A. oryzae* genomic DNA with 0.25 μM of each of the primers XRE-S-bio and XRE-AS (Additional file 1: Table S1). xynF1_upstream _1 (the region between − 470 to − 380 bp from the initiation codon of *xynF1*) was amplified from *A. oryzae* genomic DNA with 0.25 μM each of xynF1-F-bio and xynF1-R (Additional file 2: Figure S1A and Additional file 1: Table S1). Double-stranded egl-242 (the region between − 781 to − 727 bp from the initiation codon of AO090023000787), egl-363 (the region between − 660 to − 607 bp from the initiation codon of AO090023000787), egl-617 (the region between − 406 to − 356 bp from the initiation codon of AO090023000787), abf-687 (the region between − 344 to − 295 bp from the initiation codon of AO090701000885), or abf-830 (the region between − 187 to − 139 bp from the initiation codon of AO090701000885) was prepared with bio-polyA as a complementary oligonucleotide by using Klenow fragments (Takara; Additional file 2: Figure S1B, C; Additional file 1: Table S1). All the biotinylated fragments were purified using MinElute PCR Purification Kit for BLI analysis.

The BLI is an optical analytical technique for monitoring biomolecular interactions utilizing the interference pattern of white light as it is reflected from a layer of immobilized biomolecules versus an internal reference layer [26]. The affinities of MBP-AoXlnR against several AoXlnR-binding fragments in the AO090103000423 (*xynF1*), AO090023000787, or AO090701000885 promoter region were determined by using the BLI on a BLItz system equipped with streptavidin sensors (Pall-ForteBio, Menlo Park, CA). A baseline was initially established in Kinetics Buffer 10× (Pall-ForteBio; 30 s). Then, 4 μL of each biotinylated ligand (500 nM in Kinetics Buffer 10×) was captured (2 min) and a baseline was re-established in Kinetics Buffer 10× (30 s). To analyze the affinity between MBP-AoXlnR and the immobilized ligand, 4 μL of the given MBP-XlnR_1–183_ was captured (2 min), and the bound analyte was dissociated in 200 μL of Kinetics Buffer 10× (3 min). AoXlnR was diluted with Kinetics Buffer 10× to the desired concentration (0, 25, 50, or 100 nM for AoXlnR-binding fragments in the *xynF1*; 0, 100, 200, or 500 nM for those in AO090023000787, or AO090701000885). All measurements were performed at room temperature with agitation. Data analysis and fitting were performed by using global fitting mode in a 1:1 binding model.

### Statistical analysis of AoXlnR-binding sequence

Correlations between HXlnR/ΔXlnR ratio for each expression level (i.e., differential gene expression between AoXlnR-overproducing and AoXlnR-disrupted *A. oryzae* strains [22]) and several parameters, which possibly affect the binding of AoXlnR, were analyzed using Spearman’s rank correlation test and Pearson’s correlation test in R software. Here, the parameters examined were as follows; the AoXlnR-binding motif numbers in the promoters of the DEGs, the fold enrichment values of and the summit position of the detected peaks from the selection round 3 in gSELEX (Additional file 3: Table S2).

## Results

### In vitro selection of AoXlnR-binding DNAs using gSELEX-Seq for identification of the candidate promoters regulated by AoXlnR

First, for performing gSELEX-Seq, the *A. oryzae* genomic DNA was fragmented to approximately less than 100 bp, ligated with linkers at both ends, and amplified using PCR (Additional file 4: Figure S2). Next, the DNA fragments binding to MBP-XlnR_1–183_, the MBP-fused AoXlnR DNA-binding domain, were selected from genomic library by three rounds of gSELEX. The DNA pools selected in each round of gSELEX were amplified with biotin and Cy5-labeled primers and then immobilized onto streptavidin-coated beads. Each bead pool was incubated with MBP-XlnR_1–183_ followed by immunostaining with a fluorescein-labeled anti-MBP antibody. The bead complexes were analyzed by flow cytometry to monitor the progress of the gSELEX selection process (Additional file 5: Figure S3A). The relative fluorescein intensity increased with each round of selection. This result suggests that the DNA pool binding to AoXlnR was highly enriched in each round from the *A. oryzae* genomic library in gSELEX. Even in selection round 3, an increase in the relative binding activity was observed (Additional file 5: Figure S3B). In our previous approach using AmyR from *A. nidulans* (AnAmyR), saturation of the enrichment of the bound fragments was observed in selection round 3 of gSELEX analysis [12]. This increased enrichment efficiency might be caused by more appropriate selection conditions than previous one. It should be noted here that we used purified MBP-AoXlnR_1–183_ and not crude protein solution.

All the DNA pools selected from the genomic library were sequenced using an Illumina MiSeq system, followed by bioinformatics analysis for genome-wide identification of AoXlnR-binding sites. After the mapping of the sequenced tags onto the *A. oryzae* genome and the detection of peaks with high numbers of the mapped tags, 50-bp DNA fragment around the top of the peaks were cut out.

Each 50-bp tag from round 3 was annotated by local BLAST with the *A. oryzae* upstream1000 dataset. Based on the annotation, we successfully identified 1948 promoters, including those of eight genes under the control of AoXlnR (*xynF1*, *xynG1*, *xynG2*, *xylA*, *celA*, *celB*, *celC*, and *celD*) [22] (Additional file 6: Table S3). This result reinforces the robustness of the genome-wide identification system for TF-regulated promoters using gSELEX-Seq.

### Identification of the genes regulated directly by AoXlnR

**Fig. 1:**
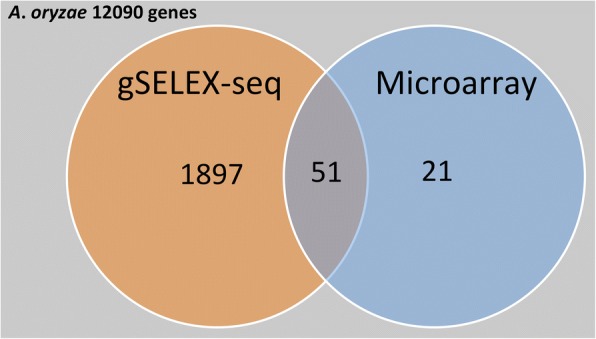
Venn diagram of the numbers of XlnR-related genes from gSELEX and microarray analyses. gSELEX-Seq: genes under the control of candidate AoXlnR-regulated promoters, obtained using gSELEX; Microarray: DEGs that showed more than five-fold higher expression in the AoXlnR overproducer than in the disruptant, which were identified using microarray analysis [22]. Values indicate the total number of genes in each set

In order to identify AoXlnR targets, Noguchi et al. carried out DNA microarray analysis in which the data based on spots of the scanned microarray images were analyzed for measure the expression level in each gene according to the procedure reported by Tamano et al. [22, 27]. By the DNA microarray analysis, 75 DEGs that showed more than five-fold greater expression in the AoXlnR overproducer than in the disruptant were identified [22]. From these 75 genes, AO090003001341, AO090701000828, and AO090005000768 were removed from the gene ID list in AspGD based on the current data of gene information for *A. oryzae*. The remaining 72 DEGs were compared with the 1948 genes possibly regulated by AoXlnR obtained from gSELEX-Seq; consequently, an intersecting subset had 51 genes (Fig. 1, Additional files 3 and 6: Table S2 and S3). This subset includes not only the aforementioned eight genes regulated directly by AoXlnR but also xylanolytic genes (AO090001000208, AO090005000698, and AO090011000140) and glycolytic genes (AO090701000274, AO090103000087, and AO090012000445). Remarkably, the percentage of promoters containing the canonical binding motifs (5′-GGCTA/GA-3′) in the 51 DEGs (86.3% [44/51]), was significantly higher than that in the other 21 DEGs (57.1% [12/21]; *p* < 0.05 by Fisher’s Exact Test). Generally, the genes identified as DEGs in microarray analysis should include ones regulated primarily or secondarily by the TFs. Together, the expressions of the 51 DEGs downstream of the promoters are considered to be directly controlled by AoXlnR. On the other hand, the other 21 DEGs, which were not included among the genes downstream of the candidate promoters, are considered to be indirectly regulated by AoXlnR.

### de novo motif analysis for identification of AoXlnR-binding sites

**Fig. 2:**
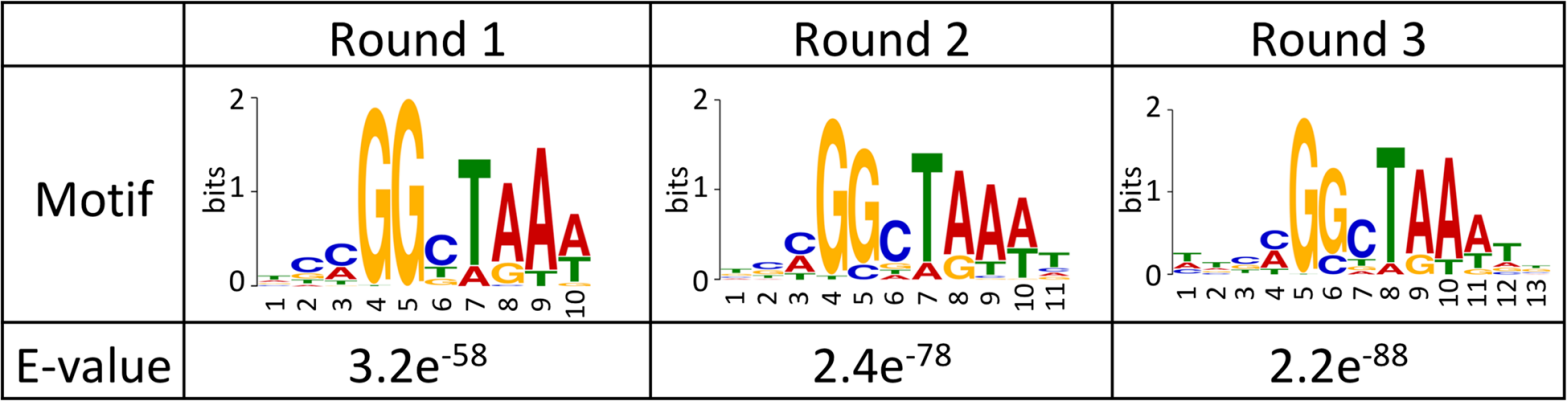
Analysis of the AoXlnR-binding motif. From the sequence of the peaks, 50-bp tags were extracted, and de novo motif analysis of the AoXlnR-binding site with the top 100 tags ranked according to fold enrichment was performed using MEME (v 4. 10.2). Here, only motifs that showed an E-value of < 1 are shown

de novo motif analysis of AoXlnR-binding sites was performed using the extracted 50-bp tags from gSELEX-Seq analysis (Fig. 2). The results revealed that 5′-(c/a) GGcT (A/g)(A/t)(a/t)-3′ was clearly detected as an AoXlnR-binding motif in all selection rounds. The binding motif contained one of the major canonical XlnR-binding motif, 5´-GGCTAA-3´ [21]. It should be noted that guanine was also detected at a relatively high frequency at the fifth base in the motif. This result is quite reasonable because AoXlnR binds the 5´-GGCTGA-3´ sequence with lower binding affinity than 5´-GGCTAA-3′ [20].

### Integrated data mining with differential expression levels obtained from microarray analysis

**Fig. 3:**
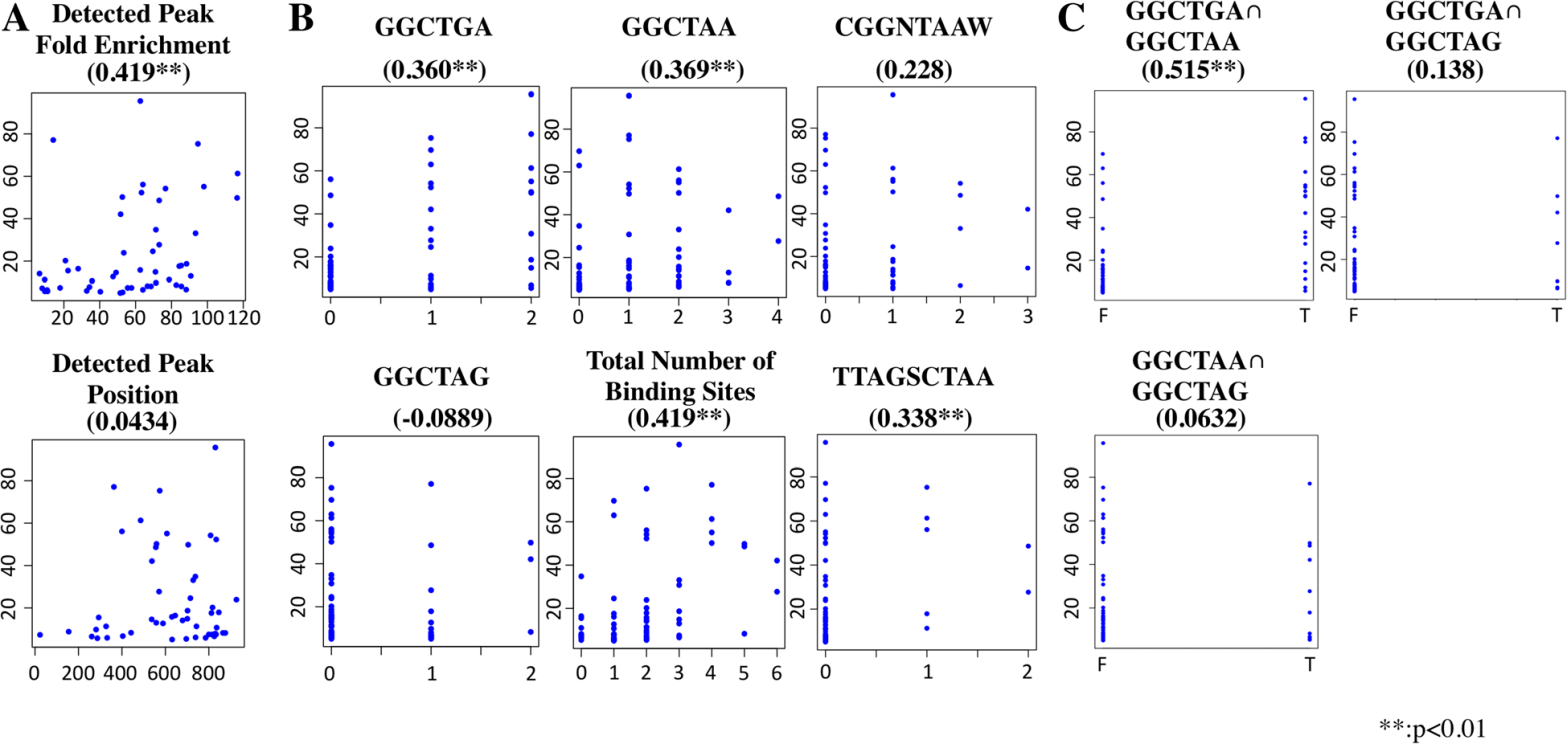
Scatter plots showing correlation between various factors related to AoXlnR binding and differential expression levels. (**a**) Correlations between parameters obtained from gSELEX-Seq analysis and the differential expression levels in the 51 genes in the intersecting set in Fig. 1. Detected Peak Fold Enrichment, fold enrichment detected in the promoter regions of the 51 genes by gSELEX-Seq analysis; Detected Peak Position, the summit of the peaks detected in the promoter regions of the 51 genes by gSELEX-Seq analysis. Here, the position of the base just before the start codon is set to 1000. (**b**) Correlations between the numbers of AoXlnR-binding sites and the differential expression levels in the 72 DEGs [22]. GGCTGA, the number of 5′-GGCTGA-3′ sites; GGCTAA, the number of 5′-GGCTAA-3′ sites; GGCTAG, the number of 5′-GGCTAG-3′ sites; Total Number of Binding Sites, the total number of 5′-GGCTAG-3′, 5′-GGCTAA-3′ and 5′-GGCTGA-3′ sites; CGGNTAAW, the number of 5′- CGGNTAAW-3′ sites; TTAGSCTAA, the number of 5′-TTAGSCTAA-3′ sites. (**c**) Correlations between the coexistence of two kinds of canonical AoXlnR-binding motifs and the differential expression levels of the 72 DEGs. GGCTGA∩GGCTAA, the set intersection of promoter regions containing 5′-GGCTGA-3′ and 5′-GGCTAA-3′; GGCTAA∩GGCTAG, the set intersection of promoter regions containing 5′-GGCTAA-3′ and 5′-GGCTAG-3′; GGCTGA∩GGCTAG, the set intersection of promoter regions containing 5′-GGCTGA-3′ and 5′-GGCTAG-3′. The promoter regions containing both of the two kinds of motifs are categorized in T wheres the others are in F. The vertical axis in each scattergram shows the differential expression levels. Values in parentheses show the Spearman’s rank correlation coefficient with the differential expression levels

To further investigate the transcriptional mechanisms regulated by AoXlnR, correlations between various parameters related to AoXlnR binding and the differential expression levels were analyzed (Fig. 3 and Additional files 3 and 7: Table S2 and S4). Although the peak positions detected were irrelevant for the differential expression levels of the 51 genes, the fold enrichment values, which were detected from gSELEX-Seq analysis, showed significant correlation for the differential expression level of each gene (Spearman’s rank correlation coefficient = 0.419, *p* < 0.01; Fig. 3a and Additional file 7: Table S4). This result indicates that the binding preference of AoXlnR identified by gSELEX-Seq correlated with the phenotype of AoXlnR analyzed by microarray. In addition, there was a significant correlation between the total number of AoXlnR-binding motifs (i.e., sum of the 5′-GGCTAG-3′, 5′-GGCTAA-3′, and 5′-GGCTGA-3′ sites on each promoter) and the differential expression level of each gene (Spearman’s rank correlation coefficient = 0.419, *p* < 0.01; Fig. 3b and Additional file 7: Table S4). Interestingly, high correlations were showed between the differential expression level and the number of 5′-GGCTGA-3′ (Spearman’s rank correlation coefficient = 0.360; *p* < 0.01) as well as that of the major canonical binding motif, 5′-GGCTAA-3′ sites (Spearman’s rank correlation coefficient = 0.369; *p* < 0.01) (Fig. 3b and Additional file 7: Table S4). The number of 5′-GGCTGA-3′sites also showed a considerably high correlation with the differential expression level in Pearson’s correlation test (Pearson’s correlation coefficient = 0.519; *p* < 0.01; Additional file 7: Table S4). These results suggest that the 5′-GGCTGA-3′ site present upstream of the gene can greatly contribute to the activation by AoXlnR in *A. oryzae* cells.

## Discussion

AoXlnR, a TF of *A*. *oryzae*, is a key player in cellulose and xylan metabolisms, and activates the response of xylanolytic and cellulolytic genes [20–22]. In this study, the gene expression network controlled by AoXlnR was investigated using integrated mining of gSELEX-Seq and microarray data.

By using gSELEX-Seq, 1948 promoters were identified as the candidate promoters regulated by AoXlnR (Additional file 6: Table S3). Although the canonical AoXlnR-binding motifs, 5′-GGCTGA-3′ and 5′-GGCTAG-3′ were present near the detected summits of the peaks in the promoters of *xlnA* and *celA*, respectively, there were no binding motifs near those upstream of *xynF1*, *xynG1*, *xynG2*, *celB*, *celC*, and *celD* (Additional file 8: Figure S4, Additional file 3: Table S2). On the other hand, the regions flanking the detected six summits contained sequences similar to the consensus motifs. These results suggest that the binding specificity of AoXlnR is relatively low, and consequently, the fragments containing sequences similar to the core motif were enriched in the gSELEX process.

To confirm the hypothesis mentioned above, the binding affinity of AoXlnR for a fragment containing the detected summit of the peak in the promoter of *xynF1* was examined using BLI. The kinetic analysis indicated that xynF1_upstream_1 binds AoXlnR with higher affinity (apparent KD = 311 nM) than XRE-WT (apparent KD = 656 nM) (Table 1). Here, xynF1_upstream_1 is a 100-bp fragment including the summit of the peak detected by gSELEX-Seq (Additional file 2: Figure S1A). Note that the 5′-GGGTAA-3′ region, similar to an AoXlnR-binding motif, is present in xynF1_upstream_1 at two places (Additional file 8: Figure S4). On the other hand, XRE-WT is an AoXlnR-binding fragment retaining two AoXlnR-binding motifs (5′-GGCTAA-3′ and 5′-GGCTGA-3′) reported in the previous study [21] (Additional file 2: Figure S1A). It should be also noted that a minor peak near the AoXlnR-binding motif was detected in the XRE-WT region in the mapping data (Additional file 9: Figure S5). Together, these results, which AoXnlR binds with moderate affinity to a region containing no canonical binding motifs, indicated that the in vitro AoXlnR-binding specificity is relatively low. Furthermore, the results also strongly reinforce the idea that DNA fragments were preferentially enriched in accordance with their in vitro affinities for AoXlnR during the gSELEX process.

**Table 1:** Binding activity of MBP-AoXlnR_1–183_-bound fragments

Sample ID	KD (nM)	ka (1/Ms)	kd (1/s)
xynF1_upstream_1	311	7.14 × 10^4^	2.22 × 10^− 2^
XRE-WT	656	5.99 × 10^4^	3.93 × 10^− 2^
egl-242	40.6	1.24 × 10^5^	5.04 × 10^− 3^
egl-363	68.7	1.23 × 10^5^	8.44 × 10^− 3^
egl-617	48.4	9.06 × 10^4^	4.39 × 10^− 3^
abf-687	133	9.85 × 10^4^	1.31 × 10^− 2^
abf-830	144	1.54 × 10^5^	2.22 × 10^− 2^

As described above, using gSELEX-Seq, 1948 promoters were identified as the candidate promoters regulated by AoXlnR (Additional file 6: Table S3). However, only 51 genes among them were included in the intersecting subset of genes identified by the gSELEX-Seq and the microarray analysis (Fig. 1). Since it is highly unlikely that AoXlnR regulates all of these genes in *A. oryzae* cells, we speculate that most of the other 1897 genes are false positives in gSELEX analysis. However, it is possible that the subset of the 1897 genes might include unidentified DEGs by the microarray analysis, which was conducted with mRNA extracted from mycelia that had been exposed to D-xylose for 30 min [22]. The unidentified DEGs might be revealed by RNA-Seq analysis with another AoXlnR induction condition.

In de novo motif analysis using the extracted 50-bp tags from the gSELEX-Seq, 5′-(c/a)GGcT(A/g)(A/t)(a/t)-3′ was clearly detected as an AoXlnR-binding motif (Fig. 2). Recent studies have shown that the AoXlnR monomer binds to 5′-CGGNTAA (A/T)-3′ and the dimer to 5′-TTAG(G/C) CTAA-3′, respectively [28, 29]. The motif detected in Fig. 2 contained only the AoXlnR monomer-binding motif, suggesting that the recombinant AoXlnR prepared in this study, MBP-XlnR_1–183_, was predominantly present as a monomer. Therefore, it may be difficult to analyze the binding sites of AoXlnR dimer using the recombinant MBP-XlnR_1–183_. However, the association efficiency of the AoXlnR monomer might be enhanced by the attaching a protein tag, which accelerates the homodimerization of the expressed protein, to the end of the AoXlnR. We are currently planning to conduct gSELEX-Seq using AoXlnR fused with GST, which is a homodimer forming protein [30].

The results of integrated data mining with differential expression levels obtained from microarray analysis suggested that the 5′-GGCTGA-3′ sites in the promoters are closely related to the regulation by AoXlnR (Fig. 3b). The promoter regions of AO090023000787 and AO090701000885, which showed significant differential expression, contain two 5′-GGCTGA-3′ sites. Therefore, 50-bp regions containing the 5′-GGCTGA-3′ in the two promoters were applied to BLI analysis for characterization of the AoXlnR-binding preference (Table 1 and Additional file 2: Figure S1). Interestingly, all fragments containing the binding motif analyzed were bound to AoXlnR with nanomolar affinities. The results strongly suggested that all the 5′-GGCTGA-3′ sites in the promoters subject to regulation by AoXlnR.

On the other hand, using gel shift assays, Marui et al. showed that AoXlnR preferentially binds the 5′-GGCTAA-3′ region in the *xynF1* promoter with approximately 10-times higher affinity than that for 5′-GGCTGA-3′ in the promoter [20]. In addition, they showed that the 5′-GGCTAA-3′ in the *xynF1* promoter is utilized in vivo for induction by xylan and d-xylose more efficiently than the 5′-GGCTGA-3′ site [19, 20]. The results of these previous studies are inconsistent with our findings that the *cis*-element 5′-GGCTGA-3′ is significantly involved in the expression regulation by AoXlnR, comparable to 5′-GGCTAA-3′ (Fig. 3b).

Of the 29 promoters possessing one or more 5′-GGCTGA-3′ sites among the 72 DEGs identified by microarray analysis [22], most of them (21 promoters) also possessed 5′-GGCTAA-3′ or 5′-GGCTAG-3′ sites. Therefore, we analyzed the correlations between the coexistence of the two kinds of canonical AoXlnR-binding motifs and differential expression (Fig. 3c and Additional file 7: Table S4). The analysis showed that the presence of both the 5′-GGCTGA-3′ and 5′-GGCTAA-3′ sites was significantly correlated with differential expression levels (Spearman’s rank correlation coefficient = 0.515, *p* < 0.01). By contrast, there was no significant correlation in the case of 5′-GGCTAA-3′ and 5′-GGCTAG-3′ (Spearman’s rank correlation coefficient = 0.0632) or 5′-GGCTGA-3′ and 5′-GGCTAG-3′ (Spearman’s rank correlation coefficient = 0.138). The data mining strongly suggests that the coexistence of 5′-GGCTGA-3′ and 5′-GGCTAA-3′ sites in the promoter enhances the expression of genes located downstream of the sites. In fact, the xylan-dependent expression level of a reporter gene decreased moderately by introducing of a point mutation into the 5′-GGCTGA-3′ site [20]. Together, these results suggest that the 5’-GGCTGA-3′ motif might be a *cis*-element that further enhances the transcriptional activation by AoXlnR bound preferentially onto the 5′-GGCTAA-3′ site.

The approach adopted in this study can be used for the analysis of AoXlnR-mediated cellulose and xylan metabolic network. In addition, the information of the correlation between the presence of the AoXlnR motifs and the expression of genes located downstream of the sites can be applied to *A. oryzae* metabolic engineering approaches utilizing cellulose as a starting material. Several synthetic biological approaches with a genetically engineered *A. oryzae* for cellulosic fermentation have been reported [31, 32]. In these approaches, however, several kinds of foreign cellulase genes were introduced into an *A. oryzae* transformant to enhance the cellulase activity. Considering the hypothesis described above, AoXlnR can further activate the expression of a target intrinsic cellulose gene by newly introducing the 5′-GGCTGA-3′ motif into the promoter of the gene. The motif can be inserted at a desired site onto the *A. oryzae* genome by using the CRISPR/Cas9 system of filamentous fungi [33–35] to increase the cellulase activity of the strain. In principle, this strategy can be applied to various metabolite fermentations using *A. oryzae* as a cell factory. Hence, if our pipeline is combined with the CRISPR/Cas9 system, it may be possible to rationally design a metabolic process in *A. oryzae*.

Recently, Wang et al. performed a genome-wide analysis of TF-binding sites in *A. oryzae* by using DNase I digestion coupled with high-throughput sequencing (DNase-Seq), consequently identifying the binding site of 19 known TFs based on the digestion profile [36]. In addition, the authors found that the DNase I cleavage patterns of TFs were consistent with DNA shape features, such as minor groove twist (MGW), propeller twist (ProT), helix twist (HelT), and Roll. In the report, however, the target TF phenotypes were not considered. On the other hand, our pipeline, which integrates both data of in vitro TF-binding characteristics and DEGs, can output not only the genes regulated directly by a TF but also the TF-binding *cis*-elements related closely to the expression of the target genes. A further robust transcriptome analysis system may be established by incorporating the DNA shape features described above as parameters into our pipeline.

## Conclusions

In this study, the genes regulated directly by AoXlnR were successfully identified by integrated mining of data obtained from gSELEX-Seq and microarray. The data mining of the promoters of the DEGs showed that the presence of both the canonical AoXlnR-binding motifs, 5′-GGCTAA-3′ and 5′-GGCTGA-3′, are related significantly to the expression of genes located downstream of the sites. These findings will contribute greatly to the elucidation of AoXlnR-mediated cellulose and xylan metabolic network in *A. oryzae*. In addition, the pipeline, which is based on integrated mining of data consisting of both in vitro characterization of the DNA-binding sites and TF phenotype, can be a robust platform for comprehensive analysis of gene expression network via TFs.

## Additional files

Additional file 1: **Table S1.** Oligonucleotide primers list. (DOCX 94 kb)

Additional file 2: **Figure S1.** Promoter region used for affinity analysis. (A) The regions of XRE-WT and xynF1_upsteram_1 in xynF1Blue and red sequences indicate the regions of xynF1_upstream_1 and XRE-WT, respectively. Lower cases indicate mutation sites to derive from the designed primer. (B) The regions of egl-242, egl-363 and egl-617 are derived from promoter of AO090023000787. Blue, red and green sequences indicate the regions of egl-242, egl-363 and egl-617, respectively. (C) The regions of abf-680 and abf-837 are derived from promoter of AO090701000885. Blue and red sequences indicate the regions of of abf-680 and abf-837, respectively. AoXlnR binding motifs are shaded. Asterisks indicate the summit position of the detected peaks from the selection round 3 in gSELEX. Italic characters indicate possible AoXlnR binding sites. (DOCX 213 kb)

Additional file 3: **Table S2.** Parameter list in data mining of AoXlnR binding sequence. (N), the number of the site; (P), the posion of the site; (FE), fold enrichment. The Gene ID in gray column indicates that the peak was detected in the promoter in gSELEX-Seq analysis. (DOCX 609 kb)

Additional file 4: **Figure S2.** Construction of *A. oryzae* genomic library. (A) Genomic DNA shearing by using DNA-shearing system M220 (Life Technologies). (B) Gel extraction of size-fractionated genomic library after linker ligation. Bands of approximately 100–250 bp were excised using a spatula. (C) Genomic library after PCR amplification using the gel extraction product as a template. Red arrow indicates the amplicon. (TIFF 33973 kb)

Additional file 5: **Figure S3.** Flow cytometric analysis of selected DNA pools from gSELEX by using bead display. (A) Dot plot of fluorescence analysis. X-axis is quantified fluorescence intensity in the FL1 channel and Y-axis is quantified fluorescence intensity in FL5. (B) Relative binding affinities to XlnR was calculated by the dividing the intensity of FL1 by that of FL5 and the binding affinity of Round 0 against AoXlnR was set as 1. (TIFF 33973 kb)

Additional file 6: **Table S3.** *A. oryzae* promoter regions selected by gSELEX-Seq. (DOCX 239 kb)

Additional file 7: **Table S4.** Correlation analyses between various factors related to AoXlnR binding and differential expression levels. (DOC 49 kb)

Additional file 8: **Figure S4.** Summits of peaks detected using gSELEX-Seq in the promoter regions of *xynF1*, *xynG1*, *xynG2*, *xylA*, *celA*, *celB*, *celC* and *celD*. gSELEX-Seq peaks were detected using MACS (v1.4.2). Blue squares indicate the summits of the peaks. Red square frames indicate canonical XlnR binding motifs. (DOCX 278 kb)

Additional file 9: **Figure S5.** Mapping of selected DNAs in *xynF1* promoter region. Sequence tags from each round were mapped on *A. oryzae* mapped with Bowtie (v2). The mapping results were visualized by Integrative Genomics Viewer (IGV). Red arrow indicates the translation initiation site. (TIFF 33973 kb)

## Abbreviations

BLI: Biolayer interferometry
gSELEX-Seq: Genomic systematic evolution of ligands by exponential enrichment-Seq
MBP: Maltose-binding protein
TFs: Transcription factors

## References

[1] Todeschini AL, Georges A, Veitia RA (2014). Transcription factors: specific DNA binding and specific gene regulation. Trends genet.

[2] Johnston M. A model fungal gene regulatory mechanism: the GAL genes of *Saccharomyces cerevisiae*. Microbiol Rev. 1987;51:458–76. Available from: http://mmbr.asm.org/content/51/4/458.long. 10.1128/mr.51.4.458-476.1987 PMC373127 2830478

[3] Marmorstein R, Carey M, Ptashne M, Harrison SC (1992). DNA recognition by GAL4: structure of a protein-DNA complex. Nature.

[4] Schena M, Shalon D, Davis RW, Brown PO (1995). Quantitative monitoring of gene expression patterns with a complementary DNA microarray. Science.

[5] Zheng M, Wang X, Templeton LJ, Smulski DR, LaRossa RA, Storz G. DNA Microarray-Mediated Transcriptional Profiling of the *Escherichia coli* Response to Hydrogen Peroxide. J Bacteriol. 2001;183:4562–70. Available from: http://jb.asm.org/content/183/15/4562.long. 10.1128/JB.183.15.4562-4570.2001 PMC95351 11443091

[6] Mao J, Habib T, Shenwu M, Kang B, Allen W, Robertson L, et al. Transcriptome profiling of *Saccharomyces cerevisiae* mutants lacking C2H2 zinc finger proteins. BMC Genomics. 2008;9:1–9. Available from: https://bmcgenomics.biomedcentral.com/articles/10.1186/1471-2164-9-S1-S14. 10.1186/1471-2164-9-S1-S14 PMC2386056 18366603

[7] Oliphant AR, Brandl CJ, Struhl K (1989). Defining the sequence specificity of DNA-binding proteins by selecting binding sites from random-sequence oligonucleotides: analysis of yeast GCN4 protein. Mol Cell Biol [Internet].

[8] Tuerk C, Gold L (1990). Systematic evolution of ligands by exponential enrichment: RNA ligands to bacteriophage T4 DNA polymerase. Science.

[9] Ellington AD, Szostak JW (1990). In vitro selection of RNA molecules that bind specific ligands. Nature.

[10] Singer BS, Shtatland T, Brown D, Gold L (1997). Libraries for genomic SELEX. Nucleic Acids Res.

[11] Dror I, Golan T, Levy C, Rohs R, Mandel-Gutfreund Y (2015). A widespread role of the motif environment in transcription factor binding across diverse protein families. Genome Res.

[12] Kojima T, Kunitake E, Ihara K, Kobayashi T, Nakano H (2016). A robust analytical pipeline for genome-wide identification of the genes regulated by a transcription factor: Combinatorial analysis performed using gSELEX-Seq and RNA-Seq. PLoS One.

[13] Barbesgaard P, Heldt-Hansen HP, Diderichsen B. On the safety of *Aspergillus oryzae*: a review. Appl Microbiol Biotechnol. 1992;36:569–72. Available from: https://link.springer.com/article/10.1007%2FBF00183230. 10.1007/BF00183230 1368061

[14] Beguin P (1990). Molecular Biology of cellulose degradation. Microbiol.

[15] van Peij NN, Gielkens MM, de Vries RP, Visser J, de Graaff LH. The transcriptional activator XlnR regulates both Xylanolytic and endoglucanase gene expression in *Aspergillus niger*. Appl Environ Microbiol. 1998;64:3615–9. Available from: http://aem.asm.org/content/64/10/3615.long. 10.1128/aem.64.10.3615-3619.1998 PMC106473 9758775

[16] de Vries RP, Visser J, de Graaff LH. CreA modulates the XlnR-induced expression on xylose of *Aspergillus niger* genes involved in xylan degradation. Res Microbiol. 1999;150:281–5. Available from: https://www.sciencedirect.com/science/article/pii/S0923250899800539?via%3Dihub. 10.1016/s0923-2508(99)80053-9 10376490

[17] van Peij NN, Visser J, de Graaff LH. Isolation and analysis of xlnR, encoding a transcriptional activator coordinating xylanolytic expression in *Aspergillus niger*. Mol Microbiol. 1998;27:131–42. Available from: https://onlinelibrary.wiley.com/doi/abs/10.1046/j.1365-2958.1998.00666.x. 10.1046/j.1365-2958.1998.00666.x 9466262

[18] Gielkens MM, Dekkers E, Visser J, de Graaff LH. Two Cellobiohydrolase-encoding genes from *Aspergillus niger* require D-xylose and the Xylanolytic transcriptional activator XlnR for their expression. Appl Environ Microbiol. 1999;65:4340–5. Available from: http://aem.asm.org/content/65/10/4340.long. 10.1128/aem.65.10.4340-4345.1999 PMC91575 10508057

[19] de Vries RP, van de Vondervoort PJ, Hendriks L, van de Belt M, Visser J. Regulation of the α-glucuronidase-encoding gene ( aguA ) from *Aspergillus niger*. Mol Genet Genomics. 2002;268:96–102. Available from: https://link.springer.com/article/10.1007%2Fs00438-002-0729-7. 10.1007/s00438-002-0729-7 12242504

[20] Marui J, Kitamoto N, Kato M, Kobayashi T, Tsukagoshi N. Transcriptional activator, AoXlnR, mediates cellulose inductive expression of the xylanolytic and cellulytic genes in *Aspergillus oryzae*. FEBS Lett. 2002;528:279–82. Available from: https://febs.onlinelibrary.wiley.com/doi/abs/10.1016/S0014-5793%2802%2903328-8. 10.1016/s0014-5793(02)03328-8 12297320

[21] Marui J, Tanaka A, Mimura S, de Graaff LH, Visser J, Kitamoto N, et al. A transcriptional activator, AoXlnR, controls the expression of genes encoding xylanolytic enzymes in *Aspergillus oryzae*. Fungal Genet Biol. 2002;35:157–69. Available from: https://www.sciencedirect.com/science/article/pii/S1087184501913210?via%3Dihub. 10.1006/fgbi.2001.1321 11848678

[22] Noguchi Y, Sano M, Kanamaru K, Ko T, Takeuchi M, Kato M, et al. Genes regulated by AoXlnR, the xylanolytic and cellulolytic transcriptional regulator, in *Aspergillus oryzae*. Appl Microbiol Biotechnol. 2009;85:141–54. Available from: https://link.springer.com/article/10.1007%2Fs00253-009-2236-9. 10.1007/s00253-009-2236-9 19777228

[23] Kojima T, Hashimoto Y, Kato M, Kobayashi T, Nakano H. High-throughput screening of DNA binding sites for transcription factor AmyR from *Aspergillus nidulans* using DNA beads display system. J Biosci Bioeng. 2010;109:519–25. The Society for Biotechnology, Japan; Available from: 10.1016/j.jbiosc.2009.11.024. 20471587

[24] Bradford MM (1976). A rapid and sensitive method for the quantitation microgram quantities of protein utilizing the principle of protein-dye binding. Anal Biochem.

[25] Cerqueira GC, Arnaud MB, Inglis DO, Skrzypek MS, Binkley G, Simison M, et al. The *Aspergillus* Genome Database: multispecies curation and incorporation of RNA-Seq data to improve structural gene annotations. Nucleic Acids Res. 2014;42(database issue):D705–10.https://www.ncbi.nlm.nih.gov/pubmed/24194595. 10.1093/nar/gkt1029 PMC3965050 24194595

[26] Concepcion J, Witte K, Wartchow C, Choo S, Yao D, Persson H (2009). Label-free detection of biomolecular interactions using BioLayer interferometry for kinetic characterization. Comb Chem High Throughput Screen.

[27] Tamano K, Sano M, Yamane N, Terabayashi Y, Toda T, Sunagawa M, et al. Transcriptional regulation of genes on the non-syntenic blocks of *Aspergillus oryzae* and its functional relationship to solid-state cultivation. Fungal Genet Biol. 2008;45:139–51. Available from: https://www.ncbi.nlm.nih.gov/pubmed/17967552. 10.1016/j.fgb.2007.09.005 17967552

[28] Kunitake E, Kobayashi T. Conservation and diversity of the regulators of cellulolytic enzyme genes in Ascomycete fungi. Curr genet. 2017;63:951–8. Berlin: Springer; Available from: https://link.springer.com/article/10.1007%2Fs00294-017-0695-6. 10.1007/s00294-017-0695-6 28451846

[29] Ishikawa K, Kunitake E, Kawase T, Atsumi M, Noguchi Y, Ishikawa S, et al. Comparison of the paralogous transcription factors AraR and XlnR in *Aspergillus oryzae*. Curr genet. 2018;64:1245–60. Berlin: Springer; Available from: 10.1007/s00294-018-0837-5. 29654355

[30] Nishida M, Harada S, Noguchi S, Satow Y, Inoue H, Takahashi K (1998). Three-dimensional structure of Escherichia coli glutathione S-transferase complexed with glutathione sulfonate: catalytic roles of Cys10 and His106. J Mol biol.

[31] Lin H, Wang Q, Shen Q, Ma J, Fu J, Zhao Y. Engineering *Aspergillus oryzae* A-4 through the chromosomal insertion of foreign cellulase expression cassette to improve conversion of cellulosic biomass into lipids. PLoS One. 2014;9:e108442. Available from: http://journals.plos.org/plosone/article?id=10.1371/journal.pone.0108442. 10.1371/journal.pone.0108442 PMC4177402 25251435

[32] Yamada R, Yoshie T, Wakai S, Asai-Nakashima N, Okazaki F, Ogino C, et al. *Aspergillus oryzae*-based cell factory for direct kojic acid production from cellulose, Microb Cell Fact. 2014;13:71. Available from: https://microbialcellfactories.biomedcentral.com/articles/10.1186/1475-2859-13-71. 10.1186/1475-2859-13-71 PMC4035902 24885968

[33] Nødvig CS, Nielsen JB, Kogle ME, Mortensen UH (2015). A CRISPR-Cas9 system for genetic engineering of filamentous fungi. PLoS One.

[34] Katayama T, Tanaka Y, Okabe T, Nakamura H, Fujii W, Kitamoto K, et al. Development of a genome editing technique using the CRISPR/Cas9 system in the industrial filamentous fungus *Aspergillus oryzae*. Biotechnol Lett. 2016;38:637–42. Available from: https://link.springer.com/article/10.1007%2Fs10529-015-2015-x. 10.1007/s10529-015-2015-x 26687199

[35] Nakamura H, Katayama T, Okabe T, Iwashita K, Fujii W, Kitamoto K, et al. Highly efficient gene targeting in *Aspergillus oryzae* industrial strains under ligD mutation introduced by genome editing: Strain-specific differences in the effects of deleting EcdR, the negative regulator of sclerotia formation. J Gen Appl Microbiol. 2017;63:172–8. Available from: https://www.jstage.jst.go.jp/article/jgam/63/3/63_2016.10.002/_article. 10.2323/jgam.2016.10.002 28484116

[36] Wang C, Lv Y, Wang B, Yin C, Lin Y, Pan L. Survey of protein-DNA interactions in *Aspergillus oryzae* on agenomic scale. Nucleic Acids Res. 2015;43:4429–46. Available from: https://academic.oup.com/nar/article/43/9/4429/1128438. 10.1093/nar/gkv334 PMC4482085 25883143

