# Supplementary material for: Comprehensive investigation of the gene expression system regulated by an Aspergillus oryzae transcription factor XlnR using integrated mining of gSELEX-Seq and microarray data
Source: BMC Genomics. 2019 Jan 8;20:16. doi: 10.1186/s12864-018-5375-5 (PMC6323846; doi:10.1186/s12864-018-5375-5)
Supplement: Supplementary file 1 — Table S1. Oligonucleotide primers list. (DOCX 94 kb) [file 12864_2018_5375_MOESM1_ESM.docx]

**Supplementary Table 1. Oligonucleotide primers list.**

| **Primer name** | **Primer sequence** | **Others** |
| --- | --- | --- |
| Nextra-Read1 | 5′-TCGTCGGCAGCGTCAGATGTGTATAAGAGACAG-3′ | For sequencing adaptor with an Illumina MiSeq sequencer (Illumina). |
| Nextra-Read2 | 5′-GTCTCGTGGGCTCGGAGATGTGTATAAGAGACAG-3′ | For sequencing adaptor with an Illumina MiSeq sequencer (Illumina). |
| Nextra-Read1-adp-comp | 5′-CTGTCTCTTATACACATCTGACGCTGCCGACGATT-3′ | For sequencing adaptor with an Illumina MiSeq sequencer (Illumina). |
| Nextra-Read2-adp-comp | 5′-CTGTCTCTTATACACATCTCCGAGCCCACGAGACTT-3′ | For sequencing adaptor with an Illumina MiSeq sequencer (Illumina). |
| XRE-AS | 5′-GCTCTAGATTCCCATTTGACCTGCC-3′ |  |
| XRE-S-bio | 5′-Biotin-GGAATTCGGGGTATTAGGCTAAACGTG-3′ |  |
| xynF1-R | 5′-AGCAGATGCTGAAACCCTGAGGA-3′ |  |
| xynF1-F-bio | 5′-Biotin-CCAGCCAACCTTCAGCTCAGCAA-3′ |  |
| egl-242 | 5′-GGAGACTCCTCTCATCTTGCTGGGCTGAAGACTCGGTCAATACCATGATACCTTTTTTTTTTTTTTTTTT-3′ |  |
| egl-363 | 5′-CAACCCGAGGGTCGATCGACTCCGGCATTAGCTGCCCAGCAGGTATACAGCCTTTTTTTTTTTTTTTTTT-3′ |  |
| egl-617 | 5′-GCAGCGGCGAAAGGTGCAGGATGGCTGACGCTCAGTTTAATGAGCAGTGGCCTTTTTTTTTTTTTTTTTT-3′ |  |
| abf-680 | 5′-GCAGAAGCTCTTGCAGATCTGTCGGCTGAAGGTTTGTTTTAGGCCAAATGCCTTTTTTTTTTTTTTTTTT-3′ |  |
| abf-837 | 5′-TTAAACACATGAGACACACTCGCGGCTGAATGGTTTCGGGTTTTAGCCGGCCTTTTTTTTTTTTTTTTTT-3′ |  |
| bio-polyA | 5′-Biotin-AAAAAAAAAAAAAAAAAAGG-3′ |  |
