# Supplementary material for: Comprehensive investigation of the gene expression system regulated by an Aspergillus oryzae transcription factor XlnR using integrated mining of gSELEX-Seq and microarray data
Source: BMC Genomics. 2019 Jan 8;20:16. doi: 10.1186/s12864-018-5375-5 (PMC6323846; doi:10.1186/s12864-018-5375-5)
Supplement: Supplementary file 2 — Figure S1. Promoter region used for affinity analysis. (A) The regions of XRE-WT and xynF1_upsteram_1 in xynF1Blue and red sequences indicate the regions of xynF1_upstream_1 and XRE-WT, respectively. Lower cases indicate mutation sites to derive from the designed primer. (B) The regions of egl-242, egl-363 and egl-617 are derived from promoter of AO090023000787. Blue, red and green sequences indicate the regions of egl-242, egl-363 and egl-617, respectively. (C) The regions of abf-680 and abf-837 are derived from promoter of AO090701000885. Blue and red sequences indicate the regions of of abf-680 and abf-837, respectively. AoXlnR binding motifs are shaded. Asterisks indicate the summit position of the detected peaks from the selection round 3 in gSELEX. Italic characters indicate possible AoXlnR binding sites. (DOCX 213 kb) [file 12864_2018_5375_MOESM2_ESM.docx]

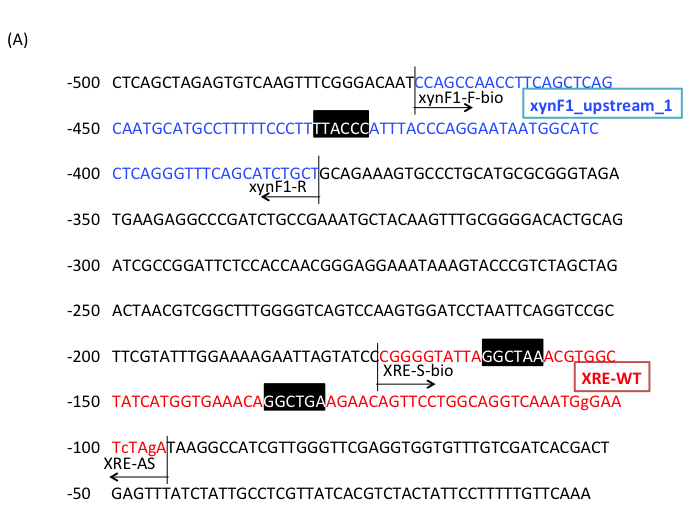


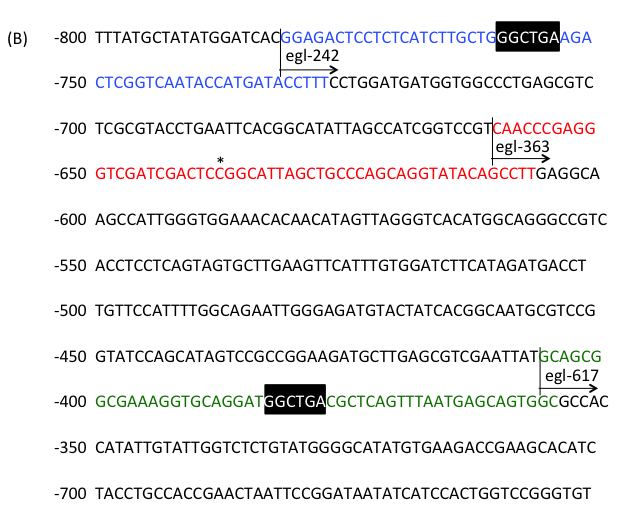


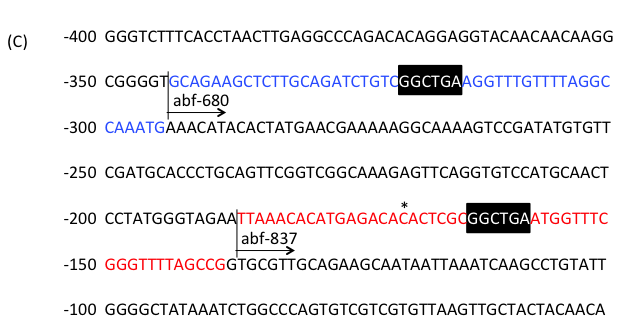


Supplementary Fig. 1. Promoter region used for affinity analysis.

(A) The regions of XRE-WT and xynF1_upsteram_1 in *xynF1*Blue and red sequences indicate the regions of xynF1_upstream_1 and XRE-WT, respectively. Lower cases indicate mutation sites to derive from the designed primer. (B) The regions of egl-242, egl-363 and egl-617 are derived from promoter of AO090023000787. Blue, red and green sequences indicate the regions of egl-242, egl-363 and egl-617, respectively. (C) The regions of abf-680 and abf-837 are derived from promoter of AO090701000885. Blue and red sequences indicate the regions of of abf-680 and abf-837, respectively. AoXlnR binding motifs are shaded. Asterisks indicate the summit position of the detected peaks from the selection round 3 in gSELEX. Italic characters indicate possible AoXlnR binding sites.
