# Supplementary material for: Comprehensive investigation of the gene expression system regulated by an Aspergillus oryzae transcription factor XlnR using integrated mining of gSELEX-Seq and microarray data
Source: BMC Genomics. 2019 Jan 8;20:16. doi: 10.1186/s12864-018-5375-5 (PMC6323846; doi:10.1186/s12864-018-5375-5)
Supplement: Supplementary file 3 — Table S2. Parameter list in data mining of AoXlnR binding sequence. (N), the number of the site; (P), the posion of the site; (FE), fold enrichment. The Gene ID in gray column indicates that the peak was detected in the promoter in gSELEX-Seq analysis. (DOCX 609 kb) [file 12864_2018_5375_MOESM3_ESM.docx]

**Supplementary Table 2. Parameter list in data mining of AoXlnR binding sequence.**

(N), the number of the site; (P), the posion of the site; (FE), fold enrichment. The Gene ID in gray column indicates that the peak was detected in the promoter in gSELEX-Seq analysis.
