# Supplementary figures and images for: Comprehensive investigation of the gene expression system regulated by an Aspergillus oryzae transcription factor XlnR using integrated mining of gSELEX-Seq and microarray data
Source: BMC Genomics. 2019 Jan 8;20:16. doi: 10.1186/s12864-018-5375-5 (PMC6323846; doi:10.1186/s12864-018-5375-5)

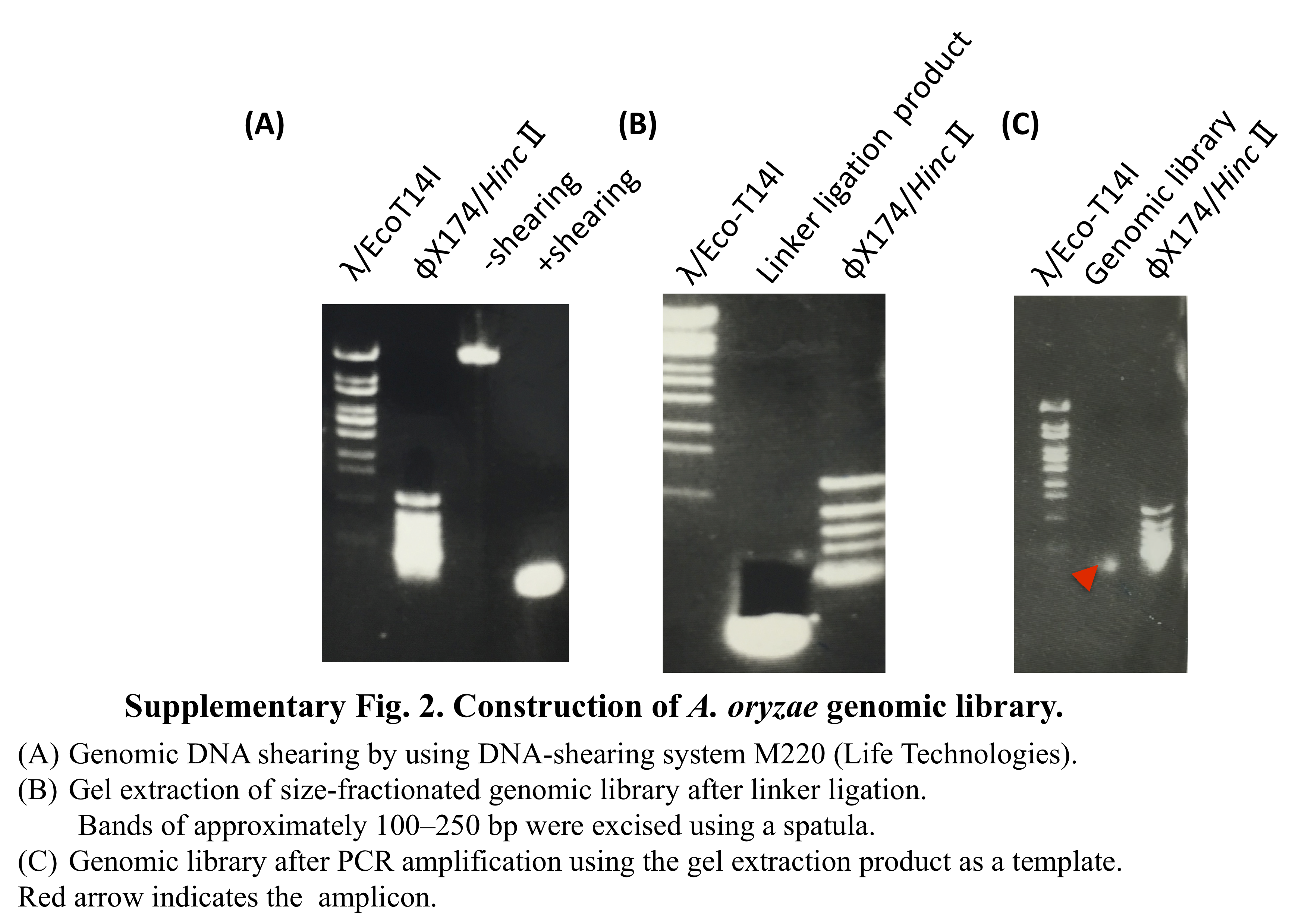

Supplement: Supplementary file 4 — Figure S2. Construction of A. oryzae genomic library. (A) Genomic DNA shearing by using DNA-shearing system M220 (Life Technologies). (B) Gel extraction of size-fractionated genomic library after linker ligation. Bands of approximately 100–250 bp were excised using a spatula. (C) Genomic library after PCR amplification using the gel extraction product as a template. Red arrow indicates the amplicon. (TIFF 33973 kb) [file 12864_2018_5375_MOESM4_ESM.tiff]

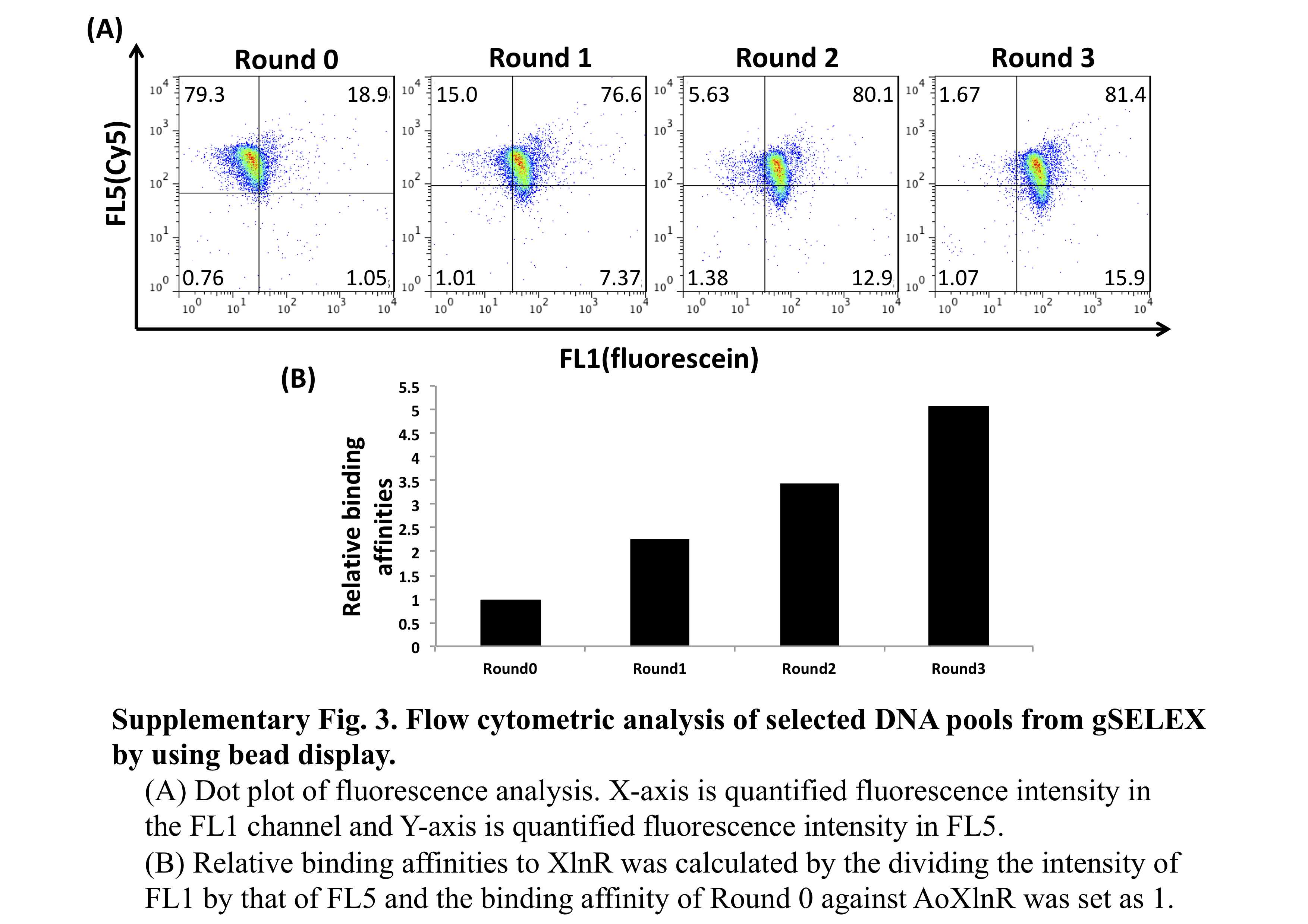

Supplement: Supplementary file 5 — Figure S3. Flow cytometric analysis of selected DNA pools from gSELEX by using bead display. (A) Dot plot of fluorescence analysis. X-axis is quantified fluorescence intensity in the FL1 channel and Y-axis is quantified fluorescence intensity in FL5. (B) Relative binding affinities to XlnR was calculated by the dividing the intensity of FL1 by that of FL5 and the binding affinity of Round 0 against AoXlnR was set as 1. (TIFF 33973 kb) [file 12864_2018_5375_MOESM5_ESM.tiff]

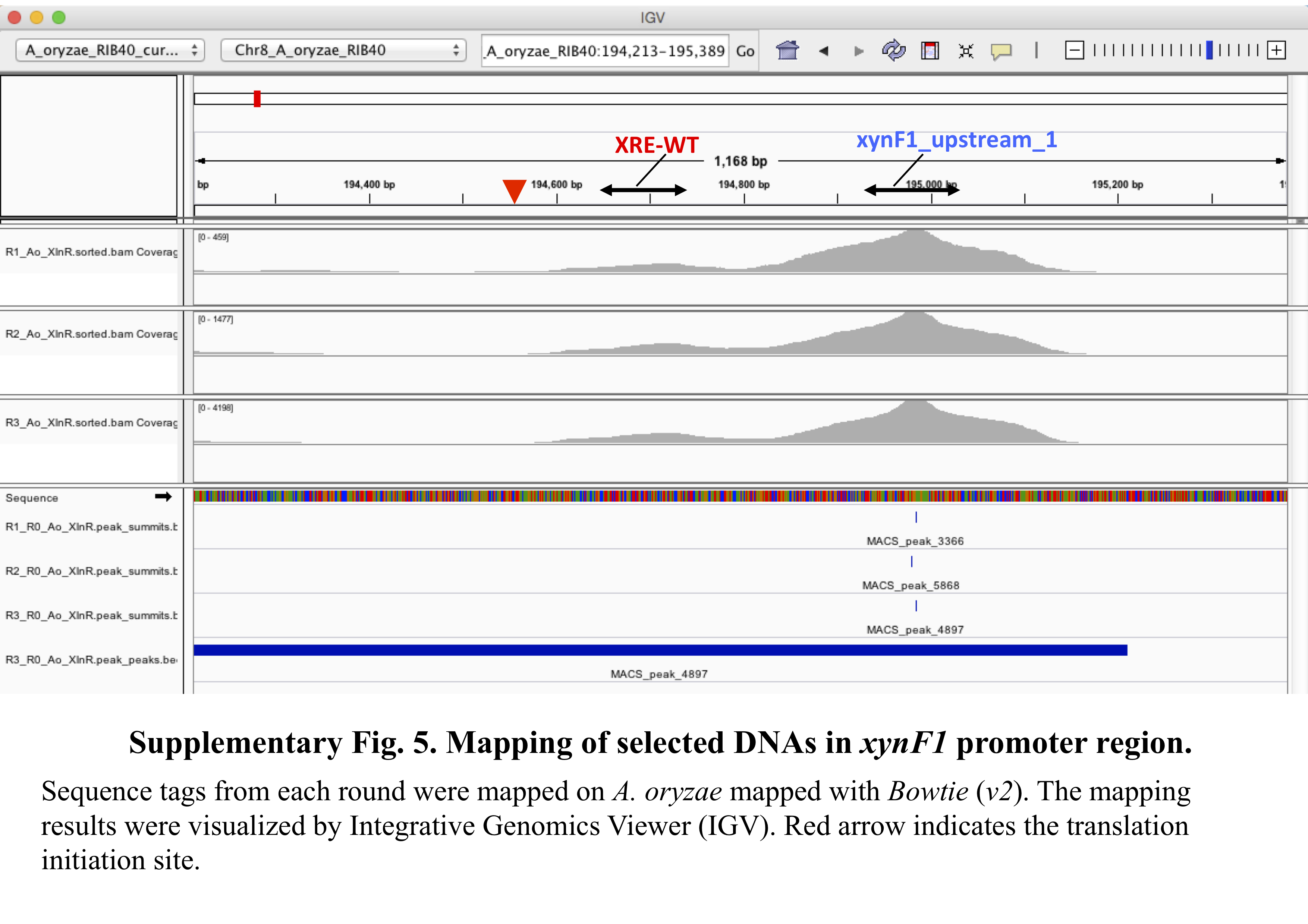

Supplement: Supplementary file 9 — Figure S5. Mapping of selected DNAs in xynF1 promoter region. Sequence tags from each round were mapped on A. oryzae mapped with Bowtie (v2). The mapping results were visualized by Integrative Genomics Viewer (IGV). Red arrow indicates the translation initiation site. (TIFF 33973 kb) [file 12864_2018_5375_MOESM9_ESM.tiff]
