# Supplementary material for: Comprehensive investigation of the gene expression system regulated by an Aspergillus oryzae transcription factor XlnR using integrated mining of gSELEX-Seq and microarray data
Source: BMC Genomics. 2019 Jan 8;20:16. doi: 10.1186/s12864-018-5375-5 (PMC6323846; doi:10.1186/s12864-018-5375-5)
Supplement: Supplementary file 6 — Table S3. A. oryzae promoter regions selected by gSELEX-Seq. (DOCX 239 kb) [file 12864_2018_5375_MOESM6_ESM.docx]

**Supplementary Table 3. *A. oryzae* promoter regions selected by gSELEX-Seq.**

| **Query ID** | **Subject ID** |
| --- | --- |
| Chr4_A_oryzae_RIB40:2303099-2303148 | AO090012000888 |
| Chr5_A_oryzae_RIB40:2756105-2756154 | AO090120000026 |
| Chr2_A_oryzae_RIB40:153223-153272 | AO090001000068 |
| Chr2_A_oryzae_RIB40:153223-153272 | AO090001000069 |
| Chr4_A_oryzae_RIB40:2315300-2315349 | AO090012000896 |
| Chr4_A_oryzae_RIB40:1789077-1789126 | AO090012000704 |
| Chr2_A_oryzae_RIB40:3051748-3051797 | AO090003000356 |
| Chr2_A_oryzae_RIB40:3051748-3051797 | AO090003000357 |
| Chr2_A_oryzae_RIB40:2627689-2627738 | AO090003000201 |
| Chr4_A_oryzae_RIB40:1811650-1811699 | AO090012000710 |
| Chr6_A_oryzae_RIB40:3229096-3229145 | AO090038000175 |
| Chr2_A_oryzae_RIB40:6010393-6010442 | AO090701000927 |
| Chr4_A_oryzae_RIB40:4512011-4512060 | AO090102000011 |
| Chr4_A_oryzae_RIB40:4512011-4512060 | AO090102000010 |
| Chr8_A_oryzae_RIB40:320754-320803 | AO090103000367 |
| Chr1_A_oryzae_RIB40:6305191-6305240 | AO090005000050 |
| Chr7_A_oryzae_RIB40:1904950-1904999 | AO090011000745 |
| Chr1_A_oryzae_RIB40:339446-339495 | AO090009000126 |
| Chr1_A_oryzae_RIB40:339446-339495 | AO090009000128 |
| Chr8_A_oryzae_RIB40:1875642-1875691 | AO090010000574 |
| Chr8_A_oryzae_RIB40:1875642-1875691 | AO090010000576 |
| Chr3_A_oryzae_RIB40:1110608-1110657 | AO090023000436 |
| Chr2_A_oryzae_RIB40:216121-216170 | AO090001000097 |
| Chr6_A_oryzae_RIB40:1157188-1157237 | AO090020000267 |
| Chr8_A_oryzae_RIB40:194960-195009 | AO090103000423 |
| Chr2_A_oryzae_RIB40:4424183-4424232 | AO090003000859 |
| Chr6_A_oryzae_RIB40:2972175-2972224 | AO090038000266 |
| Chr6_A_oryzae_RIB40:2972175-2972224 | AO090038000265 |
| Chr2_A_oryzae_RIB40:454048-454097 | AO090001000197 |
| Chr4_A_oryzae_RIB40:3407196-3407245 | AO090102000409 |
| Chr2_A_oryzae_RIB40:1713189-1713238 | AO090001000649 |
| Chr1_A_oryzae_RIB40:3793614-3793663 | AO090005000986 |
| Chr7_A_oryzae_RIB40:1210141-1210190 | AO090011000483 |
| Chr4_A_oryzae_RIB40:3319805-3319854 | AO090102000445 |
| Chr7_A_oryzae_RIB40:1013090-1013139 | AO090701001268 |
| Chr7_A_oryzae_RIB40:1013090-1013139 | AO090701001268 |
| Chr7_A_oryzae_RIB40:1013090-1013139 | AO090701001268 |
| Chr6_A_oryzae_RIB40:1323640-1323689 | AO090020000197 |
| Chr4_A_oryzae_RIB40:697462-697511 | AO090012000281 |
| Chr2_A_oryzae_RIB40:1205033-1205082 | AO090001000761 |
| Chr2_A_oryzae_RIB40:1205033-1205082 | AO090001000760 |
| Chr4_A_oryzae_RIB40:63121-63170 | AO090012000029 |
| Chr3_A_oryzae_RIB40:4758207-4758256 | AO090026000127 |
| Chr3_A_oryzae_RIB40:4758207-4758256 | AO090701000980 |
| Chr6_A_oryzae_RIB40:1701793-1701842 | AO090020000042 |
| Chr8_A_oryzae_RIB40:591433-591482 | AO090103000263 |
| Chr8_A_oryzae_RIB40:591433-591482 | AO090103000264 |
| Chr5_A_oryzae_RIB40:3197133-3197182 | AO090120000203 |
| Chr4_A_oryzae_RIB40:958771-958820 | AO090012000389 |
| Chr8_A_oryzae_RIB40:2020821-2020870 | AO090010000515 |
| Chr5_A_oryzae_RIB40:601834-601883 | AO090701000674 |
| Chr7_A_oryzae_RIB40:2331730-2331779 | AO090011000900 |
| Chr2_A_oryzae_RIB40:5913957-5914006 | AO090003001417 |
| Chr2_A_oryzae_RIB40:5913957-5914006 | AO090003001418 |
| Chr4_A_oryzae_RIB40:1113777-1113826 | AO090012000445 |
| Chr3_A_oryzae_RIB40:736366-736415 | AO090023000285 |
| Chr7_A_oryzae_RIB40:279330-279379 | AO090011000096 |
| Chr6_A_oryzae_RIB40:753471-753520 | AO090020000429 |
| Chr1_A_oryzae_RIB40:5080072-5080121 | AO090005000512 |
| Chr8_A_oryzae_RIB40:1517626-1517675 | AO090010000726 |
| Chr8_A_oryzae_RIB40:1907384-1907433 | AO090010000562 |
| Chr8_A_oryzae_RIB40:2151594-2151643 | AO090010000464 |
| Chr8_A_oryzae_RIB40:1038005-1038054 | AO090103000087 |
| Chr3_A_oryzae_RIB40:2635568-2635617 | AO090023000997 |
| Chr8_A_oryzae_RIB40:2283317-2283366 | AO090010000406 |
| Chr8_A_oryzae_RIB40:2283317-2283366 | AO090010000407 |
| Chr6_A_oryzae_RIB40:448374-448423 | AO090020000538 |
| Chr4_A_oryzae_RIB40:4101349-4101398 | AO090102000170 |
| Chr1_A_oryzae_RIB40:1612686-1612735 | AO090009000603 |
| Chr8_A_oryzae_RIB40:1464499-1464548 | AO090010000743 |
| Chr6_A_oryzae_RIB40:182441-182490 | AO090020000646 |
| Chr1_A_oryzae_RIB40:4581138-4581187 | AO090005000698 |
| Chr4_A_oryzae_RIB40:3520133-3520182 | AO090102000372 |
| Chr2_A_oryzae_RIB40:4801890-4801939 | AO090003000997 |
| Chr6_A_oryzae_RIB40:1771333-1771382 | AO090020000021 |
| Chr6_A_oryzae_RIB40:1771333-1771382 | AO090020000022 |
| Chr1_A_oryzae_RIB40:2511622-2511671 | AO090005001482 |
| Chr8_A_oryzae_RIB40:418309-418358 | AO090103000336 |
| Chr3_A_oryzae_RIB40:89174-89223 | AO090023000034 |
| Chr1_A_oryzae_RIB40:4367701-4367750 | AO090005000769 |
| Chr1_A_oryzae_RIB40:4367701-4367750 | AO090701000933 |
| Chr3_A_oryzae_RIB40:987689-987738 | AO090023000384 |
| Chr6_A_oryzae_RIB40:1150517-1150566 | AO090020000269 |
| Chr5_A_oryzae_RIB40:1517661-1517710 | AO090701000315 |
| Chr1_A_oryzae_RIB40:110714-110763 | AO090009000039 |
| Chr3_A_oryzae_RIB40:4895496-4895545 | AO090026000083 |
| Chr1_A_oryzae_RIB40:3986855-3986904 | AO090005000912 |
| Chr5_A_oryzae_RIB40:1482023-1482072 | AO090701000327 |
| Chr7_A_oryzae_RIB40:388692-388741 | AO090011000141 |
| Chr7_A_oryzae_RIB40:388692-388741 | AO090011000140 |
| Chr7_A_oryzae_RIB40:1061147-1061196 | AO090011000416 |
| Chr3_A_oryzae_RIB40:4722583-4722632 | AO090026000142 |
| Chr5_A_oryzae_RIB40:3788641-3788690 | AO090120000413 |
| Chr5_A_oryzae_RIB40:3016883-3016932 | AO090120000135 |
| Chr6_A_oryzae_RIB40:1472299-1472348 | AO090020000136 |
| Chr5_A_oryzae_RIB40:1035102-1035151 | AO090701000503 |
| Chr7_A_oryzae_RIB40:1417660-1417709 | AO090011000971 |
| Chr3_A_oryzae_RIB40:1030635-1030684 | AO090023000401 |
| Chr2_A_oryzae_RIB40:253875-253924 | AO090001000111 |
| Chr4_A_oryzae_RIB40:330414-330463 | AO090012000135 |
| Chr2_A_oryzae_RIB40:2103887-2103936 | AO090003000002 |
| Chr2_A_oryzae_RIB40:6156464-6156513 | AO090003001517 |
| Chr4_A_oryzae_RIB40:3656077-3656126 | AO090102000326 |
| Chr5_A_oryzae_RIB40:55158-55207 | AO090701000884 |
| Chr7_A_oryzae_RIB40:1699923-1699972 | AO090011000654 |
| Chr2_A_oryzae_RIB40:3615945-3615994 | AO090003000570 |
| Chr4_A_oryzae_RIB40:4030750-4030799 | AO090102000199 |
| Chr4_A_oryzae_RIB40:4770669-4770718 | AO090166000075 |
| Chr3_A_oryzae_RIB40:1354-1403 | AO090023000001 |
| Chr4_A_oryzae_RIB40:4178913-4178962 | AO090102000141 |
| Chr2_A_oryzae_RIB40:859352-859401 | AO090001000343 |
| Chr1_A_oryzae_RIB40:2231855-2231904 | AO090005001567 |
| Chr1_A_oryzae_RIB40:2231855-2231904 | AO090005001568 |
| Chr1_A_oryzae_RIB40:401651-401700 | AO090009000148 |
| Chr1_A_oryzae_RIB40:5341529-5341578 | AO090005000423 |
| Chr6_A_oryzae_RIB40:2340750-2340799 | AO090038000495 |
| Chr5_A_oryzae_RIB40:1553440-1553489 | AO090701001006 |
| Chr3_A_oryzae_RIB40:3739372-3739421 | AO090026000490 |
| Chr3_A_oryzae_RIB40:3739372-3739421 | AO090701001131 |
| Chr5_A_oryzae_RIB40:2320850-2320899 | AO090701000015 |
| Chr3_A_oryzae_RIB40:1498880-1498929 | AO090023000568 |
| Chr1_A_oryzae_RIB40:5922902-5922951 | AO090005000189 |
| Chr1_A_oryzae_RIB40:5417343-5417392 | AO090005000395 |
| Chr3_A_oryzae_RIB40:2526496-2526545 | AO090023000957 |
| Chr6_A_oryzae_RIB40:290286-290335 | AO090020000604 |
| Chr8_A_oryzae_RIB40:3001604-3001653 | AO090010000153 |
| Chr6_A_oryzae_RIB40:1534134-1534183 | AO090020000107 |
| Chr6_A_oryzae_RIB40:3561295-3561344 | AO090038000039 |
| Chr4_A_oryzae_RIB40:3629423-3629472 | AO090102000336 |
| Chr1_A_oryzae_RIB40:5107388-5107437 | AO090005000503 |
| Chr5_A_oryzae_RIB40:165067-165116 | AO090701000917 |
| Chr8_A_oryzae_RIB40:580751-580800 | AO090103000267 |
| Chr8_A_oryzae_RIB40:580751-580800 | AO090103000268 |
| Chr4_A_oryzae_RIB40:1709389-1709438 | AO090012000671 |
| Chr7_A_oryzae_RIB40:2712605-2712654 | AO090206000054 |
| Chr8_A_oryzae_RIB40:300408-300457 | AO090103000376 |
| Chr2_A_oryzae_RIB40:2773414-2773463 | AO090003000256 |
| Chr6_A_oryzae_RIB40:2536634-2536683 | AO090038000425 |
| Chr2_A_oryzae_RIB40:1401266-1401315 | AO090001000536 |
| Chr2_A_oryzae_RIB40:3681093-3681142 | AO090003000593 |
| Chr2_A_oryzae_RIB40:3681093-3681142 | AO090003000594 |
| Chr3_A_oryzae_RIB40:679232-679281 | AO090023000264 |
| Chr1_A_oryzae_RIB40:3781654-3781703 | AO090005000990 |
| Chr5_A_oryzae_RIB40:1683400-1683449 | AO090701000250 |
| Chr4_A_oryzae_RIB40:737346-737395 | AO090012000298 |
| Chr4_A_oryzae_RIB40:1017784-1017833 | AO090012000414 |
| Chr7_A_oryzae_RIB40:1212510-1212559 | AO090011000486 |
| Chr2_A_oryzae_RIB40:1584567-1584616 | AO090701001074 |
| Chr6_A_oryzae_RIB40:2314336-2314385 | AO090038000504 |
| Chr2_A_oryzae_RIB40:1405622-1405671 | AO090001000539 |
| Chr5_A_oryzae_RIB40:1002077-1002126 | AO090701000515 |
| Chr5_A_oryzae_RIB40:4512303-4512352 | AO090113000199 |
| Chr2_A_oryzae_RIB40:479763-479812 | AO090001000207 |
| Chr5_A_oryzae_RIB40:1417141-1417190 | AO090701000345 |
| Chr5_A_oryzae_RIB40:1417141-1417190 | AO090701000346 |
| Chr5_A_oryzae_RIB40:3570436-3570485 | AO090120000331 |
| Chr2_A_oryzae_RIB40:1534056-1534105 | AO090001000585 |
| Chr2_A_oryzae_RIB40:1534056-1534105 | AO090001000584 |
| Chr2_A_oryzae_RIB40:2236821-2236870 | AO090003000055 |
| Chr2_A_oryzae_RIB40:2236821-2236870 | AO090003000056 |
| Chr6_A_oryzae_RIB40:3496191-3496240 | AO090038000067 |
| Chr6_A_oryzae_RIB40:3496191-3496240 | AO090038000066 |
| Chr2_A_oryzae_RIB40:482537-482586 | AO090001000208 |
| Chr2_A_oryzae_RIB40:5630160-5630209 | AO090003001305 |
| Chr8_A_oryzae_RIB40:1557274-1557323 | AO090010000706 |
| Chr3_A_oryzae_RIB40:4893612-4893661 | AO090026000084 |
| Chr3_A_oryzae_RIB40:4893612-4893661 | AO090003000903 |
| Chr4_A_oryzae_RIB40:45122-45171 | AO090012000020 |
| Chr4_A_oryzae_RIB40:462062-462111 | AO090012000184 |
| Chr3_A_oryzae_RIB40:401736-401785 | AO090023000158 |
| Chr3_A_oryzae_RIB40:3757960-3758009 | AO090026000484 |
| Chr5_A_oryzae_RIB40:3759257-3759306 | AO090120000402 |
| Chr2_A_oryzae_RIB40:2424553-2424602 | AO090003000121 |
| Chr2_A_oryzae_RIB40:2424553-2424602 | AO090003000122 |
| Chr5_A_oryzae_RIB40:52284-52333 | AO090701000885 |
| Chr8_A_oryzae_RIB40:2504532-2504581 | AO090010000314 |
| Chr3_A_oryzae_RIB40:28705-28754 | AO090023000010 |
| Chr3_A_oryzae_RIB40:903831-903880 | AO090023000354 |
| Chr3_A_oryzae_RIB40:4005002-4005051 | AO090026000394 |
| Chr5_A_oryzae_RIB40:89429-89478 | AO090701000870 |
| Chr8_A_oryzae_RIB40:2622285-2622334 | AO090026000084 |
| Chr8_A_oryzae_RIB40:2622285-2622334 | AO090003000903 |
| Chr5_A_oryzae_RIB40:4034589-4034638 | AO090120000506 |
| Chr8_A_oryzae_RIB40:3188715-3188764 | AO090010000085 |
| Chr5_A_oryzae_RIB40:1313994-1314043 | AO090701000391 |
| Chr6_A_oryzae_RIB40:4092055-4092104 | AO090138000050 |
| Chr4_A_oryzae_RIB40:4492026-4492075 | AO090102000020 |
| Chr3_A_oryzae_RIB40:2585421-2585470 | AO090023000977 |
| Chr3_A_oryzae_RIB40:2585421-2585470 | AO090023000979 |
| Chr3_A_oryzae_RIB40:563149-563198 | AO090023000226 |
| Chr2_A_oryzae_RIB40:3194223-3194272 | AO090003000417 |
| Chr3_A_oryzae_RIB40:1235031-1235080 | AO090023000481 |
| Chr1_A_oryzae_RIB40:6423487-6423536 | AO090005000002 |
| Chr1_A_oryzae_RIB40:6423487-6423536 | AO090005000001 |
| Chr2_A_oryzae_RIB40:4823753-4823802 | AO090003001006 |
| Chr4_A_oryzae_RIB40:1277265-1277314 | AO090012000504 |
| Chr7_A_oryzae_RIB40:1090506-1090555 | AO090011000430 |
| Chr8_A_oryzae_RIB40:857570-857619 | AO090103000160 |
| Chr1_A_oryzae_RIB40:1193992-1194041 | AO090701000434 |
| Chr6_A_oryzae_RIB40:311892-311941 | AO090020000596 |
| Chr6_A_oryzae_RIB40:311892-311941 | AO090020000595 |
| Chr7_A_oryzae_RIB40:1057820-1057869 | AO090011000414 |
| Chr3_A_oryzae_RIB40:1629119-1629168 | AO090023000622 |
| Chr1_A_oryzae_RIB40:1162166-1162215 | AO090009000446 |
| Chr5_A_oryzae_RIB40:464176-464225 | AO090701000717 |
| Chr6_A_oryzae_RIB40:2262653-2262702 | AO090038000524 |
| Chr4_A_oryzae_RIB40:812841-812890 | AO090012000331 |
| Chr4_A_oryzae_RIB40:812841-812890 | AO090012000330 |
| Chr1_A_oryzae_RIB40:4198559-4198608 | AO090005000829 |
| Chr5_A_oryzae_RIB40:2008649-2008698 | AO090701000123 |
| Chr4_A_oryzae_RIB40:3903668-3903717 | AO090102000239 |
| Chr3_A_oryzae_RIB40:4976592-4976641 | AO090026000056 |
| Chr7_A_oryzae_RIB40:665898-665947 | AO090011000261 |
| Chr5_A_oryzae_RIB40:3091678-3091727 | AO090120000158 |
| Chr8_A_oryzae_RIB40:190101-190150 | AO090103000426 |
| Chr1_A_oryzae_RIB40:6240419-6240468 | AO090005000072 |
| Chr3_A_oryzae_RIB40:4621412-4621461 | AO090026000177 |
| Chr2_A_oryzae_RIB40:1435788-1435837 | AO090001000550 |
| Chr3_A_oryzae_RIB40:1193335-1193384 | AO090023000465 |
| Chr4_A_oryzae_RIB40:2421-2470 | AO090012000001 |
| Chr4_A_oryzae_RIB40:2486811-2486860 | AO090012000958 |
| Chr4_A_oryzae_RIB40:2486811-2486860 | AO090012000957 |
| Chr1_A_oryzae_RIB40:372257-372306 | AO090009000138 |
| Chr5_A_oryzae_RIB40:903431-903480 | AO090701000547 |
| Chr5_A_oryzae_RIB40:4137306-4137355 | AO090113000040 |
| Chr3_A_oryzae_RIB40:1625905-1625954 | AO090023000620 |
| Chr3_A_oryzae_RIB40:1625905-1625954 | AO090023000621 |
| Chr2_A_oryzae_RIB40:67534-67583 | AO090001000029 |
| Chr5_A_oryzae_RIB40:3171283-3171332 | AO090120000193 |
| Chr5_A_oryzae_RIB40:3171283-3171332 | AO090120000194 |
| Chr7_A_oryzae_RIB40:360071-360120 | AO090011000129 |
| Chr7_A_oryzae_RIB40:986723-986772 | AO090011000385 |
| Chr2_A_oryzae_RIB40:4545311-4545360 | AO090003000905 |
| Chr2_A_oryzae_RIB40:4545311-4545360 | AO090003000904 |
| Chr8_A_oryzae_RIB40:2155355-2155404 | AO090010000463 |
| Chr3_A_oryzae_RIB40:176641-176690 | AO090023000064 |
| Chr3_A_oryzae_RIB40:2886610-2886659 | AO090026000820 |
| Chr2_A_oryzae_RIB40:4635110-4635159 | AO090003000935 |
| Chr7_A_oryzae_RIB40:1201516-1201565 | AO090026000084 |
| Chr7_A_oryzae_RIB40:1201516-1201565 | AO090003000903 |
| Chr8_A_oryzae_RIB40:2848871-2848920 | AO090701001294 |
| Chr6_A_oryzae_RIB40:2985127-2985176 | AO090038000260 |
| Chr6_A_oryzae_RIB40:2985127-2985176 | AO090038000259 |
| Chr3_A_oryzae_RIB40:702477-702526 | AO090701001118 |
| Chr8_A_oryzae_RIB40:2349418-2349467 | AO090010000375 |
| Chr8_A_oryzae_RIB40:2349418-2349467 | AO090010000374 |
| Chr8_A_oryzae_RIB40:2781194-2781243 | AO090010000231 |
| Chr7_A_oryzae_RIB40:193371-193420 | AO090011000063 |
| Chr2_A_oryzae_RIB40:278108-278157 | AO090001000119 |
| Chr7_A_oryzae_RIB40:534469-534518 | AO090011000205 |
| Chr6_A_oryzae_RIB40:1284834-1284883 | AO090020000216 |
| Chr6_A_oryzae_RIB40:1284834-1284883 | AO090020000215 |
| Chr4_A_oryzae_RIB40:2283798-2283847 | AO090012000881 |
| Chr4_A_oryzae_RIB40:2283798-2283847 | AO090012000880 |
| Chr4_A_oryzae_RIB40:581435-581484 | AO090012000236 |
| Chr6_A_oryzae_RIB40:2306456-2306505 | AO090038000508 |
| Chr6_A_oryzae_RIB40:4105698-4105747 | AO090138000042 |
| Chr6_A_oryzae_RIB40:1470529-1470578 | AO090020000137 |
| Chr7_A_oryzae_RIB40:340573-340622 | AO090011000121 |
| Chr8_A_oryzae_RIB40:2144630-2144679 | AO090010000468 |
| Chr5_A_oryzae_RIB40:690187-690236 | AO090701000639 |
| Chr5_A_oryzae_RIB40:4225093-4225142 | AO090701001215 |
| Chr4_A_oryzae_RIB40:222690-222739 | AO090012000090 |
| Chr1_A_oryzae_RIB40:5674524-5674573 | AO090005000286 |
| Chr5_A_oryzae_RIB40:1200575-1200624 | AO090701000434 |
| Chr2_A_oryzae_RIB40:4002228-4002277 | AO090003000707 |
| Chr1_A_oryzae_RIB40:444157-444206 | AO090009000168 |
| Chr3_A_oryzae_RIB40:1485770-1485819 | AO090023000562 |
| Chr7_A_oryzae_RIB40:1171308-1171357 | AO090011000962 |
| Chr8_A_oryzae_RIB40:3235124-3235173 | AO090010000063 |
| Chr1_A_oryzae_RIB40:5136487-5136536 | AO090005000491 |
| Chr1_A_oryzae_RIB40:5136487-5136536 | AO090005000490 |
| Chr2_A_oryzae_RIB40:5557210-5557259 | AO090003001277 |
| Chr2_A_oryzae_RIB40:6122232-6122281 | AO090003001580 |
| Chr3_A_oryzae_RIB40:4710111-4710160 | AO090701001139 |
| Chr4_A_oryzae_RIB40:1899086-1899135 | AO090012000746 |
| Chr4_A_oryzae_RIB40:4043970-4044019 | AO090102000192 |
| Chr8_A_oryzae_RIB40:1536214-1536263 | AO090010000786 |
| Chr8_A_oryzae_RIB40:225483-225532 | AO090103000413 |
| Chr5_A_oryzae_RIB40:210300-210349 | AO090701000824 |
| Chr8_A_oryzae_RIB40:3345100-3345149 | AO090010000023 |
| Chr1_A_oryzae_RIB40:4599183-4599232 | AO090005000692 |
| Chr1_A_oryzae_RIB40:4599183-4599232 | AO090005000693 |
| Chr8_A_oryzae_RIB40:1608206-1608255 | AO090010000688 |
| Chr5_A_oryzae_RIB40:1618103-1618152 | AO090701000274 |
| Chr6_A_oryzae_RIB40:1367998-1368047 | AO090020000186 |
| Chr2_A_oryzae_RIB40:3333479-3333528 | AO090003000463 |
| Chr3_A_oryzae_RIB40:652957-653006 | AO090023000254 |
| Chr3_A_oryzae_RIB40:652957-653006 | AO090023000255 |
| Chr8_A_oryzae_RIB40:3338394-3338443 | AO090010000026 |
| Chr2_A_oryzae_RIB40:4799671-4799720 | AO090003000996 |
| Chr1_A_oryzae_RIB40:132646-132695 | AO090009000049 |
| Chr1_A_oryzae_RIB40:2938643-2938692 | AO090005001299 |
| Chr1_A_oryzae_RIB40:2938643-2938692 | AO090005001298 |
| Chr4_A_oryzae_RIB40:385818-385867 | AO090012000162 |
| Chr4_A_oryzae_RIB40:385818-385867 | AO090012000161 |
| Chr4_A_oryzae_RIB40:2527977-2528026 | AO090012000974 |
| Chr4_A_oryzae_RIB40:2527977-2528026 | AO090012000975 |
| Chr1_A_oryzae_RIB40:4628784-4628833 | AO090005000684 |
| Chr6_A_oryzae_RIB40:2398021-2398070 | AO090038000472 |
| Chr3_A_oryzae_RIB40:3678576-3678625 | AO090026000515 |
| Chr3_A_oryzae_RIB40:3678576-3678625 | AO090026000514 |
| Chr2_A_oryzae_RIB40:1379986-1380035 | AO090001000528 |
| Chr1_A_oryzae_RIB40:5570618-5570667 | AO090005000336 |
| Chr1_A_oryzae_RIB40:5819024-5819073 | AO090005000232 |
| Chr3_A_oryzae_RIB40:3204980-3205029 | AO090026000701 |
| Chr2_A_oryzae_RIB40:5594101-5594150 | AO090003001293 |
| Chr4_A_oryzae_RIB40:1885118-1885167 | AO090012000737 |
| Chr4_A_oryzae_RIB40:1885118-1885167 | AO090012000738 |
| Chr3_A_oryzae_RIB40:122314-122363 | AO090023000047 |
| Chr8_A_oryzae_RIB40:440799-440848 | AO090103000326 |
| Chr6_A_oryzae_RIB40:4149326-4149375 | AO090138000206 |
| Chr5_A_oryzae_RIB40:2857473-2857522 | AO090120000076 |
| Chr5_A_oryzae_RIB40:2857473-2857522 | AO090120000075 |
| Chr1_A_oryzae_RIB40:4073152-4073201 | AO090005000883 |
| Chr5_A_oryzae_RIB40:156723-156772 | AO090701000841 |
| Chr3_A_oryzae_RIB40:4992241-4992290 | AO090026000049 |
| Chr3_A_oryzae_RIB40:4992241-4992290 | AO090026000050 |
| Chr2_A_oryzae_RIB40:634878-634927 | AO090701001064 |
| Chr8_A_oryzae_RIB40:781692-781741 | AO090103000192 |
| Chr2_A_oryzae_RIB40:4195265-4195314 | AO090003000782 |
| Chr2_A_oryzae_RIB40:4195265-4195314 | AO090003000781 |
| Chr4_A_oryzae_RIB40:4396942-4396991 | AO090102000058 |
| Chr1_A_oryzae_RIB40:6349227-6349276 | AO090005000031 |
| Chr5_A_oryzae_RIB40:2561020-2561069 | AO090124000094 |
| Chr1_A_oryzae_RIB40:2121669-2121718 | AO090005001607 |
| Chr1_A_oryzae_RIB40:2121669-2121718 | AO090005001606 |
| Chr3_A_oryzae_RIB40:3641367-3641416 | AO090026000526 |
| Chr3_A_oryzae_RIB40:3641367-3641416 | AO090026000527 |
| Chr8_A_oryzae_RIB40:1035222-1035271 | AO090103000088 |
| Chr6_A_oryzae_RIB40:4178565-4178614 | AO090138000006 |
| Chr7_A_oryzae_RIB40:46713-46762 | AO090011000957 |
| Chr4_A_oryzae_RIB40:4006440-4006489 | AO090701000434 |
| Chr6_A_oryzae_RIB40:1129150-1129199 | AO090020000277 |
| Chr7_A_oryzae_RIB40:1055681-1055730 | AO090011000413 |
| Chr2_A_oryzae_RIB40:539437-539486 | AO090001000224 |
| Chr5_A_oryzae_RIB40:4519570-4519619 | AO090113000204 |
| Chr4_A_oryzae_RIB40:155350-155399 | AO090012000067 |
| Chr2_A_oryzae_RIB40:2921353-2921402 | AO090003000305 |
| Chr3_A_oryzae_RIB40:2150611-2150660 | AO090701000434 |
| Chr7_A_oryzae_RIB40:2440915-2440964 | AO090011000928 |
| Chr5_A_oryzae_RIB40:4458050-4458099 | AO090113000178 |
| Chr6_A_oryzae_RIB40:1942044-1942093 | AO090038000632 |
| Chr6_A_oryzae_RIB40:1942044-1942093 | AO090038000631 |
| Chr1_A_oryzae_RIB40:222797-222846 | AO090009000076 |
| Chr6_A_oryzae_RIB40:3327914-3327963 | AO090038000137 |
| Chr6_A_oryzae_RIB40:3327914-3327963 | AO090010000290 |
| Chr6_A_oryzae_RIB40:3327914-3327963 | AO090026000545 |
| Chr6_A_oryzae_RIB40:3327914-3327963 | AO090010000292 |
| Chr1_A_oryzae_RIB40:2857636-2857685 | AO090005001331 |
| Chr3_A_oryzae_RIB40:4903671-4903720 | AO090026000080 |
| Chr2_A_oryzae_RIB40:5873139-5873188 | AO090003001402 |
| Chr2_A_oryzae_RIB40:5873139-5873188 | AO090003001401 |
| Chr1_A_oryzae_RIB40:1444272-1444321 | AO090009000545 |
| Chr1_A_oryzae_RIB40:3162722-3162771 | AO090005001231 |
| Chr5_A_oryzae_RIB40:3614261-3614310 | AO090120000347 |
| Chr4_A_oryzae_RIB40:3728780-3728829 | AO090102000296 |
| Chr3_A_oryzae_RIB40:209600-209649 | AO090023000080 |
| Chr5_A_oryzae_RIB40:4265278-4265327 | AO090113000090 |
| Chr1_A_oryzae_RIB40:3811777-3811826 | AO090005000980 |
| Chr1_A_oryzae_RIB40:2516334-2516383 | AO090005001479 |
| Chr6_A_oryzae_RIB40:2903507-2903556 | AO090038000287 |
| Chr2_A_oryzae_RIB40:97458-97507 | AO090001000043 |
| Chr3_A_oryzae_RIB40:4330371-4330420 | AO090026000278 |
| Chr6_A_oryzae_RIB40:3467799-3467848 | AO090038000082 |
| Chr6_A_oryzae_RIB40:3467799-3467848 | AO090038000081 |
| Chr3_A_oryzae_RIB40:3634842-3634891 | AO090026000529 |
| Chr7_A_oryzae_RIB40:800510-800559 | AO090011000317 |
| Chr7_A_oryzae_RIB40:485192-485241 | AO090011000183 |
| Chr7_A_oryzae_RIB40:1457365-1457414 | AO090011000576 |
| Chr3_A_oryzae_RIB40:1697448-1697497 | AO090023000646 |
| Chr8_A_oryzae_RIB40:2426213-2426262 | AO090010000349 |
| Chr7_A_oryzae_RIB40:201751-201800 | AO090011000067 |
| Chr7_A_oryzae_RIB40:201751-201800 | AO090011000066 |
| Chr3_A_oryzae_RIB40:2881074-2881123 | AO090026000824 |
| Chr6_A_oryzae_RIB40:3199333-3199382 | AO090038000185 |
| Chr7_A_oryzae_RIB40:1592959-1593008 | AO090011000620 |
| Chr2_A_oryzae_RIB40:973922-973971 | AO090001000386 |
| Chr6_A_oryzae_RIB40:246131-246180 | AO090020000618 |
| Chr2_A_oryzae_RIB40:4835634-4835683 | AO090003001013 |
| Chr2_A_oryzae_RIB40:4835634-4835683 | AO090003001014 |
| Chr7_A_oryzae_RIB40:1062051-1062100 | AO090011000417 |
| Chr1_A_oryzae_RIB40:262896-262945 | AO090009000092 |
| Chr2_A_oryzae_RIB40:2649486-2649535 | AO090003000208 |
| Chr4_A_oryzae_RIB40:2625475-2625524 | AO090012001015 |
| Chr4_A_oryzae_RIB40:3057394-3057443 | AO090102000540 |
| Chr4_A_oryzae_RIB40:3057394-3057443 | AO090102000541 |
| Chr5_A_oryzae_RIB40:2795384-2795433 | AO090124000093 |
| Chr4_A_oryzae_RIB40:664948-664997 | AO090012000268 |
| Chr6_A_oryzae_RIB40:2976317-2976366 | AO090038000263 |
| Chr6_A_oryzae_RIB40:2976317-2976366 | AO090038000264 |
| Chr2_A_oryzae_RIB40:4435635-4435684 | AO090003000863 |
| Chr3_A_oryzae_RIB40:4410545-4410594 | AO090026000252 |
| Chr7_A_oryzae_RIB40:780651-780700 | AO090011000307 |
| Chr7_A_oryzae_RIB40:780651-780700 | AO090011000308 |
| Chr8_A_oryzae_RIB40:2220881-2220930 | AO090010000437 |
| Chr3_A_oryzae_RIB40:249459-249508 | AO090023000100 |
| Chr3_A_oryzae_RIB40:249459-249508 | AO090023000099 |
| Chr1_A_oryzae_RIB40:3318545-3318594 | AO090005001172 |
| Chr1_A_oryzae_RIB40:3318545-3318594 | AO090005001171 |
| Chr5_A_oryzae_RIB40:4103726-4103775 | AO090113000022 |
| Chr7_A_oryzae_RIB40:418001-418050 | AO090011000153 |
| Chr1_A_oryzae_RIB40:274569-274618 | AO090009000097 |
| Chr1_A_oryzae_RIB40:5652642-5652691 | AO090005000297 |
| Chr7_A_oryzae_RIB40:1259661-1259710 | AO090011000502 |
| Chr3_A_oryzae_RIB40:4056664-4056713 | AO090026000372 |
| Chr1_A_oryzae_RIB40:645404-645453 | AO090009000242 |
| Chr2_A_oryzae_RIB40:2349978-2350027 | AO090003000098 |
| Chr6_A_oryzae_RIB40:1028899-1028948 | AO090020000320 |
| Chr1_A_oryzae_RIB40:4632720-4632769 | AO090005000682 |
| Chr1_A_oryzae_RIB40:4632720-4632769 | AO090005000683 |
| Chr2_A_oryzae_RIB40:5929783-5929832 | AO090003001424 |
| Chr2_A_oryzae_RIB40:5929783-5929832 | AO090003001423 |
| Chr4_A_oryzae_RIB40:4413116-4413165 | AO090102000052 |
| Chr8_A_oryzae_RIB40:3182421-3182470 | AO090010000088 |
| Chr5_A_oryzae_RIB40:799213-799262 | AO090701000590 |
| Chr4_A_oryzae_RIB40:2338293-2338342 | AO090012000904 |
| Chr5_A_oryzae_RIB40:1969838-1969887 | AO090701000141 |
| Chr1_A_oryzae_RIB40:3314325-3314374 | AO090005001174 |
| Chr1_A_oryzae_RIB40:819552-819601 | AO090009000307 |
| Chr1_A_oryzae_RIB40:1849736-1849785 | AO090009000690 |
| Chr6_A_oryzae_RIB40:1241537-1241586 | AO090020000231 |
| Chr1_A_oryzae_RIB40:5127016-5127065 | AO090005000494 |
| Chr1_A_oryzae_RIB40:2496714-2496763 | AO090005001484 |
| Chr2_A_oryzae_RIB40:1402264-1402313 | AO090001000537 |
| Chr2_A_oryzae_RIB40:1402264-1402313 | AO090001000538 |
| Chr1_A_oryzae_RIB40:1741755-1741804 | AO090701000434 |
| Chr2_A_oryzae_RIB40:1555653-1555702 | AO090001000594 |
| Chr4_A_oryzae_RIB40:1087794-1087843 | AO090012000436 |
| Chr5_A_oryzae_RIB40:151120-151169 | AO090701000843 |
| Chr5_A_oryzae_RIB40:411989-412038 | AO090701000739 |
| Chr5_A_oryzae_RIB40:2668384-2668433 | AO090124000007 |
| Chr6_A_oryzae_RIB40:369682-369731 | AO090020000571 |
| Chr6_A_oryzae_RIB40:369682-369731 | AO090020000572 |
| Chr6_A_oryzae_RIB40:3227757-3227806 | AO090038000176 |
| Chr5_A_oryzae_RIB40:1697101-1697150 | AO090701000244 |
| Chr1_A_oryzae_RIB40:1425805-1425854 | AO090009000535 |
| Chr1_A_oryzae_RIB40:1425805-1425854 | AO090009000536 |
| Chr1_A_oryzae_RIB40:2847696-2847745 | AO090005001339 |
| Chr1_A_oryzae_RIB40:2847696-2847745 | AO090005001338 |
| Chr1_A_oryzae_RIB40:2644061-2644110 | AO090005001427 |
| Chr8_A_oryzae_RIB40:2543059-2543108 | AO090038000137 |
| Chr8_A_oryzae_RIB40:2543059-2543108 | AO090010000290 |
| Chr8_A_oryzae_RIB40:2543059-2543108 | AO090026000545 |
| Chr8_A_oryzae_RIB40:2543059-2543108 | AO090010000292 |
| Chr2_A_oryzae_RIB40:5374869-5374918 | AO090003001206 |
| Chr5_A_oryzae_RIB40:1933620-1933669 | AO090701001197 |
| Chr8_A_oryzae_RIB40:2802687-2802736 | AO090010000225 |
| Chr8_A_oryzae_RIB40:420439-420488 | AO090103000335 |
| Chr2_A_oryzae_RIB40:3025488-3025537 | AO090003000346 |
| Chr2_A_oryzae_RIB40:3025488-3025537 | AO090003000344 |
| Chr2_A_oryzae_RIB40:659704-659753 | AO090001000265 |
| Chr2_A_oryzae_RIB40:1489473-1489522 | AO090001000567 |
| Chr7_A_oryzae_RIB40:241790-241839 | AO090011000084 |
| Chr3_A_oryzae_RIB40:1591101-1591150 | AO090023000604 |
| Chr4_A_oryzae_RIB40:318615-318664 | AO090012000131 |
| Chr4_A_oryzae_RIB40:318615-318664 | AO090012000132 |
| Chr3_A_oryzae_RIB40:1652537-1652586 | AO090023000629 |
| Chr3_A_oryzae_RIB40:1652537-1652586 | AO090023000628 |
| Chr7_A_oryzae_RIB40:1052162-1052211 | AO090011000411 |
| Chr3_A_oryzae_RIB40:580907-580956 | AO090023000231 |
| Chr5_A_oryzae_RIB40:525913-525962 | AO090701000699 |
| Chr5_A_oryzae_RIB40:435260-435309 | AO090701000728 |
| Chr5_A_oryzae_RIB40:435260-435309 | AO090701000729 |
| Chr1_A_oryzae_RIB40:5705077-5705126 | AO090005000276 |
| Chr8_A_oryzae_RIB40:1414888-1414937 | AO090010000761 |
| Chr3_A_oryzae_RIB40:2050081-2050130 | AO090023000788 |
| Chr2_A_oryzae_RIB40:5728187-5728236 | AO090003001590 |
| Chr1_A_oryzae_RIB40:1670421-1670470 | AO090009000626 |
| Chr1_A_oryzae_RIB40:1670421-1670470 | AO090009000627 |
| Chr6_A_oryzae_RIB40:4089624-4089673 | AO090138000051 |
| Chr5_A_oryzae_RIB40:613124-613173 | AO090701000669 |
| Chr2_A_oryzae_RIB40:5280013-5280062 | AO090003001172 |
| Chr7_A_oryzae_RIB40:1191029-1191078 | AO090011000474 |
| Chr7_A_oryzae_RIB40:1191029-1191078 | AO090011000473 |
| Chr4_A_oryzae_RIB40:1949134-1949183 | AO090012000766 |
| Chr6_A_oryzae_RIB40:3917061-3917110 | AO090038000137 |
| Chr6_A_oryzae_RIB40:3917061-3917110 | AO090010000290 |
| Chr6_A_oryzae_RIB40:3917061-3917110 | AO090026000545 |
| Chr6_A_oryzae_RIB40:3917061-3917110 | AO090010000292 |
| Chr6_A_oryzae_RIB40:2703545-2703594 | AO090038000368 |
| Chr4_A_oryzae_RIB40:2425140-2425189 | AO090012000942 |
| Chr4_A_oryzae_RIB40:82024-82073 | AO090012000038 |
| Chr4_A_oryzae_RIB40:4455983-4456032 | AO090102000035 |
| Chr4_A_oryzae_RIB40:4309985-4310034 | AO090102000091 |
| Chr5_A_oryzae_RIB40:875700-875749 | AO090701000558 |
| Chr4_A_oryzae_RIB40:991030-991079 | AO090012000403 |
| Chr1_A_oryzae_RIB40:5127780-5127829 | AO090005000495 |
| Chr1_A_oryzae_RIB40:5127780-5127829 | AO090005000494 |
| Chr2_A_oryzae_RIB40:4012849-4012898 | AO090003000714 |
| Chr2_A_oryzae_RIB40:4012849-4012898 | AO090003000715 |
| Chr4_A_oryzae_RIB40:3643012-3643061 | AO090102000331 |
| Chr4_A_oryzae_RIB40:3643012-3643061 | AO090102000332 |
| Chr6_A_oryzae_RIB40:3957111-3957160 | AO090138000114 |
| Chr1_A_oryzae_RIB40:5691062-5691111 | AO090005000279 |
| Chr1_A_oryzae_RIB40:1558348-1558397 | AO090009000585 |
| Chr4_A_oryzae_RIB40:1732946-1732995 | AO090012000682 |
| Chr4_A_oryzae_RIB40:1732946-1732995 | AO090012000683 |
| Chr4_A_oryzae_RIB40:4673933-4673982 | AO090166000046 |
| Chr8_A_oryzae_RIB40:820627-820676 | AO090103000172 |
| Chr1_A_oryzae_RIB40:2258871-2258920 | AO090701000434 |
| Chr1_A_oryzae_RIB40:565697-565746 | AO090009000210 |
| Chr1_A_oryzae_RIB40:565697-565746 | AO090009000211 |
| Chr2_A_oryzae_RIB40:4055013-4055062 | AO090003000728 |
| Chr1_A_oryzae_RIB40:4799155-4799204 | AO090005000623 |
| Chr3_A_oryzae_RIB40:2565714-2565763 | AO090701001125 |
| Chr2_A_oryzae_RIB40:720746-720795 | AO090001000290 |
| Chr4_A_oryzae_RIB40:4595126-4595175 | AO090038000137 |
| Chr4_A_oryzae_RIB40:4595126-4595175 | AO090010000290 |
| Chr4_A_oryzae_RIB40:4595126-4595175 | AO090026000545 |
| Chr4_A_oryzae_RIB40:4595126-4595175 | AO090010000292 |
| Chr1_A_oryzae_RIB40:2483705-2483754 | AO090005001488 |
| Chr5_A_oryzae_RIB40:4204622-4204671 | AO090113000064 |
| Chr8_A_oryzae_RIB40:2342776-2342825 | AO090010000377 |
| Chr2_A_oryzae_RIB40:3267371-3267420 | AO090003000441 |
| Chr2_A_oryzae_RIB40:3267371-3267420 | AO090003000442 |
| Chr6_A_oryzae_RIB40:3379475-3379524 | AO090038000113 |
| Chr6_A_oryzae_RIB40:3559022-3559071 | AO090038000040 |
| Chr2_A_oryzae_RIB40:3291241-3291290 | AO090701001089 |
| Chr7_A_oryzae_RIB40:1505755-1505804 | AO090011000592 |
| Chr2_A_oryzae_RIB40:1341166-1341215 | AO090001000516 |
| Chr4_A_oryzae_RIB40:1136513-1136562 | AO090012000454 |
| Chr4_A_oryzae_RIB40:1136513-1136562 | AO090012000452 |
| Chr1_A_oryzae_RIB40:4030231-4030280 | AO090005000895 |
| Chr1_A_oryzae_RIB40:5118315-5118364 | AO090005000498 |
| Chr1_A_oryzae_RIB40:5118315-5118364 | AO090005000499 |
| Chr1_A_oryzae_RIB40:6325157-6325206 | AO090005000042 |
| Chr2_A_oryzae_RIB40:1083293-1083342 | AO090001000431 |
| Chr4_A_oryzae_RIB40:4868269-4868318 | AO090166000119 |
| Chr5_A_oryzae_RIB40:2119077-2119126 | AO090701000090 |
| Chr4_A_oryzae_RIB40:348505-348554 | AO090701000958 |
| Chr5_A_oryzae_RIB40:1230180-1230229 | AO090701000421 |
| Chr3_A_oryzae_RIB40:3796254-3796303 | AO090026000469 |
| Chr4_A_oryzae_RIB40:3697743-3697792 | AO090701001177 |
| Chr4_A_oryzae_RIB40:1470055-1470104 | AO090012000579 |
| Chr2_A_oryzae_RIB40:1754316-1754365 | AO090001000665 |
| Chr4_A_oryzae_RIB40:4063921-4063970 | AO090102000182 |
| Chr6_A_oryzae_RIB40:988954-989003 | AO090020000337 |
| Chr6_A_oryzae_RIB40:988954-989003 | AO090020000336 |
| Chr3_A_oryzae_RIB40:1014368-1014417 | AO090023000395 |
| Chr1_A_oryzae_RIB40:5568172-5568221 | AO090005000337 |
| Chr5_A_oryzae_RIB40:222100-222149 | AO090701000821 |
| Chr2_A_oryzae_RIB40:1479909-1479958 | AO090001000563 |
| Chr3_A_oryzae_RIB40:3746196-3746245 | AO090026000487 |
| Chr4_A_oryzae_RIB40:3299508-3299557 | AO090102000453 |
| Chr6_A_oryzae_RIB40:216673-216722 | AO090020000630 |
| Chr6_A_oryzae_RIB40:951291-951340 | AO090020000352 |
| Chr2_A_oryzae_RIB40:981146-981195 | AO090001000390 |
| Chr1_A_oryzae_RIB40:4330259-4330308 | AO090005000784 |
| Chr1_A_oryzae_RIB40:4330259-4330308 | AO090005000783 |
| Chr3_A_oryzae_RIB40:2311022-2311071 | AO090023000882 |
| Chr2_A_oryzae_RIB40:5139899-5139948 | AO090003001116 |
| Chr1_A_oryzae_RIB40:2935187-2935236 | AO090005001301 |
| Chr1_A_oryzae_RIB40:2935187-2935236 | AO090005001300 |
| Chr3_A_oryzae_RIB40:3682914-3682963 | AO090701000434 |
| Chr6_A_oryzae_RIB40:3476880-3476929 | AO090038000077 |
| Chr8_A_oryzae_RIB40:987015-987064 | AO090103000109 |
| Chr2_A_oryzae_RIB40:5578776-5578825 | AO090003001287 |
| Chr6_A_oryzae_RIB40:2696070-2696119 | AO090038000370 |
| Chr7_A_oryzae_RIB40:2784354-2784403 | AO090206000075 |
| Chr2_A_oryzae_RIB40:3542010-3542059 | AO090003000544 |
| Chr1_A_oryzae_RIB40:2018707-2018756 | AO090005001648 |
| Chr2_A_oryzae_RIB40:5724116-5724165 | AO090003001340 |
| Chr3_A_oryzae_RIB40:3673168-3673217 | AO090026000517 |
| Chr3_A_oryzae_RIB40:3673168-3673217 | AO090026000518 |
| Chr6_A_oryzae_RIB40:713366-713415 | AO090020000435 |
| Chr5_A_oryzae_RIB40:315432-315481 | AO090701000779 |
| Chr2_A_oryzae_RIB40:2656044-2656093 | AO090003000212 |
| Chr2_A_oryzae_RIB40:2656044-2656093 | AO090003000213 |
| Chr5_A_oryzae_RIB40:2102185-2102234 | AO090701000097 |
| Chr5_A_oryzae_RIB40:2102185-2102234 | AO090701000096 |
| Chr6_A_oryzae_RIB40:66119-66168 | AO090020000695 |
| Chr2_A_oryzae_RIB40:1465080-1465129 | AO090001000560 |
| Chr3_A_oryzae_RIB40:2172710-2172759 | AO090023000833 |
| Chr3_A_oryzae_RIB40:2172710-2172759 | AO090023000832 |
| Chr4_A_oryzae_RIB40:4442480-4442529 | AO090102000040 |
| Chr8_A_oryzae_RIB40:2595214-2595263 | AO090038000137 |
| Chr8_A_oryzae_RIB40:2595214-2595263 | AO090010000290 |
| Chr8_A_oryzae_RIB40:2595214-2595263 | AO090026000545 |
| Chr8_A_oryzae_RIB40:2595214-2595263 | AO090010000292 |
| Chr2_A_oryzae_RIB40:4115459-4115508 | AO090003000753 |
| Chr2_A_oryzae_RIB40:4115459-4115508 | AO090003000752 |
| Chr1_A_oryzae_RIB40:768793-768842 | AO090009000289 |
| Chr6_A_oryzae_RIB40:2092811-2092860 | AO090038000577 |
| Chr4_A_oryzae_RIB40:1964319-1964368 | AO090701000434 |
| Chr8_A_oryzae_RIB40:2267118-2267167 | AO090010000414 |
| Chr8_A_oryzae_RIB40:2650590-2650639 | AO090010000270 |
| Chr6_A_oryzae_RIB40:700564-700613 | AO090020000439 |
| Chr7_A_oryzae_RIB40:1721268-1721317 | AO090011000666 |
| Chr1_A_oryzae_RIB40:2666716-2666765 | AO090005001420 |
| Chr2_A_oryzae_RIB40:5083028-5083077 | AO090003001099 |
| Chr1_A_oryzae_RIB40:3691603-3691652 | AO090005001028 |
| Chr8_A_oryzae_RIB40:129153-129202 | AO090103000451 |
| Chr1_A_oryzae_RIB40:5858300-5858349 | AO090005000219 |
| Chr4_A_oryzae_RIB40:3694429-3694478 | AO090701001176 |
| Chr8_A_oryzae_RIB40:719349-719398 | AO090103000218 |
| Chr3_A_oryzae_RIB40:1104570-1104619 | AO090023000433 |
| Chr5_A_oryzae_RIB40:1869052-1869101 | AO090701000177 |
| Chr5_A_oryzae_RIB40:1869052-1869101 | AO090701000178 |
| Chr2_A_oryzae_RIB40:708911-708960 | AO090001000757 |
| Chr3_A_oryzae_RIB40:1878691-1878740 | AO090023000713 |
| Chr1_A_oryzae_RIB40:3988275-3988324 | AO090005000910 |
| Chr6_A_oryzae_RIB40:1808186-1808235 | AO090020000008 |
| Chr7_A_oryzae_RIB40:1039893-1039942 | AO090011000405 |
| Chr4_A_oryzae_RIB40:4468114-4468163 | AO090102000030 |
| Chr8_A_oryzae_RIB40:644217-644266 | AO090103000243 |
| Chr5_A_oryzae_RIB40:4121340-4121389 | AO090113000033 |
| Chr2_A_oryzae_RIB40:6062448-6062497 | AO090003001484 |
| Chr3_A_oryzae_RIB40:325567-325616 | AO090023000128 |
| Chr1_A_oryzae_RIB40:5852156-5852205 | AO090005000221 |
| Chr5_A_oryzae_RIB40:4109826-4109875 | AO090113000027 |
| Chr5_A_oryzae_RIB40:1118813-1118862 | AO090701000469 |
| Chr2_A_oryzae_RIB40:3424099-3424148 | AO090003000495 |
| Chr2_A_oryzae_RIB40:4454004-4454053 | AO090003000869 |
| Chr4_A_oryzae_RIB40:101265-101314 | AO090012000046 |
| Chr8_A_oryzae_RIB40:1580063-1580112 | AO090010000698 |
| Chr2_A_oryzae_RIB40:2527204-2527253 | AO090003000160 |
| Chr3_A_oryzae_RIB40:239201-239250 | AO090023000094 |
| Chr8_A_oryzae_RIB40:397930-397979 | AO090103000341 |
| Chr8_A_oryzae_RIB40:397930-397979 | AO090103000342 |
| Chr1_A_oryzae_RIB40:2642717-2642766 | AO090701001028 |
| Chr5_A_oryzae_RIB40:3324893-3324942 | AO090120000246 |
| Chr1_A_oryzae_RIB40:359685-359734 | AO090009000131 |
| Chr8_A_oryzae_RIB40:2326588-2326637 | AO090010000385 |
| Chr1_A_oryzae_RIB40:4950947-4950996 | AO090005000567 |
| Chr5_A_oryzae_RIB40:195268-195317 | AO090701000829 |
| Chr1_A_oryzae_RIB40:5631047-5631096 | AO090005000306 |
| Chr3_A_oryzae_RIB40:487275-487324 | AO090701000434 |
| Chr3_A_oryzae_RIB40:734663-734712 | AO090023000283 |
| Chr5_A_oryzae_RIB40:3418483-3418532 | AO090120000287 |
| Chr3_A_oryzae_RIB40:1225797-1225846 | AO090023000478 |
| Chr3_A_oryzae_RIB40:809212-809261 | AO090023000318 |
| Chr7_A_oryzae_RIB40:1083437-1083486 | AO090011000970 |
| Chr7_A_oryzae_RIB40:1083437-1083486 | AO090011000425 |
| Chr1_A_oryzae_RIB40:1551547-1551596 | AO090009000582 |
| Chr6_A_oryzae_RIB40:2586562-2586611 | AO090038000404 |
| Chr4_A_oryzae_RIB40:1823681-1823730 | AO090012000717 |
| Chr3_A_oryzae_RIB40:4064041-4064090 | AO090026000368 |
| Chr1_A_oryzae_RIB40:5453010-5453059 | AO090005000382 |
| Chr8_A_oryzae_RIB40:2327258-2327307 | AO090010000386 |
| Chr8_A_oryzae_RIB40:2327258-2327307 | AO090010000385 |
| Chr1_A_oryzae_RIB40:2081517-2081566 | AO090005001626 |
| Chr7_A_oryzae_RIB40:1125189-1125238 | AO090011000444 |
| Chr2_A_oryzae_RIB40:5785607-5785656 | AO090701000925 |
| Chr4_A_oryzae_RIB40:4805818-4805867 | AO090166000089 |
| Chr2_A_oryzae_RIB40:2206912-2206961 | AO090003000043 |
| Chr3_A_oryzae_RIB40:257406-257455 | AO090023000103 |
| Chr5_A_oryzae_RIB40:3608406-3608455 | AO090701000434 |
| Chr7_A_oryzae_RIB40:1703118-1703167 | AO090011000655 |
| Chr3_A_oryzae_RIB40:408049-408098 | AO090023000161 |
| Chr4_A_oryzae_RIB40:1624019-1624068 | AO090012000636 |
| Chr5_A_oryzae_RIB40:3577252-3577301 | AO090120000334 |
| Chr2_A_oryzae_RIB40:963424-963473 | AO090001000382 |
| Chr4_A_oryzae_RIB40:1756072-1756121 | AO090012000690 |
| Chr6_A_oryzae_RIB40:1129935-1129984 | AO090020000276 |
| Chr3_A_oryzae_RIB40:1826407-1826456 | AO090023000692 |
| Chr6_A_oryzae_RIB40:2363928-2363977 | AO090038000487 |
| Chr7_A_oryzae_RIB40:2163295-2163344 | AO090011000846 |
| Chr4_A_oryzae_RIB40:793097-793146 | AO090012000321 |
| Chr6_A_oryzae_RIB40:4075388-4075437 | AO090138000057 |
| Chr3_A_oryzae_RIB40:2500068-2500117 | AO090023000946 |
| Chr3_A_oryzae_RIB40:1489102-1489151 | AO090023000564 |
| Chr4_A_oryzae_RIB40:287830-287879 | AO090012000116 |
| Chr3_A_oryzae_RIB40:3440952-3441001 | AO090026000605 |
| Chr6_A_oryzae_RIB40:794305-794354 | AO090020000413 |
| Chr1_A_oryzae_RIB40:6081485-6081534 | AO090005000135 |
| Chr5_A_oryzae_RIB40:4387413-4387462 | AO090113000143 |
| Chr1_A_oryzae_RIB40:5455255-5455304 | AO090005000381 |
| Chr2_A_oryzae_RIB40:239327-239376 | AO090001000105 |
| Chr2_A_oryzae_RIB40:2563756-2563805 | AO090003000174 |
| Chr4_A_oryzae_RIB40:346449-346498 | AO090012000143 |
| Chr5_A_oryzae_RIB40:826259-826308 | AO090701000576 |
| Chr5_A_oryzae_RIB40:826259-826308 | AO090701000577 |
| Chr7_A_oryzae_RIB40:1128807-1128856 | AO090011000446 |
| Chr7_A_oryzae_RIB40:1128807-1128856 | AO090011000447 |
| Chr8_A_oryzae_RIB40:868252-868301 | AO090103000153 |
| Chr8_A_oryzae_RIB40:2745201-2745250 | AO090010000243 |
| Chr3_A_oryzae_RIB40:1548540-1548589 | AO090023000584 |
| Chr5_A_oryzae_RIB40:1259001-1259050 | AO090701000410 |
| Chr3_A_oryzae_RIB40:3859314-3859363 | AO090026000448 |
| Chr3_A_oryzae_RIB40:3859314-3859363 | AO090026000449 |
| Chr3_A_oryzae_RIB40:448865-448914 | AO090023000179 |
| Chr3_A_oryzae_RIB40:448865-448914 | AO090023000176 |
| Chr8_A_oryzae_RIB40:648000-648049 | AO090103000241 |
| Chr8_A_oryzae_RIB40:648000-648049 | AO090103000242 |
| Chr4_A_oryzae_RIB40:1660944-1660993 | AO090012000653 |
| Chr4_A_oryzae_RIB40:1660944-1660993 | AO090012000652 |
| Chr1_A_oryzae_RIB40:1462359-1462408 | AO090009000554 |
| Chr2_A_oryzae_RIB40:1619405-1619454 | AO090001000618 |
| Chr2_A_oryzae_RIB40:1619405-1619454 | AO090001000619 |
| Chr6_A_oryzae_RIB40:16848-16897 | AO090020000714 |
| Chr6_A_oryzae_RIB40:16848-16897 | AO090020000715 |
| Chr5_A_oryzae_RIB40:3129908-3129957 | AO090120000178 |
| Chr3_A_oryzae_RIB40:4104547-4104596 | AO090026000355 |
| Chr2_A_oryzae_RIB40:2190160-2190209 | AO090003000035 |
| Chr7_A_oryzae_RIB40:1364473-1364522 | AO090011000542 |
| Chr3_A_oryzae_RIB40:2543451-2543500 | AO090023000963 |
| Chr2_A_oryzae_RIB40:1463449-1463498 | AO090001000559 |
| Chr2_A_oryzae_RIB40:1388284-1388333 | AO090001000530 |
| Chr2_A_oryzae_RIB40:1388284-1388333 | AO090001000531 |
| Chr4_A_oryzae_RIB40:2955023-2955072 | AO090102000581 |
| Chr8_A_oryzae_RIB40:2862420-2862469 | AO090010000203 |
| Chr8_A_oryzae_RIB40:2862420-2862469 | AO090010000204 |
| Chr6_A_oryzae_RIB40:983347-983396 | AO090020000340 |
| Chr2_A_oryzae_RIB40:1625578-1625627 | AO090001000622 |
| Chr2_A_oryzae_RIB40:1625578-1625627 | AO090001000621 |
| Chr1_A_oryzae_RIB40:3723148-3723197 | AO090005001012 |
| Chr2_A_oryzae_RIB40:322602-322651 | AO090001000144 |
| Chr2_A_oryzae_RIB40:6122796-6122845 | AO090003001506 |
| Chr4_A_oryzae_RIB40:1210812-1210861 | AO090012000484 |
| Chr7_A_oryzae_RIB40:1320661-1320710 | AO090011000524 |
| Chr7_A_oryzae_RIB40:1707717-1707766 | AO090011000659 |
| Chr3_A_oryzae_RIB40:3180118-3180167 | AO090026000709 |
| Chr1_A_oryzae_RIB40:5678117-5678166 | AO090005000284 |
| Chr2_A_oryzae_RIB40:606465-606514 | AO090001000246 |
| Chr2_A_oryzae_RIB40:3778243-3778292 | AO090003000627 |
| Chr3_A_oryzae_RIB40:3952198-3952247 | AO090026000414 |
| Chr5_A_oryzae_RIB40:1148696-1148745 | AO090701000455 |
| Chr5_A_oryzae_RIB40:2654530-2654579 | AO090124000010 |
| Chr8_A_oryzae_RIB40:1829281-1829330 | AO090010000594 |
| Chr2_A_oryzae_RIB40:26072-26121 | AO090001000009 |
| Chr4_A_oryzae_RIB40:688522-688571 | AO090012000279 |
| Chr5_A_oryzae_RIB40:51332-51381 | AO090701000886 |
| Chr1_A_oryzae_RIB40:720984-721033 | AO090009000270 |
| Chr4_A_oryzae_RIB40:2139722-2139771 | AO090701000966 |
| Chr1_A_oryzae_RIB40:6143915-6143964 | AO090005000114 |
| Chr4_A_oryzae_RIB40:635776-635825 | AO090012000261 |
| Chr6_A_oryzae_RIB40:2087593-2087642 | AO090038000578 |
| Chr7_A_oryzae_RIB40:722466-722515 | AO090011000282 |
| Chr1_A_oryzae_RIB40:53027-53076 | AO090009000014 |
| Chr5_A_oryzae_RIB40:823538-823587 | AO090701000578 |
| Chr3_A_oryzae_RIB40:4575507-4575556 | AO090026000191 |
| Chr2_A_oryzae_RIB40:3928430-3928479 | AO090003000679 |
| Chr1_A_oryzae_RIB40:6500240-6500289 | AO090308000019 |
| Chr4_A_oryzae_RIB40:1943925-1943974 | AO090012000764 |
| Chr5_A_oryzae_RIB40:1631788-1631837 | AO090701000270 |
| Chr6_A_oryzae_RIB40:114334-114383 | AO090020000676 |
| Chr6_A_oryzae_RIB40:3185686-3185735 | AO090701001252 |
| Chr6_A_oryzae_RIB40:3348515-3348564 | AO090038000126 |
| Chr3_A_oryzae_RIB40:349399-349448 | AO090023000136 |
| Chr6_A_oryzae_RIB40:1176585-1176634 | AO090020000258 |
| Chr7_A_oryzae_RIB40:2287717-2287766 | AO090011000893 |
| Chr8_A_oryzae_RIB40:249438-249487 | AO090103000401 |
| Chr1_A_oryzae_RIB40:5794618-5794667 | AO090005000242 |
| Chr2_A_oryzae_RIB40:4222799-4222848 | AO090003000795 |
| Chr4_A_oryzae_RIB40:4572325-4572374 | AO090166000008 |
| Chr3_A_oryzae_RIB40:1173946-1173995 | AO090023000458 |
| Chr5_A_oryzae_RIB40:858564-858613 | AO090701000567 |
| Chr1_A_oryzae_RIB40:4804395-4804444 | AO090005000622 |
| Chr8_A_oryzae_RIB40:2079554-2079603 | AO090010000489 |
| Chr6_A_oryzae_RIB40:922142-922191 | AO090020000365 |
| Chr5_A_oryzae_RIB40:1728676-1728725 | AO090701000231 |
| Chr7_A_oryzae_RIB40:351368-351417 | AO090011000126 |
| Chr7_A_oryzae_RIB40:351368-351417 | AO090011000125 |
| Chr4_A_oryzae_RIB40:3924023-3924072 | AO090102000233 |
| Chr7_A_oryzae_RIB40:950925-950974 | AO090011000370 |
| Chr4_A_oryzae_RIB40:333488-333537 | AO090012000136 |
| Chr2_A_oryzae_RIB40:247760-247809 | AO090001000108 |
| Chr2_A_oryzae_RIB40:247760-247809 | AO090001000109 |
| Chr2_A_oryzae_RIB40:6044481-6044530 | AO090003001478 |
| Chr3_A_oryzae_RIB40:1049245-1049294 | AO090023000409 |
| Chr3_A_oryzae_RIB40:1049245-1049294 | AO090023000410 |
| Chr4_A_oryzae_RIB40:1385117-1385166 | AO090012000544 |
| Chr5_A_oryzae_RIB40:1041354-1041403 | AO090701000500 |
| Chr8_A_oryzae_RIB40:2378415-2378464 | AO090010000366 |
| Chr3_A_oryzae_RIB40:3248756-3248805 | AO090026000682 |
| Chr2_A_oryzae_RIB40:1318348-1318397 | AO090001000511 |
| Chr5_A_oryzae_RIB40:609775-609824 | AO090701000670 |
| Chr5_A_oryzae_RIB40:2501216-2501265 | AO090124000074 |
| Chr5_A_oryzae_RIB40:409929-409978 | AO090701000741 |
| Chr3_A_oryzae_RIB40:2996120-2996169 | AO090026000785 |
| Chr8_A_oryzae_RIB40:1758825-1758874 | AO090010000623 |
| Chr5_A_oryzae_RIB40:790651-790700 | AO090701000594 |
| Chr1_A_oryzae_RIB40:32340-32389 | AO090009000008 |
| Chr2_A_oryzae_RIB40:2587353-2587402 | AO090003000182 |
| Chr2_A_oryzae_RIB40:2587353-2587402 | AO090003000183 |
| Chr7_A_oryzae_RIB40:629081-629130 | AO090011000246 |
| Chr8_A_oryzae_RIB40:524333-524382 | AO090103000291 |
| Chr1_A_oryzae_RIB40:5898255-5898304 | AO090005000203 |
| Chr2_A_oryzae_RIB40:5080855-5080904 | AO090003001097 |
| Chr2_A_oryzae_RIB40:5080855-5080904 | AO090003001098 |
| Chr3_A_oryzae_RIB40:5016039-5016088 | AO090026000039 |
| Chr6_A_oryzae_RIB40:40822-40871 | AO090020000704 |
| Chr1_A_oryzae_RIB40:2705728-2705777 | AO090005001404 |
| Chr1_A_oryzae_RIB40:4346553-4346602 | AO090005000778 |
| Chr4_A_oryzae_RIB40:146173-146222 | AO090012000064 |
| Chr4_A_oryzae_RIB40:3308635-3308684 | AO090102000449 |
| Chr4_A_oryzae_RIB40:3308635-3308684 | AO090102000448 |
| Chr4_A_oryzae_RIB40:3308635-3308684 | AO090701001174 |
| Chr3_A_oryzae_RIB40:3692986-3693035 | AO090026000508 |
| Chr7_A_oryzae_RIB40:17742-17791 | AO090011000009 |
| Chr7_A_oryzae_RIB40:317410-317459 | AO090011000113 |
| Chr4_A_oryzae_RIB40:4608105-4608154 | AO090166000019 |
| Chr2_A_oryzae_RIB40:4139500-4139549 | AO090003000761 |
| Chr3_A_oryzae_RIB40:2274376-2274425 | AO090023000870 |
| Chr3_A_oryzae_RIB40:2274376-2274425 | AO090023000871 |
| Chr1_A_oryzae_RIB40:3879218-3879267 | AO090005000955 |
| Chr1_A_oryzae_RIB40:3879218-3879267 | AO090005000956 |
| Chr1_A_oryzae_RIB40:5769773-5769822 | AO090005000250 |
| Chr2_A_oryzae_RIB40:3580664-3580713 | AO090003000558 |
| Chr2_A_oryzae_RIB40:5526560-5526609 | AO090003001265 |
| Chr3_A_oryzae_RIB40:748914-748963 | AO090023000291 |
| Chr8_A_oryzae_RIB40:765507-765556 | AO090103000202 |
| Chr2_A_oryzae_RIB40:3545481-3545530 | AO090003000545 |
| Chr2_A_oryzae_RIB40:2818394-2818443 | AO090003000270 |
| Chr1_A_oryzae_RIB40:1064217-1064266 | AO090009000400 |
| Chr3_A_oryzae_RIB40:4997084-4997133 | AO090026000048 |
| Chr8_A_oryzae_RIB40:281761-281810 | AO090103000384 |
| Chr8_A_oryzae_RIB40:281761-281810 | AO090103000385 |
| Chr1_A_oryzae_RIB40:2538682-2538731 | AO090005001471 |
| Chr8_A_oryzae_RIB40:2425463-2425512 | AO090010000349 |
| Chr2_A_oryzae_RIB40:718721-718770 | AO090001000289 |
| Chr2_A_oryzae_RIB40:4853897-4853946 | AO090003001021 |
| Chr2_A_oryzae_RIB40:4853897-4853946 | AO090003001020 |
| Chr5_A_oryzae_RIB40:2351670-2351719 | AO090701000002 |
| Chr6_A_oryzae_RIB40:4063404-4063453 | AO090138000065 |
| Chr8_A_oryzae_RIB40:2524265-2524314 | AO090010000304 |
| Chr2_A_oryzae_RIB40:2927566-2927615 | AO090003000307 |
| Chr2_A_oryzae_RIB40:1423840-1423889 | AO090001000544 |
| Chr1_A_oryzae_RIB40:2831227-2831276 | AO090005001345 |
| Chr5_A_oryzae_RIB40:3605151-3605200 | AO090120000346 |
| Chr5_A_oryzae_RIB40:1437989-1438038 | AO090701001195 |
| Chr1_A_oryzae_RIB40:2883673-2883722 | AO090005001321 |
| Chr4_A_oryzae_RIB40:3264804-3264853 | AO090102000465 |
| Chr6_A_oryzae_RIB40:435269-435318 | AO090701001222 |
| Chr5_A_oryzae_RIB40:2303092-2303141 | AO090701000021 |
| Chr3_A_oryzae_RIB40:318249-318298 | AO090023000126 |
| Chr1_A_oryzae_RIB40:1869989-1870038 | AO090009000701 |
| Chr5_A_oryzae_RIB40:2709263-2709312 | AO090120000007 |
| Chr2_A_oryzae_RIB40:431476-431525 | AO090001000188 |
| Chr3_A_oryzae_RIB40:4998032-4998081 | AO090026000047 |
| Chr3_A_oryzae_RIB40:4694223-4694272 | AO090026000150 |
| Chr3_A_oryzae_RIB40:1426385-1426434 | AO090023000536 |
| Chr4_A_oryzae_RIB40:2226148-2226197 | AO090012000863 |
| Chr4_A_oryzae_RIB40:2226148-2226197 | AO090012000862 |
| Chr5_A_oryzae_RIB40:1510317-1510366 | AO090701000316 |
| Chr3_A_oryzae_RIB40:4610500-4610549 | AO090026000180 |
| Chr3_A_oryzae_RIB40:4610500-4610549 | AO090026000181 |
| Chr1_A_oryzae_RIB40:2201877-2201926 | AO090005001578 |
| Chr7_A_oryzae_RIB40:79134-79183 | AO090011000030 |
| Chr6_A_oryzae_RIB40:3576777-3576826 | AO090038000032 |
| Chr2_A_oryzae_RIB40:2757593-2757642 | AO090003000251 |
| Chr1_A_oryzae_RIB40:2980348-2980397 | AO090038000137 |
| Chr1_A_oryzae_RIB40:2980348-2980397 | AO090010000290 |
| Chr1_A_oryzae_RIB40:2980348-2980397 | AO090026000545 |
| Chr1_A_oryzae_RIB40:2980348-2980397 | AO090010000292 |
| Chr2_A_oryzae_RIB40:608509-608558 | AO090001000248 |
| Chr6_A_oryzae_RIB40:398435-398484 | AO090020000561 |
| Chr3_A_oryzae_RIB40:278537-278586 | AO090023000109 |
| Chr3_A_oryzae_RIB40:278537-278586 | AO090023000108 |
| Chr4_A_oryzae_RIB40:3708938-3708987 | AO090102000307 |
| Chr6_A_oryzae_RIB40:1660278-1660327 | AO090020000058 |
| Chr6_A_oryzae_RIB40:1660278-1660327 | AO090020000060 |
| Chr5_A_oryzae_RIB40:4074695-4074744 | AO090113000011 |
| Chr1_A_oryzae_RIB40:374877-374926 | AO090009000140 |
| Chr2_A_oryzae_RIB40:545292-545341 | AO090001000225 |
| Chr3_A_oryzae_RIB40:4651402-4651451 | AO090026000165 |
| Chr2_A_oryzae_RIB40:6029857-6029906 | AO090003001472 |
| Chr2_A_oryzae_RIB40:6263218-6263267 | AO090003001558 |
| Chr5_A_oryzae_RIB40:2914627-2914676 | AO090120000095 |
| Chr2_A_oryzae_RIB40:5267412-5267461 | AO090003001164 |
| Chr1_A_oryzae_RIB40:1453565-1453614 | AO090009000551 |
| Chr6_A_oryzae_RIB40:2288883-2288932 | AO090038000513 |
| Chr1_A_oryzae_RIB40:1373125-1373174 | AO090009000516 |
| Chr1_A_oryzae_RIB40:1373125-1373174 | AO090009000517 |
| Chr5_A_oryzae_RIB40:663813-663862 | AO090701000645 |
| Chr1_A_oryzae_RIB40:827893-827942 | AO090009000310 |
| Chr4_A_oryzae_RIB40:2424423-2424472 | AO090012000941 |
| Chr6_A_oryzae_RIB40:331398-331447 | AO090020000590 |
| Chr2_A_oryzae_RIB40:780515-780564 | AO090001000305 |
| Chr3_A_oryzae_RIB40:1769113-1769162 | AO090023000672 |
| Chr3_A_oryzae_RIB40:1769113-1769162 | AO090023000673 |
| Chr5_A_oryzae_RIB40:716588-716637 | AO090701000625 |
| Chr8_A_oryzae_RIB40:331502-331551 | AO090103000361 |
| Chr4_A_oryzae_RIB40:2534223-2534272 | AO090012000978 |
| Chr1_A_oryzae_RIB40:1834876-1834925 | AO090009000684 |
| Chr1_A_oryzae_RIB40:1834876-1834925 | AO090009000685 |
| Chr6_A_oryzae_RIB40:1296870-1296919 | AO090020000209 |
| Chr1_A_oryzae_RIB40:4695328-4695377 | AO090005000656 |
| Chr1_A_oryzae_RIB40:4695328-4695377 | AO090005000657 |
| Chr2_A_oryzae_RIB40:4704845-4704894 | AO090003000963 |
| Chr7_A_oryzae_RIB40:947599-947648 | AO090011000368 |
| Chr7_A_oryzae_RIB40:947599-947648 | AO090011000369 |
| Chr3_A_oryzae_RIB40:3589882-3589931 | AO090038000137 |
| Chr3_A_oryzae_RIB40:3589882-3589931 | AO090010000290 |
| Chr3_A_oryzae_RIB40:3589882-3589931 | AO090026000545 |
| Chr3_A_oryzae_RIB40:3589882-3589931 | AO090010000292 |
| Chr3_A_oryzae_RIB40:1372911-1372960 | AO090023000523 |
| Chr4_A_oryzae_RIB40:145540-145589 | AO090012000063 |
| Chr5_A_oryzae_RIB40:3184730-3184779 | AO090023000942 |
| Chr5_A_oryzae_RIB40:3184730-3184779 | AO090120000198 |
| Chr5_A_oryzae_RIB40:3184730-3184779 | AO090023000974 |
| Chr5_A_oryzae_RIB40:3184730-3184779 | AO090701001123 |
| Chr8_A_oryzae_RIB40:1828771-1828820 | AO090010000594 |
| Chr4_A_oryzae_RIB40:4312363-4312412 | AO090102000090 |
| Chr5_A_oryzae_RIB40:4422227-4422276 | AO090113000160 |
| Chr3_A_oryzae_RIB40:754138-754187 | AO090023000296 |
| Chr6_A_oryzae_RIB40:1017581-1017630 | AO090020000324 |
| Chr4_A_oryzae_RIB40:4300391-4300440 | AO090102000096 |
| Chr5_A_oryzae_RIB40:962669-962718 | AO090701000527 |
| Chr8_A_oryzae_RIB40:133689-133738 | AO090103000450 |
| Chr8_A_oryzae_RIB40:133689-133738 | AO090103000449 |
| Chr1_A_oryzae_RIB40:666812-666861 | AO090009000252 |
| Chr1_A_oryzae_RIB40:814771-814820 | AO090701001018 |
| Chr2_A_oryzae_RIB40:2173157-2173206 | AO090003000028 |
| Chr5_A_oryzae_RIB40:3198434-3198483 | AO090120000204 |
| Chr6_A_oryzae_RIB40:3279442-3279491 | AO090038000155 |
| Chr7_A_oryzae_RIB40:371961-372010 | AO090011000134 |
| Chr7_A_oryzae_RIB40:785194-785243 | AO090011000310 |
| Chr7_A_oryzae_RIB40:785194-785243 | AO090011000309 |
| Chr7_A_oryzae_RIB40:1333759-1333808 | AO090026000084 |
| Chr7_A_oryzae_RIB40:1333759-1333808 | AO090003000903 |
| Chr8_A_oryzae_RIB40:539574-539623 | AO090103000284 |
| Chr8_A_oryzae_RIB40:3033294-3033343 | AO090010000140 |
| Chr1_A_oryzae_RIB40:1093141-1093190 | AO090009000414 |
| Chr2_A_oryzae_RIB40:2339681-2339730 | AO090003000095 |
| Chr5_A_oryzae_RIB40:4415842-4415891 | AO090113000157 |
| Chr2_A_oryzae_RIB40:951387-951436 | AO090001000378 |
| Chr2_A_oryzae_RIB40:221488-221537 | AO090001000099 |
| Chr3_A_oryzae_RIB40:4761971-4762020 | AO090026000126 |
| Chr4_A_oryzae_RIB40:3474309-3474358 | AO090102000386 |
| Chr4_A_oryzae_RIB40:4749361-4749410 | AO090166000070 |
| Chr5_A_oryzae_RIB40:165998-166047 | AO090701000838 |
| Chr5_A_oryzae_RIB40:2623133-2623182 | AO090124000023 |
| Chr6_A_oryzae_RIB40:2414397-2414446 | AO090701001245 |
| Chr8_A_oryzae_RIB40:2519266-2519315 | AO090010000308 |
| Chr4_A_oryzae_RIB40:731221-731270 | AO090012000296 |
| Chr2_A_oryzae_RIB40:666316-666365 | AO090001000267 |
| Chr7_A_oryzae_RIB40:2279815-2279864 | AO090011000892 |
| Chr7_A_oryzae_RIB40:819140-819189 | AO090011000324 |
| Chr3_A_oryzae_RIB40:2358819-2358868 | AO090023000901 |
| Chr3_A_oryzae_RIB40:844965-845014 | AO090023000331 |
| Chr1_A_oryzae_RIB40:5915148-5915197 | AO090005000194 |
| Chr3_A_oryzae_RIB40:2572537-2572586 | AO090023000942 |
| Chr3_A_oryzae_RIB40:2572537-2572586 | AO090120000198 |
| Chr3_A_oryzae_RIB40:2572537-2572586 | AO090023000974 |
| Chr3_A_oryzae_RIB40:2572537-2572586 | AO090701001123 |
| Chr5_A_oryzae_RIB40:2770098-2770147 | AO090120000036 |
| Chr5_A_oryzae_RIB40:2770098-2770147 | AO090120000034 |
| Chr6_A_oryzae_RIB40:1761863-1761912 | AO090020000026 |
| Chr6_A_oryzae_RIB40:1761863-1761912 | AO090020000025 |
| Chr6_A_oryzae_RIB40:3007755-3007804 | AO090038000251 |
| Chr6_A_oryzae_RIB40:3007755-3007804 | AO090701001251 |
| Chr6_A_oryzae_RIB40:3007755-3007804 | AO090038000250 |
| Chr3_A_oryzae_RIB40:641488-641537 | AO090023000249 |
| Chr6_A_oryzae_RIB40:2690035-2690084 | AO090038000376 |
| Chr5_A_oryzae_RIB40:1268338-1268387 | AO090701000406 |
| Chr1_A_oryzae_RIB40:2753549-2753598 | AO090005001383 |
| Chr1_A_oryzae_RIB40:2753549-2753598 | AO090005001384 |
| Chr1_A_oryzae_RIB40:1682748-1682797 | AO090009000630 |
| Chr2_A_oryzae_RIB40:978686-978735 | AO090001000387 |
| Chr8_A_oryzae_RIB40:152507-152556 | AO090103000443 |
| Chr5_A_oryzae_RIB40:1864524-1864573 | AO090701000180 |
| Chr3_A_oryzae_RIB40:622772-622821 | AO090023000245 |
| Chr3_A_oryzae_RIB40:1889687-1889736 | AO090023000717 |
| Chr1_A_oryzae_RIB40:5158226-5158275 | AO090005000481 |
| Chr1_A_oryzae_RIB40:5158226-5158275 | AO090005000482 |
| Chr1_A_oryzae_RIB40:4601942-4601991 | AO090005000691 |
| Chr5_A_oryzae_RIB40:221350-221399 | AO090701000820 |
| Chr2_A_oryzae_RIB40:3339214-3339263 | AO090003000465 |
| Chr4_A_oryzae_RIB40:3623757-3623806 | AO090102000338 |
| Chr4_A_oryzae_RIB40:3623757-3623806 | AO090102000337 |
| Chr3_A_oryzae_RIB40:368130-368179 | AO090023000143 |
| Chr3_A_oryzae_RIB40:368130-368179 | AO090023000144 |
| Chr4_A_oryzae_RIB40:1569619-1569668 | AO090012000614 |
| Chr4_A_oryzae_RIB40:1569619-1569668 | AO090012000615 |
| Chr5_A_oryzae_RIB40:2319569-2319618 | AO090701000014 |
| Chr5_A_oryzae_RIB40:2964267-2964316 | AO090120000114 |
| Chr5_A_oryzae_RIB40:4483164-4483213 | AO090113000186 |
| Chr7_A_oryzae_RIB40:474078-474127 | AO090011000179 |
| Chr7_A_oryzae_RIB40:2151127-2151176 | AO090011000841 |
| Chr6_A_oryzae_RIB40:2131596-2131645 | AO090038000563 |
| Chr8_A_oryzae_RIB40:1730014-1730063 | AO090010000635 |
| Chr5_A_oryzae_RIB40:1645004-1645053 | AO090701000265 |
| Chr3_A_oryzae_RIB40:152261-152310 | AO090023000056 |
| Chr5_A_oryzae_RIB40:2609099-2609148 | AO090124000030 |
| Chr5_A_oryzae_RIB40:2609099-2609148 | AO090124000029 |
| Chr6_A_oryzae_RIB40:180465-180514 | AO090701001220 |
| Chr6_A_oryzae_RIB40:1511157-1511206 | AO090020000119 |
| Chr7_A_oryzae_RIB40:728102-728151 | AO090011000285 |
| Chr8_A_oryzae_RIB40:2602136-2602185 | AO090038000137 |
| Chr8_A_oryzae_RIB40:2602136-2602185 | AO090010000290 |
| Chr8_A_oryzae_RIB40:2602136-2602185 | AO090026000545 |
| Chr8_A_oryzae_RIB40:2602136-2602185 | AO090010000292 |
| Chr1_A_oryzae_RIB40:1160518-1160567 | AO090009000445 |
| Chr3_A_oryzae_RIB40:924207-924256 | AO090023000362 |
| Chr3_A_oryzae_RIB40:3230901-3230950 | AO090026000688 |
| Chr2_A_oryzae_RIB40:1942557-1942606 | AO090001000727 |
| Chr2_A_oryzae_RIB40:1942557-1942606 | AO090001000728 |
| Chr1_A_oryzae_RIB40:3381288-3381337 | AO090701001031 |
| Chr1_A_oryzae_RIB40:5960860-5960909 | AO090005000173 |
| Chr2_A_oryzae_RIB40:1700187-1700236 | AO090026000084 |
| Chr2_A_oryzae_RIB40:1700187-1700236 | AO090003000903 |
| Chr2_A_oryzae_RIB40:5978379-5978428 | AO090003001448 |
| Chr3_A_oryzae_RIB40:4682754-4682803 | AO090026000155 |
| Chr3_A_oryzae_RIB40:4856708-4856757 | AO090026000093 |
| Chr4_A_oryzae_RIB40:4358690-4358739 | AO090102000072 |
| Chr7_A_oryzae_RIB40:489636-489685 | AO090011000187 |
| Chr7_A_oryzae_RIB40:489636-489685 | AO090011000186 |
| Chr8_A_oryzae_RIB40:310376-310425 | AO090103000372 |
| Chr7_A_oryzae_RIB40:795840-795889 | AO090011000315 |
| Chr3_A_oryzae_RIB40:1038010-1038059 | AO090023000403 |
| Chr1_A_oryzae_RIB40:5251323-5251372 | AO090005000451 |
| Chr1_A_oryzae_RIB40:5251323-5251372 | AO090005000450 |
| Chr3_A_oryzae_RIB40:3057635-3057684 | AO090026000758 |
| Chr3_A_oryzae_RIB40:1827988-1828037 | AO090023000694 |
| Chr4_A_oryzae_RIB40:2015318-2015367 | AO090012000783 |
| Chr4_A_oryzae_RIB40:2015318-2015367 | AO090012000782 |
| Chr3_A_oryzae_RIB40:4939341-4939390 | AO090026000071 |
| Chr6_A_oryzae_RIB40:3729193-3729242 | AO090138000192 |
| Chr3_A_oryzae_RIB40:418213-418262 | AO090023000165 |
| Chr2_A_oryzae_RIB40:5127393-5127442 | AO090003001114 |
| Chr1_A_oryzae_RIB40:3978630-3978679 | AO090005000917 |
| Chr1_A_oryzae_RIB40:2283267-2283316 | AO090005001552 |
| Chr1_A_oryzae_RIB40:4422067-4422116 | AO090005000752 |
| Chr2_A_oryzae_RIB40:109144-109193 | AO090001000051 |
| Chr3_A_oryzae_RIB40:458914-458963 | AO090023000184 |
| Chr3_A_oryzae_RIB40:1180033-1180082 | AO090023000460 |
| Chr5_A_oryzae_RIB40:2895857-2895906 | AO090120000087 |
| Chr7_A_oryzae_RIB40:2469639-2469688 | AO090011000942 |
| Chr2_A_oryzae_RIB40:4480205-4480254 | AO090003000879 |
| Chr7_A_oryzae_RIB40:327177-327226 | AO090011000115 |
| Chr1_A_oryzae_RIB40:4257590-4257639 | AO090005000806 |
| Chr2_A_oryzae_RIB40:3882442-3882491 | AO090003000665 |
| Chr3_A_oryzae_RIB40:1166811-1166860 | AO090023000455 |
| Chr4_A_oryzae_RIB40:1226193-1226242 | AO090012000490 |
| Chr1_A_oryzae_RIB40:3889530-3889579 | AO090005000951 |
| Chr7_A_oryzae_RIB40:2397723-2397772 | AO090011000918 |
| Chr1_A_oryzae_RIB40:4019296-4019345 | AO090005000899 |
| Chr2_A_oryzae_RIB40:2856369-2856418 | AO090003000282 |
| Chr4_A_oryzae_RIB40:2872252-2872301 | AO090102000614 |
| Chr2_A_oryzae_RIB40:4392947-4392996 | AO090701001101 |
| Chr2_A_oryzae_RIB40:2137469-2137518 | AO090003000017 |
| Chr1_A_oryzae_RIB40:1699455-1699504 | AO090009000636 |
| Chr1_A_oryzae_RIB40:1280688-1280737 | AO090009000484 |
| Chr4_A_oryzae_RIB40:3100168-3100217 | AO090102000523 |
| Chr3_A_oryzae_RIB40:3885303-3885352 | AO090026000437 |
| Chr1_A_oryzae_RIB40:1419316-1419365 | AO090009000532 |
| Chr1_A_oryzae_RIB40:2459392-2459441 | AO090005001497 |
| Chr2_A_oryzae_RIB40:2133771-2133820 | AO090003000015 |
| Chr2_A_oryzae_RIB40:2502967-2503016 | AO090003000150 |
| Chr3_A_oryzae_RIB40:1431384-1431433 | AO090023000537 |
| Chr4_A_oryzae_RIB40:105147-105196 | AO090012000049 |
| Chr6_A_oryzae_RIB40:117185-117234 | AO090026000084 |
| Chr6_A_oryzae_RIB40:117185-117234 | AO090003000903 |
| Chr1_A_oryzae_RIB40:3145414-3145463 | AO090005001236 |
| Chr2_A_oryzae_RIB40:4930327-4930376 | AO090003001055 |
| Chr2_A_oryzae_RIB40:1438306-1438355 | AO090001000551 |
| Chr3_A_oryzae_RIB40:1259718-1259767 | AO090023000487 |
| Chr2_A_oryzae_RIB40:4585456-4585505 | AO090003000918 |
| Chr3_A_oryzae_RIB40:914871-914920 | AO090023000358 |
| Chr3_A_oryzae_RIB40:914871-914920 | AO090023000357 |
| Chr2_A_oryzae_RIB40:5979217-5979266 | AO090003001448 |
| Chr6_A_oryzae_RIB40:384804-384853 | AO090020000564 |
| Chr2_A_oryzae_RIB40:5705644-5705693 | AO090003001332 |
| Chr6_A_oryzae_RIB40:2533350-2533399 | AO090038000426 |
| Chr2_A_oryzae_RIB40:2200184-2200233 | AO090003000041 |
| Chr6_A_oryzae_RIB40:985005-985054 | AO090020000338 |
| Chr6_A_oryzae_RIB40:985005-985054 | AO090020000339 |
| Chr4_A_oryzae_RIB40:2598577-2598626 | AO090012001004 |
| Chr4_A_oryzae_RIB40:2598577-2598626 | AO090012001005 |
| Chr1_A_oryzae_RIB40:6204028-6204077 | AO090005000089 |
| Chr1_A_oryzae_RIB40:158784-158833 | AO090009000055 |
| Chr1_A_oryzae_RIB40:5064474-5064523 | AO090005000519 |
| Chr2_A_oryzae_RIB40:3651342-3651391 | AO090003000585 |
| Chr3_A_oryzae_RIB40:507948-507997 | AO090026000084 |
| Chr3_A_oryzae_RIB40:507948-507997 | AO090003000903 |
| Chr3_A_oryzae_RIB40:2254782-2254831 | AO090023000865 |
| Chr6_A_oryzae_RIB40:1294801-1294850 | AO090020000210 |
| Chr8_A_oryzae_RIB40:934455-934504 | AO090103000126 |
| Chr8_A_oryzae_RIB40:1886422-1886471 | AO090010000571 |
| Chr8_A_oryzae_RIB40:2258722-2258771 | AO090010000418 |
| Chr8_A_oryzae_RIB40:3271130-3271179 | AO090010000050 |
| Chr1_A_oryzae_RIB40:2281337-2281386 | AO090005001553 |
| Chr2_A_oryzae_RIB40:150617-150666 | AO090001000067 |
| Chr2_A_oryzae_RIB40:2366022-2366071 | AO090003000102 |
| Chr2_A_oryzae_RIB40:2356515-2356564 | AO090003000100 |
| Chr2_A_oryzae_RIB40:2356515-2356564 | AO090003000101 |
| Chr5_A_oryzae_RIB40:1530509-1530558 | AO090701000310 |
| Chr1_A_oryzae_RIB40:4882720-4882769 | AO090005000589 |
| Chr2_A_oryzae_RIB40:3413658-3413707 | AO090003000492 |
| Chr2_A_oryzae_RIB40:4229725-4229774 | AO090003000798 |
| Chr1_A_oryzae_RIB40:3718725-3718774 | AO090005001015 |
| Chr1_A_oryzae_RIB40:3718725-3718774 | AO090005001014 |
| Chr4_A_oryzae_RIB40:188900-188949 | AO090012000082 |
| Chr5_A_oryzae_RIB40:1135660-1135709 | AO090701000462 |
| Chr8_A_oryzae_RIB40:776490-776539 | AO090103000195 |
| Chr8_A_oryzae_RIB40:776490-776539 | AO090103000196 |
| Chr1_A_oryzae_RIB40:42556-42605 | AO090009000011 |
| Chr1_A_oryzae_RIB40:42556-42605 | AO090009000010 |
| Chr2_A_oryzae_RIB40:2467628-2467677 | AO090003000139 |
| Chr7_A_oryzae_RIB40:2265120-2265169 | AO090701001277 |
| Chr3_A_oryzae_RIB40:501195-501244 | AO090023000201 |
| Chr8_A_oryzae_RIB40:3224313-3224362 | AO090010000068 |
| Chr3_A_oryzae_RIB40:4569869-4569918 | AO090026000193 |
| Chr2_A_oryzae_RIB40:1649079-1649128 | AO090001000629 |
| Chr5_A_oryzae_RIB40:1473527-1473576 | AO090701000329 |
| Chr6_A_oryzae_RIB40:1158209-1158258 | AO090020000265 |
| Chr4_A_oryzae_RIB40:2991577-2991626 | AO090102000565 |
| Chr1_A_oryzae_RIB40:3205073-3205122 | AO090026000084 |
| Chr1_A_oryzae_RIB40:3205073-3205122 | AO090003000903 |
| Chr2_A_oryzae_RIB40:1828930-1828979 | AO090001000692 |
| Chr2_A_oryzae_RIB40:2227907-2227956 | AO090026000084 |
| Chr2_A_oryzae_RIB40:2227907-2227956 | AO090003000903 |
| Chr2_A_oryzae_RIB40:4853290-4853339 | AO090003001021 |
| Chr3_A_oryzae_RIB40:2807763-2807812 | AO090026000844 |
| Chr3_A_oryzae_RIB40:3341826-3341875 | AO090026000645 |
| Chr4_A_oryzae_RIB40:2128634-2128683 | AO090012000826 |
| Chr6_A_oryzae_RIB40:3961817-3961866 | AO090138000111 |
| Chr4_A_oryzae_RIB40:248846-248895 | AO090012000102 |
| Chr1_A_oryzae_RIB40:4883585-4883634 | AO090005000589 |
| Chr6_A_oryzae_RIB40:3603838-3603887 | AO090038000022 |
| Chr8_A_oryzae_RIB40:38576-38625 | AO090103000495 |
| Chr8_A_oryzae_RIB40:38576-38625 | AO090103000494 |
| Chr6_A_oryzae_RIB40:1238570-1238619 | AO090020000232 |
| Chr8_A_oryzae_RIB40:2809366-2809415 | AO090010000221 |
| Chr8_A_oryzae_RIB40:90549-90598 | AO090701000997 |
| Chr8_A_oryzae_RIB40:2339058-2339107 | AO090010000379 |
| Chr8_A_oryzae_RIB40:2339058-2339107 | AO090010000380 |
| Chr5_A_oryzae_RIB40:2728350-2728399 | AO090120000018 |
| Chr7_A_oryzae_RIB40:421792-421841 | AO090011000155 |
| Chr7_A_oryzae_RIB40:421792-421841 | AO090011000156 |
| Chr2_A_oryzae_RIB40:5525920-5525969 | AO090003001263 |
| Chr4_A_oryzae_RIB40:27530-27579 | AO090012000011 |
| Chr4_A_oryzae_RIB40:1880302-1880351 | AO090701001160 |
| Chr5_A_oryzae_RIB40:2693523-2693572 | AO090120000001 |
| Chr5_A_oryzae_RIB40:4248447-4248496 | AO090113000082 |
| Chr1_A_oryzae_RIB40:851230-851279 | AO090009000320 |
| Chr1_A_oryzae_RIB40:5189698-5189747 | AO090005000472 |
| Chr2_A_oryzae_RIB40:5887500-5887549 | AO090003001407 |
| Chr8_A_oryzae_RIB40:874723-874772 | AO090103000152 |
| Chr2_A_oryzae_RIB40:3188058-3188107 | AO090003000415 |
| Chr2_A_oryzae_RIB40:5959860-5959909 | AO090003001436 |
| Chr2_A_oryzae_RIB40:5959860-5959909 | AO090701000926 |
| Chr2_A_oryzae_RIB40:4374991-4375040 | AO090003000842 |
| Chr7_A_oryzae_RIB40:733538-733587 | AO090011000286 |
| Chr1_A_oryzae_RIB40:4686261-4686310 | AO090005000660 |
| Chr6_A_oryzae_RIB40:997817-997866 | AO090020000331 |
| Chr2_A_oryzae_RIB40:577279-577328 | AO090001000237 |
| Chr1_A_oryzae_RIB40:22476-22525 | AO090009000006 |
| Chr1_A_oryzae_RIB40:2109780-2109829 | AO090005001613 |
| Chr3_A_oryzae_RIB40:1134566-1134615 | AO090023000444 |
| Chr3_A_oryzae_RIB40:1134566-1134615 | AO090023000445 |
| Chr3_A_oryzae_RIB40:4269856-4269905 | AO090026000299 |
| Chr4_A_oryzae_RIB40:1772327-1772376 | AO090012000699 |
| Chr4_A_oryzae_RIB40:3079890-3079939 | AO090102000533 |
| Chr5_A_oryzae_RIB40:983286-983335 | AO090701000521 |
| Chr7_A_oryzae_RIB40:1279338-1279387 | AO090011000510 |
| Chr8_A_oryzae_RIB40:802269-802318 | AO090103000180 |
| Chr8_A_oryzae_RIB40:915926-915975 | AO090103000133 |
| mito_A_oryzae_RIB40:8470-8519 | AO090002000050 |
| Chr5_A_oryzae_RIB40:1255655-1255704 | AO090701000412 |
| Chr3_A_oryzae_RIB40:2508993-2509042 | AO090023000949 |
| Chr6_A_oryzae_RIB40:72094-72143 | AO090020000693 |
| Chr1_A_oryzae_RIB40:1252027-1252076 | AO090009000476 |
| Chr1_A_oryzae_RIB40:1252027-1252076 | AO090009000475 |
| Chr6_A_oryzae_RIB40:2665586-2665635 | AO090038000386 |
| Chr7_A_oryzae_RIB40:1464231-1464280 | AO090011000577 |
| Chr4_A_oryzae_RIB40:1158596-1158645 | AO090012000464 |
| Chr2_A_oryzae_RIB40:1967949-1967998 | AO090001000737 |
| Chr6_A_oryzae_RIB40:3431167-3431216 | AO090038000090 |
| Chr1_A_oryzae_RIB40:4934591-4934640 | AO090005000575 |
| Chr1_A_oryzae_RIB40:4934591-4934640 | AO090005000574 |
| Chr4_A_oryzae_RIB40:4127066-4127115 | AO090701000989 |
| Chr3_A_oryzae_RIB40:1877299-1877348 | AO090023000711 |
| Chr2_A_oryzae_RIB40:6114599-6114648 | AO090003001501 |
| Chr4_A_oryzae_RIB40:2275841-2275890 | AO090012000876 |
| Chr3_A_oryzae_RIB40:3197079-3197128 | AO090026000704 |
| Chr4_A_oryzae_RIB40:4861340-4861389 | AO090166000116 |
| Chr8_A_oryzae_RIB40:2003660-2003709 | AO090010000520 |
| Chr6_A_oryzae_RIB40:809105-809154 | AO090020000407 |
| Chr6_A_oryzae_RIB40:809105-809154 | AO090020000406 |
| Chr2_A_oryzae_RIB40:34040-34089 | AO090001000014 |
| Chr8_A_oryzae_RIB40:187805-187854 | AO090103000427 |
| Chr8_A_oryzae_RIB40:187805-187854 | AO090103000428 |
| Chr5_A_oryzae_RIB40:2882363-2882412 | AO090120000085 |
| Chr4_A_oryzae_RIB40:2047362-2047411 | AO090012000796 |
| Chr1_A_oryzae_RIB40:1591945-1591994 | AO090009000597 |
| Chr7_A_oryzae_RIB40:2249003-2249052 | AO090011000878 |
| Chr3_A_oryzae_RIB40:108252-108301 | AO090023000042 |
| Chr2_A_oryzae_RIB40:2754575-2754624 | AO090003000250 |
| Chr8_A_oryzae_RIB40:3184109-3184158 | AO090010000087 |
| Chr7_A_oryzae_RIB40:430879-430928 | AO090011000160 |
| Chr2_A_oryzae_RIB40:892122-892171 | AO090001000357 |
| Chr2_A_oryzae_RIB40:892122-892171 | AO090001000356 |
| Chr2_A_oryzae_RIB40:1511626-1511675 | AO090001000575 |
| Chr5_A_oryzae_RIB40:2734108-2734157 | AO090120000021 |
| Chr1_A_oryzae_RIB40:1541072-1541121 | AO090009000579 |
| Chr5_A_oryzae_RIB40:1189290-1189339 | AO090701000438 |
| Chr1_A_oryzae_RIB40:2018102-2018151 | AO090005001648 |
| Chr2_A_oryzae_RIB40:1931320-1931369 | AO090001000723 |
| Chr2_A_oryzae_RIB40:2121254-2121303 | AO090003000009 |
| Chr2_A_oryzae_RIB40:2121254-2121303 | AO090003000010 |
| Chr3_A_oryzae_RIB40:3036161-3036210 | AO090026000767 |
| Chr4_A_oryzae_RIB40:3304342-3304391 | AO090102000450 |
| Chr6_A_oryzae_RIB40:3149086-3149135 | AO090038000202 |
| Chr8_A_oryzae_RIB40:1186191-1186240 | AO090103000029 |
| Chr2_A_oryzae_RIB40:5160249-5160298 | AO090003001123 |
| Chr7_A_oryzae_RIB40:396380-396429 | AO090011000144 |
| Chr7_A_oryzae_RIB40:396380-396429 | AO090011000145 |
| Chr8_A_oryzae_RIB40:438569-438618 | AO090103000327 |
| Chr8_A_oryzae_RIB40:438569-438618 | AO090103000328 |
| Chr2_A_oryzae_RIB40:2441093-2441142 | AO090003001561 |
| Chr3_A_oryzae_RIB40:5064814-5064863 | AO090026000020 |
| Chr3_A_oryzae_RIB40:1165444-1165493 | AO090023000454 |
| Chr2_A_oryzae_RIB40:362552-362601 | AO090001000159 |
| Chr4_A_oryzae_RIB40:2464745-2464794 | AO090026000084 |
| Chr4_A_oryzae_RIB40:2464745-2464794 | AO090003000903 |
| Chr3_A_oryzae_RIB40:3988994-3989043 | AO090026000400 |
| Chr5_A_oryzae_RIB40:4294879-4294928 | AO090113000103 |
| Chr3_A_oryzae_RIB40:2831627-2831676 | AO090026000837 |
| Chr7_A_oryzae_RIB40:332987-333036 | AO090011000118 |
| Chr5_A_oryzae_RIB40:2070043-2070092 | AO090701000110 |
| Chr3_A_oryzae_RIB40:1285913-1285962 | AO090023000495 |
| Chr6_A_oryzae_RIB40:2738419-2738468 | AO090038000355 |
| Chr1_A_oryzae_RIB40:5317434-5317483 | AO090005000430 |
| Chr1_A_oryzae_RIB40:6272864-6272913 | AO090005000061 |
| Chr2_A_oryzae_RIB40:4087133-4087182 | AO090701001095 |
| Chr2_A_oryzae_RIB40:716193-716242 | AO090001000286 |
| Chr3_A_oryzae_RIB40:4133352-4133401 | AO090026000343 |
| Chr8_A_oryzae_RIB40:3160582-3160631 | AO090010000100 |
| Chr2_A_oryzae_RIB40:1750922-1750971 | AO090001000664 |
| Chr3_A_oryzae_RIB40:3113368-3113417 | AO090026000732 |
| Chr6_A_oryzae_RIB40:3082279-3082328 | AO090038000221 |
| Chr5_A_oryzae_RIB40:2901210-2901259 | AO090120000088 |
| Chr5_A_oryzae_RIB40:4426166-4426215 | AO090113000162 |
| Chr5_A_oryzae_RIB40:4426166-4426215 | AO090113000163 |
| Chr5_A_oryzae_RIB40:4134793-4134842 | AO090113000039 |
| Chr8_A_oryzae_RIB40:211737-211786 | AO090103000417 |
| Chr4_A_oryzae_RIB40:2076982-2077031 | AO090012000809 |
| Chr3_A_oryzae_RIB40:102890-102939 | AO090023000039 |
| Chr2_A_oryzae_RIB40:3839334-3839383 | AO090003000652 |
| Chr7_A_oryzae_RIB40:2037573-2037622 | AO090011000798 |
| Chr2_A_oryzae_RIB40:5278144-5278193 | AO090003001171 |
| Chr2_A_oryzae_RIB40:2906906-2906955 | AO090003000299 |
| Chr2_A_oryzae_RIB40:2960650-2960699 | AO090003000318 |
| Chr2_A_oryzae_RIB40:5340340-5340389 | AO090003001194 |
| Chr5_A_oryzae_RIB40:66313-66362 | AO090701000879 |
| Chr5_A_oryzae_RIB40:66313-66362 | AO090701000878 |
| Chr5_A_oryzae_RIB40:2129071-2129120 | AO090026000084 |
| Chr5_A_oryzae_RIB40:2129071-2129120 | AO090003000903 |
| Chr6_A_oryzae_RIB40:109035-109084 | AO090701001218 |
| Chr6_A_oryzae_RIB40:3298050-3298099 | AO090038000148 |
| Chr7_A_oryzae_RIB40:471530-471579 | AO090011000178 |
| Chr1_A_oryzae_RIB40:2923635-2923684 | AO090005001309 |
| Chr4_A_oryzae_RIB40:1175189-1175238 | AO090012000471 |
| Chr4_A_oryzae_RIB40:2820980-2821029 | AO090102000633 |
| Chr5_A_oryzae_RIB40:2182166-2182215 | AO090701000065 |
| Chr5_A_oryzae_RIB40:944201-944250 | AO090701000533 |
| Chr5_A_oryzae_RIB40:944201-944250 | AO090701000532 |
| Chr1_A_oryzae_RIB40:4478951-4479000 | AO090005001669 |
| Chr2_A_oryzae_RIB40:3556564-3556613 | AO090003000550 |
| Chr6_A_oryzae_RIB40:2428099-2428148 | AO090038000465 |
| Chr7_A_oryzae_RIB40:11312-11361 | AO090011000008 |
| Chr7_A_oryzae_RIB40:11312-11361 | AO090011000007 |
| Chr4_A_oryzae_RIB40:2200022-2200071 | AO090012000853 |
| Chr7_A_oryzae_RIB40:457632-457681 | AO090011000172 |
| Chr7_A_oryzae_RIB40:1437905-1437954 | AO090011000570 |
| Chr4_A_oryzae_RIB40:778829-778878 | AO090012000315 |
| Chr3_A_oryzae_RIB40:3285578-3285627 | AO090026000664 |
| Chr3_A_oryzae_RIB40:3285578-3285627 | AO090026000665 |
| Chr7_A_oryzae_RIB40:1572782-1572831 | AO090011000613 |
| Chr7_A_oryzae_RIB40:1572782-1572831 | AO090011000614 |
| Chr7_A_oryzae_RIB40:1729723-1729772 | AO090011000671 |
| Chr2_A_oryzae_RIB40:4073065-4073114 | AO090003000737 |
| Chr2_A_oryzae_RIB40:4240782-4240831 | AO090003000803 |
| Chr7_A_oryzae_RIB40:331030-331079 | AO090011000117 |
| Chr1_A_oryzae_RIB40:2109187-2109236 | AO090005001612 |
| Chr1_A_oryzae_RIB40:2109187-2109236 | AO090005001613 |
| Chr1_A_oryzae_RIB40:3300485-3300534 | AO090005001179 |
| Chr4_A_oryzae_RIB40:161236-161285 | AO090012000070 |
| Chr8_A_oryzae_RIB40:3024158-3024207 | AO090010000143 |
| Chr2_A_oryzae_RIB40:5727637-5727686 | AO090003001590 |
| Chr3_A_oryzae_RIB40:1502785-1502834 | AO090023000570 |
| Chr2_A_oryzae_RIB40:5461805-5461854 | AO090003001238 |
| Chr3_A_oryzae_RIB40:2943263-2943312 | AO090026000804 |
| Chr5_A_oryzae_RIB40:1679796-1679845 | AO090701000252 |
| Chr2_A_oryzae_RIB40:5942429-5942478 | AO090003001428 |
| Chr2_A_oryzae_RIB40:5942429-5942478 | AO090003001429 |
| Chr2_A_oryzae_RIB40:336471-336520 | AO090001000149 |
| Chr3_A_oryzae_RIB40:2045736-2045785 | AO090023000787 |
| Chr6_A_oryzae_RIB40:2416792-2416841 | AO090038000467 |
| Chr3_A_oryzae_RIB40:1210947-1210996 | AO090023000471 |
| Chr3_A_oryzae_RIB40:1210947-1210996 | AO090023000472 |
| Chr3_A_oryzae_RIB40:853884-853933 | AO090023000332 |
| Chr7_A_oryzae_RIB40:2742073-2742122 | AO090206000061 |
| Chr1_A_oryzae_RIB40:2819776-2819825 | AO090005001349 |
| Chr3_A_oryzae_RIB40:2345225-2345274 | AO090023000895 |
| Chr4_A_oryzae_RIB40:3588671-3588720 | AO090102000344 |
| Chr1_A_oryzae_RIB40:1893002-1893051 | AO090009000710 |
| Chr2_A_oryzae_RIB40:1088851-1088900 | AO090001000434 |
| Chr3_A_oryzae_RIB40:4105644-4105693 | AO090026000356 |
| Chr4_A_oryzae_RIB40:4089884-4089933 | AO090102000172 |
| Chr8_A_oryzae_RIB40:1781975-1782024 | AO090010000616 |
| Chr4_A_oryzae_RIB40:3026910-3026959 | AO090102000551 |
| Chr5_A_oryzae_RIB40:938476-938525 | AO090701000535 |
| Chr5_A_oryzae_RIB40:938476-938525 | AO090701000534 |
| Chr1_A_oryzae_RIB40:4711881-4711930 | AO090005000647 |
| Chr1_A_oryzae_RIB40:4711881-4711930 | AO090005000648 |
| Chr8_A_oryzae_RIB40:2097472-2097521 | AO090010000483 |
| Chr1_A_oryzae_RIB40:1001462-1001511 | AO090009000373 |
| Chr3_A_oryzae_RIB40:4986764-4986813 | AO090026000053 |
| Chr6_A_oryzae_RIB40:672226-672275 | AO090020000450 |
| Chr2_A_oryzae_RIB40:848081-848130 | AO090001000335 |
| Chr2_A_oryzae_RIB40:5983663-5983712 | AO090003001450 |
| Chr6_A_oryzae_RIB40:3353700-3353749 | AO090038000122 |
| Chr6_A_oryzae_RIB40:3353700-3353749 | AO090701000984 |
| Chr7_A_oryzae_RIB40:2101978-2102027 | AO090011000824 |
| Chr2_A_oryzae_RIB40:5391175-5391224 | AO090003001577 |
| Chr6_A_oryzae_RIB40:2399663-2399712 | AO090038000471 |
| Chr3_A_oryzae_RIB40:4860259-4860308 | AO090026000091 |
| Chr3_A_oryzae_RIB40:4860259-4860308 | AO090026000090 |
| Chr5_A_oryzae_RIB40:2759920-2759969 | AO090120000028 |
| Chr6_A_oryzae_RIB40:425353-425402 | AO090020000545 |
| Chr6_A_oryzae_RIB40:425353-425402 | AO090020000546 |
| Chr6_A_oryzae_RIB40:487773-487822 | AO090020000520 |
| Chr7_A_oryzae_RIB40:1638659-1638708 | AO090011000636 |
| Chr6_A_oryzae_RIB40:650576-650625 | AO090020000459 |
| Chr1_A_oryzae_RIB40:4568534-4568583 | AO090005001660 |
| Chr7_A_oryzae_RIB40:1273083-1273132 | AO090011000508 |
| Chr2_A_oryzae_RIB40:2629271-2629320 | AO090003000203 |
| Chr2_A_oryzae_RIB40:4395727-4395776 | AO090003000846 |
| Chr3_A_oryzae_RIB40:3354199-3354248 | AO090026000640 |
| Chr1_A_oryzae_RIB40:6480185-6480234 | AO090308000009 |
| Chr7_A_oryzae_RIB40:20998-21047 | AO090011000011 |
| Chr2_A_oryzae_RIB40:1187956-1188005 | AO090001000467 |
| Chr7_A_oryzae_RIB40:2187980-2188029 | AO090011000855 |
| Chr7_A_oryzae_RIB40:2187980-2188029 | AO090011000857 |
| Chr2_A_oryzae_RIB40:5925158-5925207 | AO090003001422 |
| Chr2_A_oryzae_RIB40:4541985-4542034 | AO090026000084 |
| Chr2_A_oryzae_RIB40:4541985-4542034 | AO090003000903 |
| Chr3_A_oryzae_RIB40:1045951-1046000 | AO090023000408 |
| Chr3_A_oryzae_RIB40:4099311-4099360 | AO090026000360 |
| Chr6_A_oryzae_RIB40:233266-233315 | AO090020000622 |
| Chr7_A_oryzae_RIB40:492376-492425 | AO090011000188 |
| Chr7_A_oryzae_RIB40:492376-492425 | AO090011000190 |
| Chr6_A_oryzae_RIB40:4003745-4003794 | AO090138000091 |
| Chr2_A_oryzae_RIB40:8356-8405 | AO090001000006 |
| Chr7_A_oryzae_RIB40:1525939-1525988 | AO090011000599 |
| Chr1_A_oryzae_RIB40:3837525-3837574 | AO090005000966 |
| Chr3_A_oryzae_RIB40:4972170-4972219 | AO090026000058 |
| Chr2_A_oryzae_RIB40:37832-37881 | AO090001000015 |
| Chr4_A_oryzae_RIB40:4464007-4464056 | AO090102000032 |
| Chr4_A_oryzae_RIB40:4464007-4464056 | AO090102000033 |
| Chr5_A_oryzae_RIB40:2123410-2123459 | AO090701000087 |
| Chr2_A_oryzae_RIB40:3265199-3265248 | AO090003000440 |
| Chr3_A_oryzae_RIB40:1414370-1414419 | AO090023000532 |
| Chr3_A_oryzae_RIB40:3461601-3461650 | AO090026000593 |
| Chr4_A_oryzae_RIB40:3356393-3356442 | AO090138000139 |
| Chr4_A_oryzae_RIB40:3356393-3356442 | AO090102000434 |
| Chr4_A_oryzae_RIB40:3356393-3356442 | AO090138000140 |
| Chr4_A_oryzae_RIB40:3356393-3356442 | AO090102000433 |
| Chr4_A_oryzae_RIB40:3758755-3758804 | AO090102000282 |
| Chr5_A_oryzae_RIB40:296945-296994 | AO090701000788 |
| Chr5_A_oryzae_RIB40:503062-503111 | AO090701000706 |
| Chr5_A_oryzae_RIB40:503062-503111 | AO090701001009 |
| Chr6_A_oryzae_RIB40:1680486-1680535 | AO090020000050 |
| Chr5_A_oryzae_RIB40:3695648-3695697 | AO090120000384 |
| Chr4_A_oryzae_RIB40:50681-50730 | AO090012000022 |
| Chr2_A_oryzae_RIB40:725727-725776 | AO090001000292 |
| Chr2_A_oryzae_RIB40:725727-725776 | AO090001000293 |
| Chr3_A_oryzae_RIB40:642230-642279 | AO090023000249 |
| Chr6_A_oryzae_RIB40:926931-926980 | AO090020000362 |
| Chr5_A_oryzae_RIB40:1905955-1906004 | AO090701000159 |
| Chr7_A_oryzae_RIB40:1420333-1420382 | AO090011000563 |
| Chr4_A_oryzae_RIB40:1202552-1202601 | AO090012000482 |
| Chr2_A_oryzae_RIB40:2321550-2321599 | AO090003000085 |
| Chr4_A_oryzae_RIB40:3498983-3499032 | AO090102000376 |
| Chr7_A_oryzae_RIB40:195572-195621 | AO090011000064 |
| Chr1_A_oryzae_RIB40:5289423-5289472 | AO090005000438 |
| Chr4_A_oryzae_RIB40:4808426-4808475 | AO090166000090 |
| Chr6_A_oryzae_RIB40:3717740-3717789 | AO090138000196 |
| Chr1_A_oryzae_RIB40:2408872-2408921 | AO090005001512 |
| Chr4_A_oryzae_RIB40:1434688-1434737 | AO090012000561 |
| Chr2_A_oryzae_RIB40:1661933-1661982 | AO090001000633 |
| Chr5_A_oryzae_RIB40:3688138-3688187 | AO090120000379 |
| Chr1_A_oryzae_RIB40:3833807-3833856 | AO090005000968 |
| Chr5_A_oryzae_RIB40:521455-521504 | AO090701000700 |
| Chr1_A_oryzae_RIB40:4121762-4121811 | AO090005000860 |
| Chr2_A_oryzae_RIB40:3225463-3225512 | AO090003001567 |
| Chr2_A_oryzae_RIB40:4908983-4909032 | AO090003001047 |
| Chr3_A_oryzae_RIB40:2303191-2303240 | AO090023000879 |
| Chr3_A_oryzae_RIB40:2810548-2810597 | AO090701001126 |
| Chr3_A_oryzae_RIB40:2810548-2810597 | AO090026000843 |
| Chr3_A_oryzae_RIB40:3804273-3804322 | AO090026000465 |
| Chr4_A_oryzae_RIB40:2826694-2826743 | AO090701001168 |
| Chr5_A_oryzae_RIB40:3727773-3727822 | AO090701001212 |
| Chr5_A_oryzae_RIB40:3733736-3733785 | AO090120000394 |
| Chr6_A_oryzae_RIB40:456218-456267 | AO090020000535 |
| Chr6_A_oryzae_RIB40:3225244-3225293 | AO090038000177 |
| Chr7_A_oryzae_RIB40:2835577-2835626 | AO090206000094 |
| Chr8_A_oryzae_RIB40:791441-791490 | AO090103000185 |
| Chr8_A_oryzae_RIB40:791441-791490 | AO090103000184 |
| Chr8_A_oryzae_RIB40:1165105-1165154 | AO090103000037 |
| Chr8_A_oryzae_RIB40:1539793-1539842 | AO090010000714 |
| Chr6_A_oryzae_RIB40:2421010-2421059 | AO090038000466 |
| Chr8_A_oryzae_RIB40:2704714-2704763 | AO090026000084 |
| Chr8_A_oryzae_RIB40:2704714-2704763 | AO090003000903 |
| Chr6_A_oryzae_RIB40:3349073-3349122 | AO090038000126 |
| Chr8_A_oryzae_RIB40:3352210-3352259 | AO090010000019 |
| Chr2_A_oryzae_RIB40:3085206-3085255 | AO090003000369 |
| Chr5_A_oryzae_RIB40:626105-626154 | AO090701000665 |
| Chr2_A_oryzae_RIB40:5361696-5361745 | AO090003001200 |
| Chr8_A_oryzae_RIB40:2354800-2354849 | AO090010000372 |
| Chr3_A_oryzae_RIB40:3052664-3052713 | AO090026000761 |
| Chr2_A_oryzae_RIB40:4926436-4926485 | AO090003001054 |
| Chr1_A_oryzae_RIB40:4451343-4451392 | AO090005000739 |
| Chr1_A_oryzae_RIB40:2215216-2215265 | AO090005001573 |
| Chr1_A_oryzae_RIB40:2523476-2523525 | AO090005001476 |
| Chr1_A_oryzae_RIB40:1617588-1617637 | AO090009000606 |
| Chr2_A_oryzae_RIB40:3676238-3676287 | AO090701001091 |
| Chr2_A_oryzae_RIB40:5447307-5447356 | AO090003001231 |
| Chr2_A_oryzae_RIB40:5447307-5447356 | AO090003001232 |
| Chr1_A_oryzae_RIB40:5318420-5318469 | AO090005000432 |
| Chr3_A_oryzae_RIB40:3242195-3242244 | AO090026000683 |
| Chr5_A_oryzae_RIB40:4341801-4341850 | AO090113000126 |
| Chr6_A_oryzae_RIB40:318630-318679 | AO090020000593 |
| Chr7_A_oryzae_RIB40:470950-470999 | AO090011000178 |
| Chr7_A_oryzae_RIB40:470950-470999 | AO090011000958 |
| Chr7_A_oryzae_RIB40:1405889-1405938 | AO090011000555 |
| Chr8_A_oryzae_RIB40:773615-773664 | AO090103000197 |
| Chr6_A_oryzae_RIB40:4140144-4140193 | AO090138000026 |
| Chr4_A_oryzae_RIB40:4385613-4385662 | AO090102000062 |
| Chr2_A_oryzae_RIB40:174805-174854 | AO090001000077 |
| Chr2_A_oryzae_RIB40:174805-174854 | AO090001000078 |
| Chr3_A_oryzae_RIB40:4037149-4037198 | AO090026000380 |
| Chr4_A_oryzae_RIB40:1500754-1500803 | AO090012000590 |
| Chr8_A_oryzae_RIB40:2862973-2863022 | AO090010000204 |
| Chr1_A_oryzae_RIB40:4339975-4340024 | AO090005000779 |
| Chr1_A_oryzae_RIB40:491836-491885 | AO090009000183 |
| Chr5_A_oryzae_RIB40:2267421-2267470 | AO090701000034 |
| Chr5_A_oryzae_RIB40:2267421-2267470 | AO090701000033 |
| Chr2_A_oryzae_RIB40:4017775-4017824 | AO090003000716 |
| Chr3_A_oryzae_RIB40:1706609-1706658 | AO090023000648 |
| Chr2_A_oryzae_RIB40:5509576-5509625 | AO090003001256 |
| Chr5_A_oryzae_RIB40:3848735-3848784 | AO090120000433 |
| Chr1_A_oryzae_RIB40:6284721-6284770 | AO090701000434 |
| Chr2_A_oryzae_RIB40:232725-232774 | AO090001000103 |
| Chr3_A_oryzae_RIB40:1004316-1004365 | AO090023000390 |
| Chr4_A_oryzae_RIB40:1184014-1184063 | AO090012000476 |
| Chr4_A_oryzae_RIB40:1184014-1184063 | AO090012000475 |
| Chr5_A_oryzae_RIB40:1169791-1169840 | AO090701000446 |
| mito_A_oryzae_RIB40:13334-13383 | AO090002000070 |
| Chr5_A_oryzae_RIB40:1875400-1875449 | AO090701000175 |
| Chr4_A_oryzae_RIB40:1593777-1593826 | AO090012000623 |
| Chr3_A_oryzae_RIB40:2214598-2214647 | AO090023000848 |
| Chr3_A_oryzae_RIB40:2214598-2214647 | AO090023000847 |
| Chr1_A_oryzae_RIB40:792422-792471 | AO090009000296 |
| Chr6_A_oryzae_RIB40:2851089-2851138 | AO090038000306 |
| Chr8_A_oryzae_RIB40:2305128-2305177 | AO090010000401 |
| Chr2_A_oryzae_RIB40:212257-212306 | AO090001000095 |
| Chr2_A_oryzae_RIB40:4731234-4731283 | AO090003000972 |
| Chr5_A_oryzae_RIB40:885802-885851 | AO090701000556 |
| Chr2_A_oryzae_RIB40:2336844-2336893 | AO090003000094 |
| Chr2_A_oryzae_RIB40:4921652-4921701 | AO090003001053 |
| Chr1_A_oryzae_RIB40:6046887-6046936 | AO090005000147 |
| Chr2_A_oryzae_RIB40:4651570-4651619 | AO090003000943 |
| Chr2_A_oryzae_RIB40:5315851-5315900 | AO090003001182 |
| Chr2_A_oryzae_RIB40:5315851-5315900 | AO090003001184 |
| Chr1_A_oryzae_RIB40:3198408-3198457 | AO090005001219 |
| Chr2_A_oryzae_RIB40:2324886-2324935 | AO090003000089 |
| Chr3_A_oryzae_RIB40:961668-961717 | AO090701001121 |
| Chr4_A_oryzae_RIB40:3982589-3982638 | AO090102000216 |
| Chr5_A_oryzae_RIB40:191228-191277 | AO090701000831 |
| Chr5_A_oryzae_RIB40:191228-191277 | AO090701000830 |
| Chr3_A_oryzae_RIB40:3672750-3672799 | AO090026000517 |
| Chr4_A_oryzae_RIB40:2665746-2665795 | AO090012001027 |
| Chr4_A_oryzae_RIB40:3764851-3764900 | AO090701001179 |
| Chr5_A_oryzae_RIB40:2586795-2586844 | AO090124000040 |
| Chr6_A_oryzae_RIB40:3863634-3863683 | AO090138000139 |
| Chr6_A_oryzae_RIB40:3863634-3863683 | AO090102000434 |
| Chr6_A_oryzae_RIB40:3863634-3863683 | AO090138000140 |
| Chr6_A_oryzae_RIB40:3863634-3863683 | AO090102000433 |
| Chr7_A_oryzae_RIB40:1850412-1850461 | AO090011000722 |
| Chr5_A_oryzae_RIB40:2861376-2861425 | AO090120000078 |
| Chr5_A_oryzae_RIB40:2861376-2861425 | AO090120000079 |
| Chr3_A_oryzae_RIB40:1790591-1790640 | AO090023000682 |
| Chr5_A_oryzae_RIB40:324950-324999 | AO090701000774 |
| Chr5_A_oryzae_RIB40:1877592-1877641 | AO090701000174 |
| Chr5_A_oryzae_RIB40:1877592-1877641 | AO090701000173 |
| Chr6_A_oryzae_RIB40:297500-297549 | AO090020000603 |
| Chr5_A_oryzae_RIB40:3161773-3161822 | AO090120000191 |
| Chr2_A_oryzae_RIB40:5560949-5560998 | AO090003001278 |
| Chr8_A_oryzae_RIB40:1082013-1082062 | AO090103000067 |
| Chr3_A_oryzae_RIB40:1075542-1075591 | AO090023000421 |
| Chr1_A_oryzae_RIB40:71278-71327 | AO090009000022 |
| Chr1_A_oryzae_RIB40:191750-191799 | AO090009000067 |
| Chr2_A_oryzae_RIB40:2210989-2211038 | AO090003000045 |
| Chr3_A_oryzae_RIB40:4831787-4831836 | AO090026000102 |
| Chr4_A_oryzae_RIB40:903200-903249 | AO090012000366 |
| Chr4_A_oryzae_RIB40:3827367-3827416 | AO090102000263 |
| Chr7_A_oryzae_RIB40:2451044-2451093 | AO090011000932 |
| Chr8_A_oryzae_RIB40:1193025-1193074 | AO090701000434 |
| Chr5_A_oryzae_RIB40:2946386-2946435 | AO090120000107 |
| Chr6_A_oryzae_RIB40:459881-459930 | AO090020000531 |
| Chr7_A_oryzae_RIB40:229877-229926 | AO090011000078 |
| Chr5_A_oryzae_RIB40:3484335-3484384 | AO090120000304 |
| Chr8_A_oryzae_RIB40:1446563-1446612 | AO090010000749 |
| Chr2_A_oryzae_RIB40:4815293-4815342 | AO090003001003 |
| Chr1_A_oryzae_RIB40:3919387-3919436 | AO090005000937 |
| Chr2_A_oryzae_RIB40:1721855-1721904 | AO090001000651 |
| Chr3_A_oryzae_RIB40:3523735-3523784 | AO090026000570 |
| Chr7_A_oryzae_RIB40:502518-502567 | AO090011000195 |
| Chr1_A_oryzae_RIB40:4902253-4902302 | AO090005000582 |
| Chr1_A_oryzae_RIB40:84592-84641 | AO090009000029 |
| Chr3_A_oryzae_RIB40:699588-699637 | AO090023000272 |
| Chr2_A_oryzae_RIB40:1889866-1889915 | AO090001000712 |
| Chr5_A_oryzae_RIB40:3796425-3796474 | AO090120000417 |
| Chr7_A_oryzae_RIB40:663316-663365 | AO090011000257 |
| Chr5_A_oryzae_RIB40:4173976-4174025 | AO090113000054 |
| Chr4_A_oryzae_RIB40:3976120-3976169 | AO090102000217 |
| Chr4_A_oryzae_RIB40:4297487-4297536 | AO090102000097 |
| Chr1_A_oryzae_RIB40:5111068-5111117 | AO090005000501 |
| Chr2_A_oryzae_RIB40:655850-655899 | AO090001000263 |
| Chr2_A_oryzae_RIB40:4090387-4090436 | AO090003000743 |
| Chr2_A_oryzae_RIB40:5150402-5150451 | AO090003001120 |
| Chr3_A_oryzae_RIB40:2484655-2484704 | AO090023000942 |
| Chr3_A_oryzae_RIB40:2484655-2484704 | AO090120000198 |
| Chr3_A_oryzae_RIB40:2484655-2484704 | AO090023000974 |
| Chr3_A_oryzae_RIB40:2484655-2484704 | AO090701001123 |
| Chr4_A_oryzae_RIB40:1909439-1909488 | AO090012000751 |
| Chr5_A_oryzae_RIB40:976826-976875 | AO090701001191 |
| Chr5_A_oryzae_RIB40:1263709-1263758 | AO090701000408 |
| Chr6_A_oryzae_RIB40:735593-735642 | AO090020000434 |
| Chr6_A_oryzae_RIB40:2433123-2433172 | AO090038000464 |
| Chr8_A_oryzae_RIB40:1922447-1922496 | AO090010000554 |
| Chr8_A_oryzae_RIB40:2482434-2482483 | AO090010000322 |
| Chr1_A_oryzae_RIB40:6269426-6269475 | AO090005000063 |
| Chr2_A_oryzae_RIB40:4694229-4694278 | AO090003000959 |
| Chr3_A_oryzae_RIB40:1555435-1555484 | AO090023000587 |
| Chr8_A_oryzae_RIB40:2402828-2402877 | AO090010000355 |
| Chr1_A_oryzae_RIB40:4002752-4002801 | AO090005000905 |
| Chr1_A_oryzae_RIB40:4002752-4002801 | AO090005000906 |
| Chr1_A_oryzae_RIB40:5805442-5805491 | AO090005000238 |
| Chr3_A_oryzae_RIB40:563828-563877 | AO090023000225 |
| Chr3_A_oryzae_RIB40:3249606-3249655 | AO090026000681 |
| Chr3_A_oryzae_RIB40:71242-71291 | AO090023000025 |
| Chr3_A_oryzae_RIB40:71242-71291 | AO090023000026 |
| Chr6_A_oryzae_RIB40:1117284-1117333 | AO090701000970 |
| Chr2_A_oryzae_RIB40:1137416-1137465 | AO090001000448 |
| Chr8_A_oryzae_RIB40:1398329-1398378 | AO090010000767 |
| Chr8_A_oryzae_RIB40:1398329-1398378 | AO090010000768 |
| Chr1_A_oryzae_RIB40:5598504-5598553 | AO090005000321 |
| Chr1_A_oryzae_RIB40:5598504-5598553 | AO090005000323 |
| Chr3_A_oryzae_RIB40:813118-813167 | AO090023000319 |
| Chr8_A_oryzae_RIB40:1388425-1388474 | AO090010000770 |
| Chr2_A_oryzae_RIB40:1624827-1624876 | AO090001000622 |
| Chr3_A_oryzae_RIB40:464889-464938 | AO090023000186 |
| Chr8_A_oryzae_RIB40:2550266-2550315 | AO090701001258 |
| Chr2_A_oryzae_RIB40:5285128-5285177 | AO090003001173 |
| Chr3_A_oryzae_RIB40:1438683-1438732 | AO090023000540 |
| Chr3_A_oryzae_RIB40:1893358-1893407 | AO090023000718 |
| Chr5_A_oryzae_RIB40:1133977-1134026 | AO090701000463 |
| Chr6_A_oryzae_RIB40:190756-190805 | AO090020000644 |
| Chr7_A_oryzae_RIB40:2503292-2503341 | AO090011000955 |
| Chr7_A_oryzae_RIB40:2609665-2609714 | AO090206000006 |
| Chr7_A_oryzae_RIB40:2609665-2609714 | AO090206000009 |
| Chr8_A_oryzae_RIB40:484709-484758 | AO090103000307 |
| Chr3_A_oryzae_RIB40:1955992-1956041 | AO090023000746 |
| Chr6_A_oryzae_RIB40:631693-631742 | AO090020000468 |
| Chr7_A_oryzae_RIB40:1076800-1076849 | AO090011000423 |
| Chr2_A_oryzae_RIB40:3210846-3210895 | AO090003000422 |
| Chr3_A_oryzae_RIB40:3887240-3887289 | AO090026000436 |
| Chr4_A_oryzae_RIB40:879169-879218 | AO090012000356 |
| Chr1_A_oryzae_RIB40:732397-732446 | AO090701001017 |
| Chr6_A_oryzae_RIB40:2017007-2017056 | AO090038000605 |
| Chr8_A_oryzae_RIB40:2310296-2310345 | AO090010000398 |
| Chr3_A_oryzae_RIB40:2397974-2398023 | AO090023000914 |
| Chr2_A_oryzae_RIB40:1770071-1770120 | AO090001000673 |
| Chr7_A_oryzae_RIB40:1916216-1916265 | AO090701000956 |
| Chr7_A_oryzae_RIB40:518538-518587 | AO090011000199 |
| Chr1_A_oryzae_RIB40:47258-47307 | AO090009000012 |
| Chr7_A_oryzae_RIB40:2262555-2262604 | AO090011000883 |
| Chr4_A_oryzae_RIB40:1436885-1436934 | AO090012000564 |
| Chr7_A_oryzae_RIB40:1398227-1398276 | AO090011000553 |
| Chr2_A_oryzae_RIB40:2931109-2931158 | AO090003000309 |
| Chr4_A_oryzae_RIB40:1767277-1767326 | AO090012000696 |
| Chr7_A_oryzae_RIB40:16987-17036 | AO090011000009 |
| Chr2_A_oryzae_RIB40:5006295-5006344 | AO090003001075 |
| Chr4_A_oryzae_RIB40:2290689-2290738 | AO090012000883 |
| Chr1_A_oryzae_RIB40:1747097-1747146 | AO090009000654 |
| Chr1_A_oryzae_RIB40:1747097-1747146 | AO090009000653 |
| Chr1_A_oryzae_RIB40:2083208-2083257 | AO090005001625 |
| Chr1_A_oryzae_RIB40:2124957-2125006 | AO090005001605 |
| Chr1_A_oryzae_RIB40:6500981-6501030 | AO090308000019 |
| Chr2_A_oryzae_RIB40:4666397-4666446 | AO090003000949 |
| Chr2_A_oryzae_RIB40:4666397-4666446 | AO090003001573 |
| Chr2_A_oryzae_RIB40:6079125-6079174 | AO090003001490 |
| Chr3_A_oryzae_RIB40:334333-334382 | AO090023000130 |
| Chr4_A_oryzae_RIB40:340081-340130 | AO090012000139 |
| Chr4_A_oryzae_RIB40:340081-340130 | AO090012000141 |
| Chr4_A_oryzae_RIB40:4629454-4629503 | AO090166000026 |
| Chr5_A_oryzae_RIB40:1239188-1239237 | AO090701000419 |
| Chr6_A_oryzae_RIB40:3836012-3836061 | AO090138000209 |
| Chr8_A_oryzae_RIB40:609027-609076 | AO090103000256 |
| Chr8_A_oryzae_RIB40:1437119-1437168 | AO090010000752 |
| Chr8_A_oryzae_RIB40:2160123-2160172 | AO090010000462 |
| Chr8_A_oryzae_RIB40:2413442-2413491 | AO090010000353 |
| Chr8_A_oryzae_RIB40:2732386-2732435 | AO090010000246 |
| Chr8_A_oryzae_RIB40:29762-29811 | AO090103000498 |
| Chr8_A_oryzae_RIB40:29762-29811 | AO090103000499 |
| Chr4_A_oryzae_RIB40:71061-71110 | AO090012000034 |
| Chr4_A_oryzae_RIB40:71061-71110 | AO090012000033 |
| Chr7_A_oryzae_RIB40:1798345-1798394 | AO090011000699 |
| Chr2_A_oryzae_RIB40:1313541-1313590 | AO090001000510 |
| Chr4_A_oryzae_RIB40:315046-315095 | AO090012000130 |
| Chr2_A_oryzae_RIB40:2862313-2862362 | AO090003000284 |
| Chr4_A_oryzae_RIB40:4204687-4204736 | AO090102000133 |
| Chr6_A_oryzae_RIB40:2227942-2227991 | AO090038000535 |
| contig_28.1_A_oryzae_RIB40:1958-2007 | AO090663000001 |
| Chr6_A_oryzae_RIB40:2262062-2262111 | AO090038000524 |
| Chr1_A_oryzae_RIB40:2170418-2170467 | AO090005001593 |
| Chr2_A_oryzae_RIB40:876259-876308 | AO090001000348 |
| Chr3_A_oryzae_RIB40:3224054-3224103 | AO090026000692 |
| Chr3_A_oryzae_RIB40:3224054-3224103 | AO090026000691 |
| Chr2_A_oryzae_RIB40:4878284-4878333 | AO090003001032 |
| Chr1_A_oryzae_RIB40:1819869-1819918 | AO090009000679 |
| Chr2_A_oryzae_RIB40:3390823-3390872 | AO090003000484 |
| Chr2_A_oryzae_RIB40:4856485-4856534 | AO090003001022 |
| Chr2_A_oryzae_RIB40:4856485-4856534 | AO090701001103 |
| Chr2_A_oryzae_RIB40:6170413-6170462 | AO090003001521 |
| Chr1_A_oryzae_RIB40:1651138-1651187 | AO090701001021 |
| Chr1_A_oryzae_RIB40:2457595-2457644 | AO090005001498 |
| Chr1_A_oryzae_RIB40:4727122-4727171 | AO090005000642 |
| Chr2_A_oryzae_RIB40:412560-412609 | AO090001000180 |
| Chr2_A_oryzae_RIB40:1050364-1050413 | AO090001000415 |
| Chr2_A_oryzae_RIB40:1959665-1959714 | AO090001000734 |
| Chr3_A_oryzae_RIB40:183815-183864 | AO090023000069 |
| Chr3_A_oryzae_RIB40:824010-824059 | AO090023000323 |
| Chr3_A_oryzae_RIB40:1689323-1689372 | AO090023000643 |
| Chr3_A_oryzae_RIB40:4431628-4431677 | AO090026000245 |
| Chr4_A_oryzae_RIB40:4635249-4635298 | AO090166000029 |
| Chr4_A_oryzae_RIB40:4830977-4831026 | AO090166000099 |
| Chr5_A_oryzae_RIB40:3374262-3374311 | AO090120000264 |
| Chr5_A_oryzae_RIB40:3889203-3889252 | AO090120000450 |
| Chr5_A_oryzae_RIB40:4210758-4210807 | AO090113000066 |
| Chr6_A_oryzae_RIB40:1796790-1796839 | AO090020000012 |
| Chr7_A_oryzae_RIB40:2673240-2673289 | AO090206000037 |
| Chr7_A_oryzae_RIB40:2722347-2722396 | AO090206000057 |
| Chr7_A_oryzae_RIB40:2722347-2722396 | AO090206000056 |
| Chr8_A_oryzae_RIB40:3114182-3114231 | AO090010000112 |
| Chr7_A_oryzae_RIB40:741236-741285 | AO090011000290 |
| Chr7_A_oryzae_RIB40:741236-741285 | AO090011000289 |
| Chr1_A_oryzae_RIB40:2623250-2623299 | AO090005001435 |
| Chr1_A_oryzae_RIB40:2623250-2623299 | AO090005001671 |
| Chr2_A_oryzae_RIB40:3391504-3391553 | AO090003000484 |
| Chr7_A_oryzae_RIB40:885776-885825 | AO090011000345 |
| Chr6_A_oryzae_RIB40:2111486-2111535 | AO090038000570 |
| Chr6_A_oryzae_RIB40:2924688-2924737 | AO090038000281 |
| Chr1_A_oryzae_RIB40:2597908-2597957 | AO090005001450 |
| Chr2_A_oryzae_RIB40:3785073-3785122 | AO090003000629 |
| Chr7_A_oryzae_RIB40:1166906-1166955 | AO090011000461 |
| Chr7_A_oryzae_RIB40:2058276-2058325 | AO090011000808 |
| Chr1_A_oryzae_RIB40:6105501-6105550 | AO090005000126 |
| Chr2_A_oryzae_RIB40:1611471-1611520 | AO090001000615 |
| Chr2_A_oryzae_RIB40:1611471-1611520 | AO090001000614 |
| Chr4_A_oryzae_RIB40:1189543-1189592 | AO090012000479 |
| Chr2_A_oryzae_RIB40:5755268-5755317 | AO090003001351 |
| Chr2_A_oryzae_RIB40:5755268-5755317 | AO090003001350 |
| Chr3_A_oryzae_RIB40:4457933-4457982 | AO090026000237 |
| Chr5_A_oryzae_RIB40:3651121-3651170 | AO090120000364 |
| Chr3_A_oryzae_RIB40:2055674-2055723 | AO090023000791 |
| Chr6_A_oryzae_RIB40:2781153-2781202 | AO090038000335 |
| Chr6_A_oryzae_RIB40:2781153-2781202 | AO090038000337 |
| Chr4_A_oryzae_RIB40:1779358-1779407 | AO090012000700 |
| Chr6_A_oryzae_RIB40:4127412-4127461 | AO090138000033 |
| Chr1_A_oryzae_RIB40:957754-957803 | AO090009000357 |
| Chr1_A_oryzae_RIB40:2026721-2026770 | AO090005001647 |
| Chr1_A_oryzae_RIB40:4472843-4472892 | AO090005000731 |
| Chr2_A_oryzae_RIB40:2562442-2562491 | AO090701001082 |
| Chr4_A_oryzae_RIB40:573424-573473 | AO090701000960 |
| Chr4_A_oryzae_RIB40:573424-573473 | AO090012000231 |
| Chr4_A_oryzae_RIB40:4633805-4633854 | AO090166000027 |
| Chr7_A_oryzae_RIB40:1272423-1272472 | AO090011000508 |
| Chr7_A_oryzae_RIB40:2622781-2622830 | AO090206000015 |
| Chr3_A_oryzae_RIB40:3790972-3791021 | AO090026000470 |
| Chr7_A_oryzae_RIB40:847064-847113 | AO090011000332 |
| Chr2_A_oryzae_RIB40:1282585-1282634 | AO090001000498 |
| Chr1_A_oryzae_RIB40:2712661-2712710 | AO090005001401 |
| Chr3_A_oryzae_RIB40:2353010-2353059 | AO090023000899 |
| Chr3_A_oryzae_RIB40:2353010-2353059 | AO090023000898 |
| Chr2_A_oryzae_RIB40:1503771-1503820 | AO090001000572 |
| Chr2_A_oryzae_RIB40:1503771-1503820 | AO090001000571 |
| Chr1_A_oryzae_RIB40:4481765-4481814 | AO090005000727 |
| Chr2_A_oryzae_RIB40:3578988-3579037 | AO090003000557 |
| Chr4_A_oryzae_RIB40:1943295-1943344 | AO090012000763 |
| Chr2_A_oryzae_RIB40:819693-819742 | AO090001000322 |
| Chr1_A_oryzae_RIB40:2549809-2549858 | AO090005001467 |
| Chr2_A_oryzae_RIB40:424918-424967 | AO090001000186 |
| Chr3_A_oryzae_RIB40:4675403-4675452 | AO090026000158 |
| Chr1_A_oryzae_RIB40:3062815-3062864 | AO090005001266 |
| Chr8_A_oryzae_RIB40:3047858-3047907 | AO090010000135 |
| Chr2_A_oryzae_RIB40:2308617-2308666 | AO090003000080 |
| Chr5_A_oryzae_RIB40:4077151-4077200 | AO090113000012 |
| Chr8_A_oryzae_RIB40:314352-314401 | AO090103000371 |
| Chr8_A_oryzae_RIB40:314352-314401 | AO090103000370 |
| Chr2_A_oryzae_RIB40:837407-837456 | AO090001000328 |
| Chr8_A_oryzae_RIB40:1097755-1097804 | AO090103000061 |
| Chr1_A_oryzae_RIB40:3273349-3273398 | AO090005001192 |
| Chr1_A_oryzae_RIB40:3273349-3273398 | AO090005001193 |
| Chr1_A_oryzae_RIB40:3982915-3982964 | AO090005000915 |
| Chr3_A_oryzae_RIB40:174058-174107 | AO090023000063 |
| Chr3_A_oryzae_RIB40:303174-303223 | AO090023000121 |
| Chr3_A_oryzae_RIB40:3573715-3573764 | AO090026000552 |
| Chr4_A_oryzae_RIB40:297286-297335 | AO090012000122 |
| Chr4_A_oryzae_RIB40:1664903-1664952 | AO090012000655 |
| Chr4_A_oryzae_RIB40:1744802-1744851 | AO090012000688 |
| Chr4_A_oryzae_RIB40:2896531-2896580 | AO090102000602 |
| Chr4_A_oryzae_RIB40:4579551-4579600 | AO090166000010 |
| Chr4_A_oryzae_RIB40:4673236-4673285 | AO090166000046 |
| Chr5_A_oryzae_RIB40:2274039-2274088 | AO090701000031 |
| Chr5_A_oryzae_RIB40:2274039-2274088 | AO090701000030 |
| Chr5_A_oryzae_RIB40:2700035-2700084 | AO090120000003 |
| Chr5_A_oryzae_RIB40:3162882-3162931 | AO090120000192 |
| Chr5_A_oryzae_RIB40:3886751-3886800 | AO090120000448 |
| Chr6_A_oryzae_RIB40:1263921-1263970 | AO090020000222 |
| Chr6_A_oryzae_RIB40:1475570-1475619 | AO090020000135 |
| Chr1_A_oryzae_RIB40:1284140-1284189 | AO090009000485 |
| Chr2_A_oryzae_RIB40:2547165-2547214 | AO090003000166 |
| Chr4_A_oryzae_RIB40:4483856-4483905 | AO090102000026 |
| Chr7_A_oryzae_RIB40:1692072-1692121 | AO090011000650 |
| Chr2_A_oryzae_RIB40:2402983-2403032 | AO090003000115 |
| Chr4_A_oryzae_RIB40:237647-237696 | AO090012000098 |
| Chr8_A_oryzae_RIB40:832942-832991 | AO090103000167 |
| Chr2_A_oryzae_RIB40:4212030-4212079 | AO090003000790 |
| Chr5_A_oryzae_RIB40:2626555-2626604 | AO090124000022 |
| Chr6_A_oryzae_RIB40:3975683-3975732 | AO090138000104 |
| Chr6_A_oryzae_RIB40:3975683-3975732 | AO090138000105 |
| Chr7_A_oryzae_RIB40:2200989-2201038 | AO090011000860 |
| Chr8_A_oryzae_RIB40:2629273-2629322 | AO090010000279 |
| Chr8_A_oryzae_RIB40:2629273-2629322 | AO090010000254 |
| Chr2_A_oryzae_RIB40:876809-876858 | AO090001000348 |
| Chr3_A_oryzae_RIB40:2914138-2914187 | AO090026000810 |
| Chr4_A_oryzae_RIB40:3006624-3006673 | AO090102000558 |
| Chr2_A_oryzae_RIB40:1987729-1987778 | AO090001000747 |
| Chr1_A_oryzae_RIB40:5255750-5255799 | AO090005000449 |
| Chr2_A_oryzae_RIB40:2329862-2329911 | AO090003000092 |
| Chr5_A_oryzae_RIB40:2784417-2784466 | AO090120000043 |
| Chr3_A_oryzae_RIB40:4138311-4138360 | AO090026000342 |
| Chr3_A_oryzae_RIB40:3101629-3101678 | AO090026000738 |
| Chr5_A_oryzae_RIB40:83689-83738 | AO090701000872 |
| Chr2_A_oryzae_RIB40:927269-927318 | AO090001000759 |
| Chr3_A_oryzae_RIB40:1918041-1918090 | AO090023000728 |
| Chr2_A_oryzae_RIB40:5994312-5994361 | AO090701001113 |
| Chr2_A_oryzae_RIB40:3972524-3972573 | AO090003000695 |
| Chr1_A_oryzae_RIB40:66411-66460 | AO090009000019 |
| Chr1_A_oryzae_RIB40:5068572-5068621 | AO090005000517 |
| Chr1_A_oryzae_RIB40:5068572-5068621 | AO090005000518 |
| Chr1_A_oryzae_RIB40:5332378-5332427 | AO090005000427 |
| Chr1_A_oryzae_RIB40:5332378-5332427 | AO090005000426 |
| Chr1_A_oryzae_RIB40:5490163-5490212 | AO090005000371 |
| Chr1_A_oryzae_RIB40:5490163-5490212 | AO090005000372 |
| Chr2_A_oryzae_RIB40:2810520-2810569 | AO090003000268 |
| Chr2_A_oryzae_RIB40:4087803-4087852 | AO090701001095 |
| Chr2_A_oryzae_RIB40:4432565-4432614 | AO090003000861 |
| Chr2_A_oryzae_RIB40:4666995-4667044 | AO090003001573 |
| Chr2_A_oryzae_RIB40:4666995-4667044 | AO090003000949 |
| Chr2_A_oryzae_RIB40:5833608-5833657 | AO090003001385 |
| Chr2_A_oryzae_RIB40:5904594-5904643 | AO090003001414 |
| Chr3_A_oryzae_RIB40:2946909-2946958 | AO090026000803 |
| Chr5_A_oryzae_RIB40:4340373-4340422 | AO090113000125 |
| Chr5_A_oryzae_RIB40:4497728-4497777 | AO090113000193 |
| Chr5_A_oryzae_RIB40:4497728-4497777 | AO090113000192 |
| Chr6_A_oryzae_RIB40:1757885-1757934 | AO090701001237 |
| Chr6_A_oryzae_RIB40:1757885-1757934 | AO090020000027 |
| Chr6_A_oryzae_RIB40:3591420-3591469 | AO090038000028 |
| Chr8_A_oryzae_RIB40:2697734-2697783 | AO090010000279 |
| Chr8_A_oryzae_RIB40:2697734-2697783 | AO090010000254 |
| Chr1_A_oryzae_RIB40:5687149-5687198 | AO090005000281 |
| Chr7_A_oryzae_RIB40:1046775-1046824 | AO090011000410 |
| Chr8_A_oryzae_RIB40:270310-270359 | AO090103000390 |
| Chr1_A_oryzae_RIB40:3232494-3232543 | AO090005001209 |
| Chr5_A_oryzae_RIB40:181773-181822 | AO090701000832 |
| Chr6_A_oryzae_RIB40:2163412-2163461 | AO090038000551 |
| Chr2_A_oryzae_RIB40:5217183-5217232 | AO090003001140 |
| Chr3_A_oryzae_RIB40:558303-558352 | AO090023000222 |
| Chr3_A_oryzae_RIB40:558303-558352 | AO090023000223 |
| Chr3_A_oryzae_RIB40:1255773-1255822 | AO090023000486 |
| Chr7_A_oryzae_RIB40:928467-928516 | AO090011000361 |
| Chr1_A_oryzae_RIB40:1480654-1480703 | AO090009000564 |
| Chr6_A_oryzae_RIB40:3584756-3584805 | AO090038000029 |
| Chr6_A_oryzae_RIB40:939248-939297 | AO090020000357 |
| Chr5_A_oryzae_RIB40:4274141-4274190 | AO090113000092 |
| Chr5_A_oryzae_RIB40:4274141-4274190 | AO090113000091 |
| Chr8_A_oryzae_RIB40:2019184-2019233 | AO090010000516 |
| Chr1_A_oryzae_RIB40:2420545-2420594 | AO090005001510 |
| Chr6_A_oryzae_RIB40:3646170-3646219 | AO090701001258 |
| Chr8_A_oryzae_RIB40:809900-809949 | AO090103000178 |
| Chr1_A_oryzae_RIB40:3908804-3908853 | AO090005000942 |
| Chr4_A_oryzae_RIB40:859687-859736 | AO090012000350 |
| Chr2_A_oryzae_RIB40:2515289-2515338 | AO090003000157 |
| Chr5_A_oryzae_RIB40:3620580-3620629 | AO090120000349 |
| Chr7_A_oryzae_RIB40:1125841-1125890 | AO090011000444 |
| Chr3_A_oryzae_RIB40:2388116-2388165 | AO090023000910 |
| Chr5_A_oryzae_RIB40:3987519-3987568 | AO090120000485 |
| Chr7_A_oryzae_RIB40:2771092-2771141 | AO090701001279 |
| Chr1_A_oryzae_RIB40:1712443-1712492 | AO090009000641 |
| Chr3_A_oryzae_RIB40:3971513-3971562 | AO090026000408 |
| Chr7_A_oryzae_RIB40:1960422-1960471 | AO090011000768 |
| Chr1_A_oryzae_RIB40:3974393-3974442 | AO090005000918 |
| Chr5_A_oryzae_RIB40:2645613-2645662 | AO090124000015 |
| Chr5_A_oryzae_RIB40:3925630-3925679 | AO090120000461 |
| Chr1_A_oryzae_RIB40:5789551-5789600 | AO090005000244 |
| Chr2_A_oryzae_RIB40:1561353-1561402 | AO090001000596 |
| Chr2_A_oryzae_RIB40:3021190-3021239 | AO090003000341 |
| Chr3_A_oryzae_RIB40:2644628-2644677 | AO090023001002 |
| Chr4_A_oryzae_RIB40:1815002-1815051 | AO090012000711 |
| Chr4_A_oryzae_RIB40:1815002-1815051 | AO090012000712 |
| Chr4_A_oryzae_RIB40:1890095-1890144 | AO090012000741 |
| Chr4_A_oryzae_RIB40:3394771-3394820 | AO090102000415 |
| Chr4_A_oryzae_RIB40:3394771-3394820 | AO090102000417 |
| Chr5_A_oryzae_RIB40:2021558-2021607 | AO090701000121 |
| Chr7_A_oryzae_RIB40:406951-407000 | AO090011000149 |
| Chr7_A_oryzae_RIB40:454365-454414 | AO090011000170 |
| Chr8_A_oryzae_RIB40:2905289-2905338 | AO090010000191 |
| Chr2_A_oryzae_RIB40:85729-85778 | AO090001000039 |
| Chr4_A_oryzae_RIB40:3934606-3934655 | AO090102000229 |
| Chr4_A_oryzae_RIB40:4058932-4058981 | AO090102000183 |
| Chr4_A_oryzae_RIB40:135032-135081 | AO090012000058 |
| Chr3_A_oryzae_RIB40:1200852-1200901 | AO090023000467 |
| Chr1_A_oryzae_RIB40:3291014-3291063 | AO090005001183 |
| Chr3_A_oryzae_RIB40:1349953-1350002 | AO090023000518 |
| Chr3_A_oryzae_RIB40:3549337-3549386 | AO090026000561 |
| Chr5_A_oryzae_RIB40:145315-145364 | AO090701000846 |
| Chr5_A_oryzae_RIB40:4394590-4394639 | AO090113000146 |
| Chr7_A_oryzae_RIB40:2005835-2005884 | AO090011000792 |
| Chr2_A_oryzae_RIB40:3922304-3922353 | AO090003000677 |
| Chr8_A_oryzae_RIB40:1113774-1113823 | AO090103000051 |
| Chr5_A_oryzae_RIB40:3853763-3853812 | AO090120000437 |
| Chr1_A_oryzae_RIB40:2697797-2697846 | AO090005001407 |
| Chr6_A_oryzae_RIB40:1375023-1375072 | AO090020000180 |
| Chr2_A_oryzae_RIB40:283551-283600 | AO090001000124 |
| Chr2_A_oryzae_RIB40:283551-283600 | AO090001000123 |
| Chr4_A_oryzae_RIB40:2181599-2181648 | AO090012000846 |
| Chr6_A_oryzae_RIB40:196246-196295 | AO090020000640 |
| Chr6_A_oryzae_RIB40:2156898-2156947 | AO090038000554 |
| Chr3_A_oryzae_RIB40:2341832-2341881 | AO090023000894 |
| Chr2_A_oryzae_RIB40:2891094-2891143 | AO090003000296 |
| Chr2_A_oryzae_RIB40:2891094-2891143 | AO090003000295 |
| Chr5_A_oryzae_RIB40:2800716-2800765 | AO090120000053 |
| Chr6_A_oryzae_RIB40:568132-568181 | AO090020000492 |
| Chr6_A_oryzae_RIB40:568132-568181 | AO090020000493 |
| Chr3_A_oryzae_RIB40:755189-755238 | AO090023000297 |
| Chr5_A_oryzae_RIB40:4422681-4422730 | AO090113000160 |
| Chr2_A_oryzae_RIB40:1016938-1016987 | AO090001000406 |
| Chr4_A_oryzae_RIB40:461189-461238 | AO090012000185 |
| Chr5_A_oryzae_RIB40:1086479-1086528 | AO090701000481 |
| Chr2_A_oryzae_RIB40:2973126-2973175 | AO090003000323 |
| Chr5_A_oryzae_RIB40:4066771-4066820 | AO090113000007 |
| Chr5_A_oryzae_RIB40:4066771-4066820 | AO090113000008 |
| Chr3_A_oryzae_RIB40:2491251-2491300 | AO090003001591 |
| Chr3_A_oryzae_RIB40:2491251-2491300 | AO090120000195 |
| Chr3_A_oryzae_RIB40:2491251-2491300 | AO090120000196 |
| Chr3_A_oryzae_RIB40:2491251-2491300 | AO090023000944 |
| Chr3_A_oryzae_RIB40:2491251-2491300 | AO090023000945 |
| Chr1_A_oryzae_RIB40:3404907-3404956 | AO090005001138 |
| Chr1_A_oryzae_RIB40:3553979-3554028 | AO090005001083 |
| Chr2_A_oryzae_RIB40:1726978-1727027 | AO090001000764 |
| Chr2_A_oryzae_RIB40:1726978-1727027 | AO090001000656 |
| Chr2_A_oryzae_RIB40:3001608-3001657 | AO090003000333 |
| Chr2_A_oryzae_RIB40:3108100-3108149 | AO090003000378 |
| Chr2_A_oryzae_RIB40:6053945-6053994 | AO090003001482 |
| Chr3_A_oryzae_RIB40:65446-65495 | AO090023000023 |
| Chr3_A_oryzae_RIB40:750732-750781 | AO090023000293 |
| Chr3_A_oryzae_RIB40:750732-750781 | AO090023000292 |
| Chr3_A_oryzae_RIB40:1949633-1949682 | AO090023000744 |
| Chr3_A_oryzae_RIB40:1949633-1949682 | AO090023000743 |
| Chr4_A_oryzae_RIB40:3354238-3354287 | AO090102000436 |
| Chr4_A_oryzae_RIB40:4538292-4538341 | AO090003001591 |
| Chr4_A_oryzae_RIB40:4538292-4538341 | AO090120000195 |
| Chr4_A_oryzae_RIB40:4538292-4538341 | AO090120000196 |
| Chr4_A_oryzae_RIB40:4538292-4538341 | AO090023000944 |
| Chr4_A_oryzae_RIB40:4538292-4538341 | AO090023000945 |
| Chr6_A_oryzae_RIB40:2127636-2127685 | AO090038000565 |
| Chr6_A_oryzae_RIB40:2127636-2127685 | AO090038000564 |
| Chr6_A_oryzae_RIB40:2287511-2287560 | AO090038000514 |
| Chr6_A_oryzae_RIB40:2545717-2545766 | AO090038000421 |
| Chr6_A_oryzae_RIB40:3281982-3282031 | AO090038000154 |
| Chr6_A_oryzae_RIB40:4155808-4155857 | AO090138000019 |
| Chr7_A_oryzae_RIB40:290606-290655 | AO090011000102 |
| Chr7_A_oryzae_RIB40:1297081-1297130 | AO090011000517 |
| Chr8_A_oryzae_RIB40:1931936-1931985 | AO090010000549 |
| Chr8_A_oryzae_RIB40:1940357-1940406 | AO090010000546 |
| Chr8_A_oryzae_RIB40:2931790-2931839 | AO090010000183 |
| Chr6_A_oryzae_RIB40:3892379-3892428 | AO090138000132 |
| Chr2_A_oryzae_RIB40:4101558-4101607 | AO090003000747 |
| Chr2_A_oryzae_RIB40:3417349-3417398 | AO090701001090 |
| Chr3_A_oryzae_RIB40:1680995-1681044 | AO090023000640 |
| Chr2_A_oryzae_RIB40:5269063-5269112 | AO090003001165 |
| Chr5_A_oryzae_RIB40:3193430-3193479 | AO090120000202 |
| Chr5_A_oryzae_RIB40:3193430-3193479 | AO090120000201 |
| Chr6_A_oryzae_RIB40:787273-787322 | AO090020000417 |
| Chr2_A_oryzae_RIB40:273497-273546 | AO090001000118 |
| Chr3_A_oryzae_RIB40:229159-229208 | AO090023000086 |
| Chr5_A_oryzae_RIB40:595226-595275 | AO090701000676 |
| Chr5_A_oryzae_RIB40:1952495-1952544 | AO090701000146 |
| Chr3_A_oryzae_RIB40:3132742-3132791 | AO090026000728 |
| Chr3_A_oryzae_RIB40:3305752-3305801 | AO090026000659 |
| Chr3_A_oryzae_RIB40:3305752-3305801 | AO090026000658 |
| Chr4_A_oryzae_RIB40:4017456-4017505 | AO090102000204 |
| Chr5_A_oryzae_RIB40:870828-870877 | AO090701000561 |
| Chr5_A_oryzae_RIB40:870828-870877 | AO090701000562 |
| Chr2_A_oryzae_RIB40:5582696-5582745 | AO090003001289 |
| Chr2_A_oryzae_RIB40:5582696-5582745 | AO090003001290 |
| Chr6_A_oryzae_RIB40:2130828-2130877 | AO090038000562 |
| Chr6_A_oryzae_RIB40:2130828-2130877 | AO090038000563 |
| Chr1_A_oryzae_RIB40:3433357-3433406 | AO090005001124 |
| Chr1_A_oryzae_RIB40:3433357-3433406 | AO090005001126 |
| Chr5_A_oryzae_RIB40:1405259-1405308 | AO090701000351 |
| Chr6_A_oryzae_RIB40:2062716-2062765 | AO090038000590 |
| Chr1_A_oryzae_RIB40:967553-967602 | AO090009000362 |
| Chr3_A_oryzae_RIB40:2951123-2951172 | AO090026000801 |
| Chr2_A_oryzae_RIB40:5947448-5947497 | AO090003001431 |
| Chr3_A_oryzae_RIB40:75468-75517 | AO090023000027 |
| Chr5_A_oryzae_RIB40:13463-13512 | AO090701000902 |
| Chr3_A_oryzae_RIB40:3211164-3211213 | AO090026000698 |
| Chr1_A_oryzae_RIB40:4209783-4209832 | AO090005000826 |
| Chr3_A_oryzae_RIB40:880681-880730 | AO090023000346 |
| Chr3_A_oryzae_RIB40:880681-880730 | AO090023000345 |
| Chr1_A_oryzae_RIB40:6161237-6161286 | AO090005001653 |
| Chr3_A_oryzae_RIB40:3770666-3770715 | AO090026000479 |
| Chr5_A_oryzae_RIB40:963156-963205 | AO090701000527 |
| Chr5_A_oryzae_RIB40:963156-963205 | AO090701000526 |
| Chr1_A_oryzae_RIB40:4108822-4108871 | AO090005000866 |
| Chr1_A_oryzae_RIB40:4108822-4108871 | AO090005000867 |
| Chr2_A_oryzae_RIB40:5667476-5667525 | AO090003001319 |
| Chr5_A_oryzae_RIB40:2573233-2573282 | AO090124000046 |
| Chr8_A_oryzae_RIB40:2621131-2621180 | AO090011000531 |
| Chr8_A_oryzae_RIB40:2621131-2621180 | AO090023000203 |
| Chr8_A_oryzae_RIB40:2621131-2621180 | AO090001000763 |
| Chr8_A_oryzae_RIB40:2621131-2621180 | AO090012000948 |
| Chr2_A_oryzae_RIB40:3685816-3685865 | AO090003000596 |
| Chr2_A_oryzae_RIB40:758901-758950 | AO090001000758 |
| Chr1_A_oryzae_RIB40:2375882-2375931 | AO090005001521 |
| Chr4_A_oryzae_RIB40:3740166-3740215 | AO090102000291 |
| Chr4_A_oryzae_RIB40:3740166-3740215 | AO090102000290 |
| Chr8_A_oryzae_RIB40:893150-893199 | AO090103000145 |
| Chr6_A_oryzae_RIB40:914623-914672 | AO090020000367 |
| Chr1_A_oryzae_RIB40:5526486-5526535 | AO090005000356 |
| Chr5_A_oryzae_RIB40:4452205-4452254 | AO090113000177 |
| Chr8_A_oryzae_RIB40:2058691-2058740 | AO090010000496 |
| Chr1_A_oryzae_RIB40:187751-187800 | AO090009000066 |
| Chr1_A_oryzae_RIB40:971544-971593 | AO090009000363 |
| Chr1_A_oryzae_RIB40:3783569-3783618 | AO090005000988 |
| Chr3_A_oryzae_RIB40:4511189-4511238 | AO090026000213 |
| Chr3_A_oryzae_RIB40:3558743-3558792 | AO090026000557 |
| Chr5_A_oryzae_RIB40:2027693-2027742 | AO090701000120 |
| Chr5_A_oryzae_RIB40:1915011-1915060 | AO090701000157 |
| Chr1_A_oryzae_RIB40:1360891-1360940 | AO090009000513 |
| Chr2_A_oryzae_RIB40:2732127-2732176 | AO090003000240 |
| Chr6_A_oryzae_RIB40:3780859-3780908 | AO090138000173 |
| Chr3_A_oryzae_RIB40:4739925-4739974 | AO090026000136 |
| Chr6_A_oryzae_RIB40:1075876-1075925 | AO090020000301 |
| Chr4_A_oryzae_RIB40:2883012-2883061 | AO090102000609 |
| Chr2_A_oryzae_RIB40:6233694-6233743 | AO090003001549 |
| Chr2_A_oryzae_RIB40:6233694-6233743 | AO090003001548 |
| Chr6_A_oryzae_RIB40:789226-789275 | AO090020000416 |
| Chr1_A_oryzae_RIB40:3314838-3314887 | AO090005001174 |
| Chr1_A_oryzae_RIB40:3314838-3314887 | AO090005001175 |
| Chr2_A_oryzae_RIB40:5326619-5326668 | AO090003001188 |
| Chr8_A_oryzae_RIB40:1903776-1903825 | AO090010000563 |
| Chr1_A_oryzae_RIB40:828639-828688 | AO090009000309 |
| Chr5_A_oryzae_RIB40:2611208-2611257 | AO090124000028 |
| Chr7_A_oryzae_RIB40:1175940-1175989 | AO090011000466 |
| Chr1_A_oryzae_RIB40:3569611-3569660 | AO090005001078 |
| Chr2_A_oryzae_RIB40:1038096-1038145 | AO090001000411 |
| Chr3_A_oryzae_RIB40:3788688-3788737 | AO090026000472 |
| Chr3_A_oryzae_RIB40:3788688-3788737 | AO090026000471 |
| Chr3_A_oryzae_RIB40:1787809-1787858 | AO090023000681 |
| Chr2_A_oryzae_RIB40:1677360-1677409 | AO090001000636 |
| Chr2_A_oryzae_RIB40:1844840-1844889 | AO090001000699 |
| Chr8_A_oryzae_RIB40:557655-557704 | AO090103000276 |
| Chr1_A_oryzae_RIB40:469119-469168 | AO090009000175 |
| Chr8_A_oryzae_RIB40:1976655-1976704 | AO090010000529 |
| Chr7_A_oryzae_RIB40:1547979-1548028 | AO090701001274 |
| Chr3_A_oryzae_RIB40:3527734-3527783 | AO090026000569 |
| Chr3_A_oryzae_RIB40:3527734-3527783 | AO090026000568 |
| Chr2_A_oryzae_RIB40:1536365-1536414 | AO090001000586 |
| Chr3_A_oryzae_RIB40:2019103-2019152 | AO090023000775 |
| Chr3_A_oryzae_RIB40:2019103-2019152 | AO090023001013 |
| Chr2_A_oryzae_RIB40:2911140-2911189 | AO090003000301 |
| Chr3_A_oryzae_RIB40:4916831-4916880 | AO090701001140 |
| Chr3_A_oryzae_RIB40:4916831-4916880 | AO090026000078 |
| Chr5_A_oryzae_RIB40:3886030-3886079 | AO090120000448 |
| Chr6_A_oryzae_RIB40:3742351-3742400 | AO090138000188 |
| Chr2_A_oryzae_RIB40:2403577-2403626 | AO090003000115 |
| Chr5_A_oryzae_RIB40:2106923-2106972 | AO090701000095 |
| Chr5_A_oryzae_RIB40:2106923-2106972 | AO090701000094 |
| Chr8_A_oryzae_RIB40:2040052-2040101 | AO090010000505 |
| Chr1_A_oryzae_RIB40:4475165-4475214 | AO090005000730 |
| Chr2_A_oryzae_RIB40:3623423-3623472 | AO090003000572 |
| Chr5_A_oryzae_RIB40:1874737-1874786 | AO090701000175 |
| Chr1_A_oryzae_RIB40:6174700-6174749 | AO090005000101 |
| Chr1_A_oryzae_RIB40:6174700-6174749 | AO090005000100 |
| Chr5_A_oryzae_RIB40:4006469-4006518 | AO090120000492 |
| Chr2_A_oryzae_RIB40:1549108-1549157 | AO090001000590 |
| Chr6_A_oryzae_RIB40:87843-87892 | AO090020000685 |
| Chr8_A_oryzae_RIB40:2507151-2507200 | AO090010000313 |
| Chr8_A_oryzae_RIB40:2507151-2507200 | AO090010000312 |
| Chr1_A_oryzae_RIB40:5973017-5973066 | AO090005000170 |
| Chr4_A_oryzae_RIB40:2511529-2511578 | AO090012000967 |
| Chr4_A_oryzae_RIB40:4376945-4376994 | AO090102000065 |
| Chr1_A_oryzae_RIB40:2317922-2317971 | AO090005001540 |
| Chr5_A_oryzae_RIB40:2570163-2570212 | AO090124000047 |
| Chr7_A_oryzae_RIB40:143462-143511 | AO090011000043 |
| Chr1_A_oryzae_RIB40:1465415-1465464 | AO090009000556 |
| Chr3_A_oryzae_RIB40:3977486-3977535 | AO090026000405 |
| Chr4_A_oryzae_RIB40:1023393-1023442 | AO090012000416 |
| Chr2_A_oryzae_RIB40:5505376-5505425 | AO090003001589 |
| Chr7_A_oryzae_RIB40:2878975-2879024 | AO090206000114 |
| Chr5_A_oryzae_RIB40:1695644-1695693 | AO090701000245 |
| Chr4_A_oryzae_RIB40:791010-791059 | AO090012000320 |
| Chr4_A_oryzae_RIB40:1716081-1716130 | AO090012000676 |
| Chr6_A_oryzae_RIB40:3967733-3967782 | AO090138000107 |
| Chr6_A_oryzae_RIB40:1463470-1463519 | AO090020000141 |
| Chr2_A_oryzae_RIB40:3469616-3469665 | AO090003000517 |
| Chr7_A_oryzae_RIB40:1200389-1200438 | AO090011000531 |
| Chr7_A_oryzae_RIB40:1200389-1200438 | AO090023000203 |
| Chr7_A_oryzae_RIB40:1200389-1200438 | AO090001000763 |
| Chr7_A_oryzae_RIB40:1200389-1200438 | AO090012000948 |
| Chr3_A_oryzae_RIB40:314703-314752 | AO090023000125 |
| Chr3_A_oryzae_RIB40:1680093-1680142 | AO090023000641 |
| Chr4_A_oryzae_RIB40:1133711-1133760 | AO090012000451 |
| Chr5_A_oryzae_RIB40:3813504-3813553 | AO090120000422 |
| Chr5_A_oryzae_RIB40:3813504-3813553 | AO090120000421 |
| Chr5_A_oryzae_RIB40:3086764-3086813 | AO090120000157 |
| Chr7_A_oryzae_RIB40:2042434-2042483 | AO090011000800 |
| Chr2_A_oryzae_RIB40:5159760-5159809 | AO090003001123 |
| Chr4_A_oryzae_RIB40:354510-354559 | AO090701000959 |
| Chr1_A_oryzae_RIB40:4359724-4359773 | AO090701001036 |
| Chr3_A_oryzae_RIB40:544141-544190 | AO090023000216 |
| Chr4_A_oryzae_RIB40:1109245-1109294 | AO090012000444 |
| Chr1_A_oryzae_RIB40:6053793-6053842 | AO090701001052 |
| Chr3_A_oryzae_RIB40:4154125-4154174 | AO090026000338 |
| Chr3_A_oryzae_RIB40:4154125-4154174 | AO090026000339 |
| Chr4_A_oryzae_RIB40:361464-361513 | AO090012000150 |
| Chr4_A_oryzae_RIB40:1121225-1121274 | AO090012000447 |
| Chr4_A_oryzae_RIB40:1121225-1121274 | AO090012000448 |
| Chr5_A_oryzae_RIB40:4429316-4429365 | AO090113000164 |
| Chr2_A_oryzae_RIB40:4460731-4460780 | AO090003000871 |
| Chr1_A_oryzae_RIB40:994982-995031 | AO090009000371 |
| Chr1_A_oryzae_RIB40:994982-995031 | AO090009000370 |
| Chr3_A_oryzae_RIB40:1631392-1631441 | AO090023000623 |
| Chr6_A_oryzae_RIB40:412795-412844 | AO090020000555 |
| Chr4_A_oryzae_RIB40:4872931-4872980 | AO090166000120 |
| Chr6_A_oryzae_RIB40:1393013-1393062 | AO090020000169 |
| Chr1_A_oryzae_RIB40:1053044-1053093 | AO090009000395 |
| Chr1_A_oryzae_RIB40:5480736-5480785 | AO090005000374 |
| Chr3_A_oryzae_RIB40:3368243-3368292 | AO090026000633 |
| Chr1_A_oryzae_RIB40:1458121-1458170 | AO090009000553 |
| Chr2_A_oryzae_RIB40:4057850-4057899 | AO090003000729 |
| Chr5_A_oryzae_RIB40:4199455-4199504 | AO090113000063 |
| Chr6_A_oryzae_RIB40:1341488-1341537 | AO090020000191 |
| Chr3_A_oryzae_RIB40:4192364-4192413 | AO090026000326 |
| Chr3_A_oryzae_RIB40:4192364-4192413 | AO090026000325 |
| Chr1_A_oryzae_RIB40:6010399-6010448 | AO090005000157 |
| Chr1_A_oryzae_RIB40:6010399-6010448 | AO090005000158 |
| Chr1_A_oryzae_RIB40:6295674-6295723 | AO090005000053 |
| Chr3_A_oryzae_RIB40:2357300-2357349 | AO090023000900 |
| Chr3_A_oryzae_RIB40:1507610-1507659 | AO090023000571 |
| Chr5_A_oryzae_RIB40:1556097-1556146 | AO090701000301 |
| Chr1_A_oryzae_RIB40:3735201-3735250 | AO090005001008 |
| Chr1_A_oryzae_RIB40:3643045-3643094 | AO090005001045 |
| Chr7_A_oryzae_RIB40:826392-826441 | AO090011000327 |
| Chr4_A_oryzae_RIB40:2475815-2475864 | AO090012000951 |
| Chr8_A_oryzae_RIB40:1808499-1808548 | AO090010000603 |
| Chr2_A_oryzae_RIB40:5805496-5805545 | AO090003001373 |
| Chr2_A_oryzae_RIB40:5805496-5805545 | AO090003001374 |
| Chr4_A_oryzae_RIB40:1730760-1730809 | AO090012000681 |
| Chr8_A_oryzae_RIB40:50132-50181 | AO090701001280 |
| Chr3_A_oryzae_RIB40:3015763-3015812 | AO090026000776 |
| Chr4_A_oryzae_RIB40:3564287-3564336 | AO090102000353 |
