# Supplementary material for: Comprehensive investigation of the gene expression system regulated by an Aspergillus oryzae transcription factor XlnR using integrated mining of gSELEX-Seq and microarray data
Source: BMC Genomics. 2019 Jan 8;20:16. doi: 10.1186/s12864-018-5375-5 (PMC6323846; doi:10.1186/s12864-018-5375-5)
Supplement: Supplementary file 7 — Table S4. Correlation analyses between various factors related to AoXlnR binding and differential expression levels. (DOC 49 kb) [file 12864_2018_5375_MOESM7_ESM.doc]

**Supplementary Table 4. Correlation analyses between various factors related to AoXlnR binding and differential expression levels.**

| **Factor** | **Spearman’s rank correlation** | | **Pearson’s correlation** | | **The number of analyzed promoter** |
| --- | --- | --- | --- | --- | --- |
|  | **Correlation coefficient** | ***p*-value** | **Correlation coefficient** | ***p*-value** |  |
| **Detected Peak**  **Fold Enrichment** | 0.419 | 0.00220 | 0.335 | 0.0162 | 51 |
| **Detected Peak**  **Position** | 0.0434 | 0.763 | 0.0464 | 0.747 | 51 |
| **The number of GGCTGA** | 0.360 | 0.00192 | 0.519 | 3.04×10^-6^ | 72 |
| **The number of GGCTAA** | 0.369 | 0.00141 | 0.219 | 0.0647 | 72 |
| **The number of GGCTAG** | -0.0889 | 0.458 | 0.00965 | 0.936 | 72 |
| **The number of CGGNTAAW** | 0.228 | 0.0543 | 0.127 | 0.287 | 72 |
| **The number of TTAGSCTAA** | 0.338 | 0.00370 | 0.295 | 0.0120 | 72 |
| **Total Number of**  **Binding Sites** | 0.419 | 2.49×10^-4^ | 0.425 | 2.01×10^-4^ | 72 |
| **The coexistence of GGCTGA and**  **GGCTAA** | 0.515 | 3.72×10^-6^ | 0.573 | 1.41×10^-7^ | 72 |
| **The coexistence of GGCTGA and**  **GGCTAG** | 0.138 | 0.249 | 0.158 | 0.184 | 72 |
| **The coexistence of GGCTAA and**  **GGCTAG** | 0.0632 | 0.589 | 0.115 | 0.336 | 72 |
