# Supplementary material for: Comprehensive investigation of the gene expression system regulated by an Aspergillus oryzae transcription factor XlnR using integrated mining of gSELEX-Seq and microarray data
Source: BMC Genomics. 2019 Jan 8;20:16. doi: 10.1186/s12864-018-5375-5 (PMC6323846; doi:10.1186/s12864-018-5375-5)
Supplement: Supplementary file 8 — Figure S4. Summits of peaks detected using gSELEX-Seq in the promoter regions of xynF1, xynG1, xynG2, xylA, celA, celB, celC and celD. gSELEX-Seq peaks were detected using MACS (v1.4.2). Blue squares indicate the summits of the peaks. Red square frames indicate canonical XlnR binding motifs. (DOCX 278 kb) [file 12864_2018_5375_MOESM8_ESM.docx]

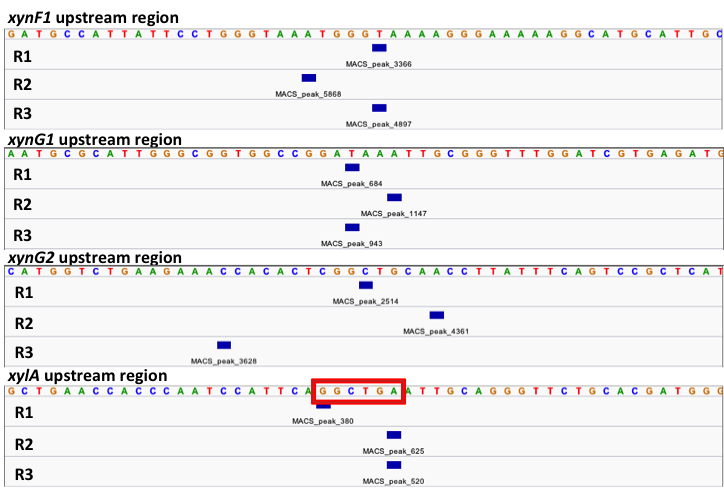


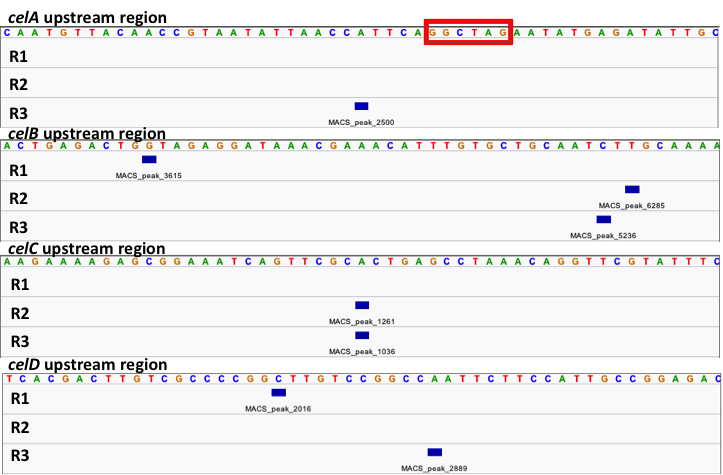


**Supplementary Fig. 4. Summits of peaks detected using gSELEX-Seq in the promoter regions of *xynF1*, *xynG1, xynG2*, *xylA*, *celA*, *celB*, *celC* and *celD*.**

gSELEX-Seq peaks were detected using MACS (v1.4.2). Blue squares indicate the summits of the peaks.

Red square frames indicate canonical XlnR binding motifs.
